# Understand the Specific Regio- and Enantioselectivity of Fluostatin Conjugation in the Post-Biosynthesis

**DOI:** 10.3390/biom10060815

**Published:** 2020-05-26

**Authors:** Yuanqi Wang, Changsheng Zhang, Yi-Lei Zhao, Rosalinda Zhao, Kendall N. Houk

**Affiliations:** 1 State Key Laboratory of Microbial Metabolism, Joint International Research Laboratory of Metabolic and Developmental Sciences, School of Life Sciences and Biotechnology, Shanghai Jiao Tong University, 800 Dongchuan Road, Shanghai 200240, China; wyq1996@sjtu.edu.cn; 2 Key Laboratory of Tropical Marine Bio-resource and Ecology, Guangdong Key Laboratory of Marine Materia, RNAM Center for Marine Microbiology, South China Sea Institute of Oceanology, Chinese Academy of Sciences, 164 West Xingang Road, Guangzhou 510301, China; czhang@scsio.ac.cn; 3 Department of Chemistry and Biochemistry, University of California, Los Angeles, Los Angeles, CA 90095, USA; rzhao2022@bwscampus.com (R.Z.); houk@chem.ucla.edu (K.N.H.)

**Keywords:** fluostatin, conjugation, regioselectivity, stereoselectivity, π–π stacking interaction

## Abstract

Fluostatins, benzofluorene-containing aromatic polyketides in the atypical angucycline family, conjugate into dimeric and even trimeric compounds in the post-biosynthesis. The formation of the C–C bond involves a non-enzymatic stereospecific coupling reaction. In this work, the unusual regio- and enantioselectivities were rationalized by density functional theory calculations with the M06-2X (SMD, water)/6–311 + G(d,p)//6–31G(d) method. These DFT calculations reproduce the lowest energy C1-(R)-C10′-(S) coupling pathway observed in a nonenzymatic reaction. Bonding of the reactive carbon atoms (C1 and C10′) of the two reactant molecules maximizes the HOMO–LUMO interactions and Fukui function involving the highest occupied molecular orbital (HOMO) of nucleophile ***p*-QM** and lowest unoccupied molecular orbital (LUMO) of electrophile **FST_2_^−^** anion. In particular, the significant π–π stacking interactions of the low-energy pre-reaction state are retained in the lowest energy pathway for C–C coupling. The distortion/interaction–activation strain analysis indicates that the transition state (**TScp-I**) of the lowest energy pathway involves the highest stabilizing interactions and small distortion among all possible C–C coupling reactions. One of the two chiral centers generated in this step is lost upon aromatization of the phenol ring in the final difluostatin products. Thus, the π–π stacking interactions between the fluostatin 6-5-6 aromatic ring system play a critical role in the stereoselectivity of the nonenzymatic fluostatin conjugation.

## 1. Introduction

**Figure 1:**
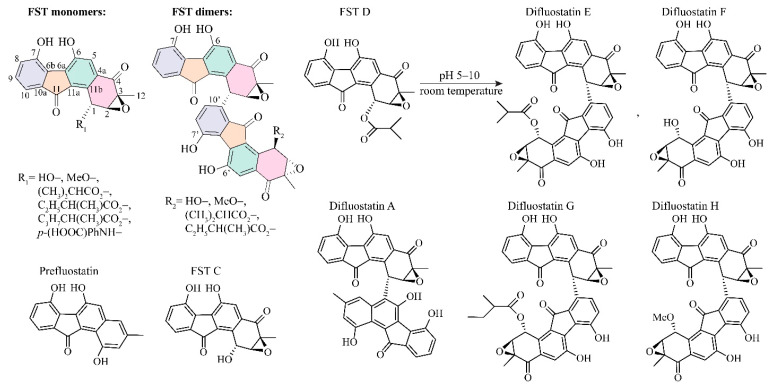
Structures of several discovered fluorostatin (FST) monomers and dimers, in which FST D undergoes spontaneous conjugation and yields Difluostatins E and F in aqueous solution.

Fluostatins (FSTs) are a family of benzofluorene-containing angucyclines produced in marine species *Micromonospora rosaria* and certain Streptomyces strains [1]. They generally possess a unique 6-5-6-6 ring skeleton (Figure 1) and are potential antitumor molecules, like the analogs kinamycin [2,3], lomaiviticin [4,5,6], and nenestatin [7]. As shown in Figure 1 and Figure S1, FSTs A–E compounds were discovered as secondary metabolites in Streptomyces species [8,9]; F–H were produced by heterologous expression of a transformation-assisted recombination (TAR) gene cluster [10]; I–L were obtained from *Micromonospora rosaria* SCSIO N160 and *Streptomyces coelicolor* YF11 [11,12]; M–Q were discovered from *Streptomyces* sp. PKU-MA00045 [13]; and R–S were generated from *Streptomyces coelicolor* by heterologous expression of the fluostatin biosynthesis gene cluster [1]. Based on the preliminarily pharmacological tests, FSTs A–B can inhibit the bioactivity of dipeptidyl peptidase III [9], C exhibits moderate inhibition against many human tumor cells [8], and F–H show mild antibacterial activities [10]. In light of the druggability of Lomaiviticin A, many biosynthetic chemists have begun to pursue the dimeric fluostatins for the amplification of therapeutic potential [12].

Unexpectedly, the recently biochemical and biosynthetic experiments indicated that fluostatin dimerization occurs without any auxiliary proteins [1]. The structural characterization of the end-products indicates there is regio- and enantioselectivity in the non-enzymatic reaction—the stereogenic center C1 generated in the dimerization exclusively favors the *R*-configuration (Figure 1). This observation is very unusual, because the stereochemical C–C couplings in biosynthesis are usually related to chirality generating enzymes such as aldolases [14,15,16], ketolases [17,18], Diels–Alderases [19,20,21,22,23], and others [24]. Previously, FlsQ1 was hypothetically annotated as the master in the fluostatin’s conjugate reaction due to its homology with a known dimerase in the NmrA protein family of actinorhodin biosynthesis) [25,26]. However, the knockout of FlsQ1 did not stop fluostatin’s conjugation, and the later extensive investigation revealed that only one protein related to the conjugation was FlsH in the *fls* gene cluster [1].

Interestingly, FlsH is indeed a serine esterase that catalyzes the deacylation of acyl-fluostatin toward low-cytotoxic C1-hydroxyl-fluostatin. Deacylation of fluostatin occurs spontaneously in the absence of FlsH, but at a much slower rate (FlsH rate enhancement: ~230-fold), leading to *para-*quinone methide (***p*-QM**) instead. The in vitro spontaneous reaction did generate certain conjugated fluostatin compounds observed in *Streptomyces albus* J1074, including difluostatin A and fluostatin S. Therefore, the dimeric fluostatins can be considered as non-metabolites occurring the post-biosynthesis [1].

In this work, the reaction mechanisms for the pure cell-free nonenzymatic process of FST D towards Difluostatin E are illustrated computationally using the density functional theory. Our calculations have demonstrated the energy superiority of the observed enantioselective C1-C10′ coupling. Electronic structure analysis shows how the molecular orbital interactions in the transition state control the regioselectivity of the conjugate addition. Intriguingly, the non-covalent interaction between the two 6-5-6 aromatic rings of fluorene scaffolds regulates the stereochemistry through favorable π–π interactions as interpreted by the spatial electron density gradient IGM descriptor (IGM: Independent Gradient Model). Mechanistic interrogation indicates the C–C coupling to be non-rate-determining with a very low energy barrier due to excellent alignment of HOMO–LUMO orbitals of the electrophile and nucleophile (HOMO, the Highest Occupied Molecular Orbital; LUMO, the Lowest Unoccupied Molecular Orbital). The π–π stacking is prevalent in both aromatic host–guest assemblies [27] and chemical reactions [28,29], but rare in the two reacting aromatic systems due to that the *sp*^2^-*sp*^2^ cross-coupling destroys the parallelism and aromaticity of both the two reacting partners.

Moreover, the π–π stacking is often controlled primarily by dispersion interactions, whereas secondary orbital interactions, electrostatic polarization, and charge transfer mingle with dispersion between the two reacting π systems in the pre-reaction state and transition states [30]. In this work, we computationally compared the activation electronic energy of the Michael addition step calculated using the Distortion/Interaction–Activation Strain (DIAS) method to estimate the magnitude of π–π stacking stabilization, which proved the stronger interaction in the transition state than the pre-reaction state. Since the similar π–π stacking also takes place between the polycyclic aromatic natural product (e.g., Lomaiviticin A) and double-stranded DNA target, the physicochemical basis of the fluostatin conjugate reaction casts light on polycyclic aromatic dimerization in natural product biosynthesis and pharmacology.

## 2. Computation Details 

All stationary point structures were optimized with the M06-2X functional [31] and the 6-31G(d) basis set in Gaussian 09 revision C.01 [32]. Vibrational frequency analyses were performed at the same level of theory to ensure local minima or first-order saddle points, and the free energies were calculated at 298 K (standard condition). In addition, the intrinsic reaction coordinates (IRC) calculations [33] and relaxed potential surface scans were carried out to identify transition states and immediate reactants and products. The electronic energies were updated with the single point calculation of the larger basis sets, i.e., M06-2X/6-311 + G(d,p). To mimic different pH conditions, the deacyloxylation was assessed with all three possible forms, i.e., neutral, monoanion, and dianion. The regiochemistry for the conjugate reaction between nucleophile and electrophile was studied by the frontier molecular orbital (FMO) analysis and with Hirshfeld charges [34,35,36] and with Fukui functions (FF) [37,38,39,40] at the M06-2X/6-311+G(d,p) level of theory in the Multiwfn 3.6 software package [41]. For a given molecular system, FF ($f^{-}$/$f^{+}$) was calculated using electron density of three states:

$$f^{-}\left(r\right)=\rho_{N}\left(r\right)-\rho_{N-1}\left(r\right),$$

$$f^{+}\left(r\right)=\rho_{N+1}\left(r\right)-\rho_{N}\left(r\right),$$

where *N* is the number of electrons in the current molecular system. The N−1 and N+1 states share the same molecular geometry as the N state. For nucleophiles, $f^{-}$ is the reactivity descriptor, while for electrophiles, $f^{+}$ is the descriptor. Atoms with larger FFs tend to have higher reactivities. To clarify the FF distribution at different atoms, the condensed FF ($f_{A}^{-}$/$f_{A}^{+}$) is used, which can be derived from atomic charges (in this case Hirshfeld atom charges were used):

$$f_{A}^{-}=q_{N-1}^{A}-q_{N}^{A},$$

$$f_{A}^{+}=q_{N}^{A}-q_{N+1}^{A}.$$

After inspecting FMO composition and FF, the eight possible conjugate pathways were calculated with the above DFT method. In addition, the composition of both FMOs are further analyzed by Hirshfeld partitioning scheme [42,43,44]. To understand the non-covalent interaction during the reaction course, we used distortion/interaction-activation strain energy decomposition [45,46] and IGM analysis [47]. All the above calculations incorporate the SMD implicit solvation model [48] for aqueous solution (SMD, universal Solvation Model based on solute electron Density). Basis set superposition error (BSSE) energy was corrected during the distortion-interaction analyses with the counterpoise method [49]. Isosurface maps were produced using VMD 1.9.3 program [50] based on outputs from the Multiwfn calculations.

## 3. Results and Discussion

### 3.1. The Reaction Energy Profile of Fluostatin Dimerization

**Figure 2:**
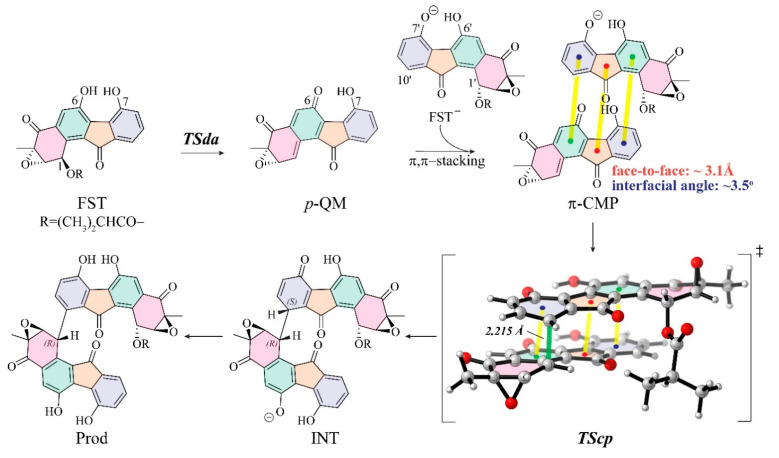
The proposed reaction mechanism for fluostatin conjugation in this work. (FST, fluostatin D; FST^−^, fluostatin anion; TSda, transition state of deacyloxylation; *p*-QM, *para*-quinone methide; π-CMP, the low-energy π–π stacking pre-reaction complex; TScp, the low-energy transition state of C-C coupling; INT, the immediate product of TScp; Prod, Difluostatin E.)

As proposed in the experimental study [1], the conjugate reaction proceeds via a two-step reaction mechanism: (1) a 1,6-elimination process of fluostatin monomer leads to a transient *p*-quinone methide (***p*-QM**) via transition states of deacyloxylation (**TSda**); (2) then the resulting ***p*-QM** conjugates with another molecule of fluostatin toward the dimer via transition states of C–C coupling (**TScp**) (Figure 2). In Huang’s work, the formation of ***p*-QM** from fluostatin has been characterized in detail with a series of in vivo and in vitro experiments with various nucleophiles. Originally, *p*-hydroxyl benzyl acetate was found to undergo spontaneous acetyl elimination to yield a *para*-quinone methide [51]. Then, the compatible in situ generation of *p*-QMs from stable p-hydroxybenzyl alcohol turned out to be the preparation of transient *p*-QM species in organic synthesis [52]. It was thus conceivable whether hydrolysis of the ester linkage at C1 is a prerequisite for the formation of *p*-QMs. The isotopic experiment of FST with H_2_^18^O ruled out the hydrolysis pathway because the ^18^O incorporation was detected in the C1-hydroxyl-fluostatin (FST C, Figure 1) by LC–MS analysis (see Supplementary Figure 117 in [1]). This indicates that FST C was formed by the Michael addition of *p*-QM and H_2_^18^O, rather than by the simple hydrolysis of FST D. However, the regio- and enantio-selectivity of the following conjugate reaction was unclear. We studied FST D as the representative fluostatin model (FST) in the calculations of the non-enzymatic spontaneous reaction.

**Figure 3:**
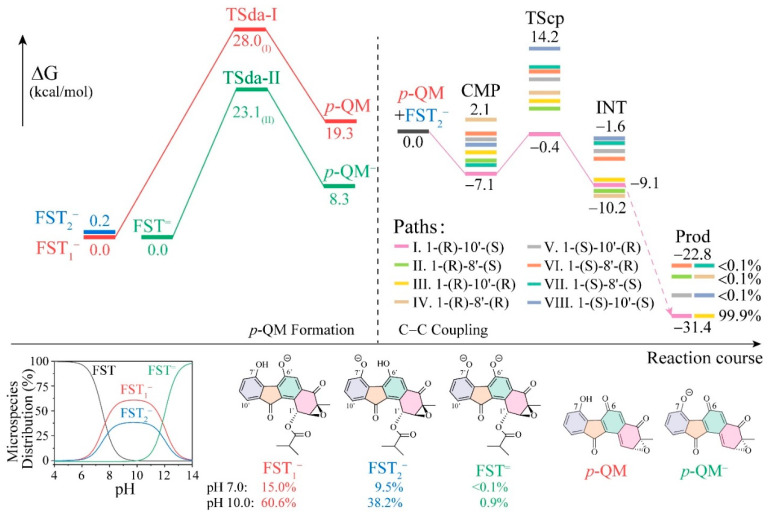
The free energy profile of the *p*-QM formation and C–C coupling (top), and the conjugated acid-base microspecies distribution (bottom).

The approximation of pK_a_ by cheminformatics leads to the prediction of a mixture of neutral, monoanion, and dianion under the experimental condition (pH 3–10). The pK_a_ predictor in the Marvin software suite predicted that the micro-mode pK_a_ values for the O6 and O7 phenol groups are 7.70 and 7.90 at 298 K, and that the macro-mode pK_a_ values of in silico titration are 7.49 and 12.06 (towards dianion) for the fluostatin conjugated acid–base system [53,54]. Another online pK_a_ prediction platform developed by Luo et al. gave the even lower pK_a_ prediction of 6.39 in aqueous solution, using the ensemble machine learning method (RMSE = 1.76, r^2^ = 0.918) [55], the algorithm integrated 35,000 experimental pK_a_ values of the iBond database [56]. Compared to phenol (pK_a_ = 9.95), fluostatin has a low pK_a_ value due to the dramatic intramolecular hydrogen bonding between the two phenoxyl groups (the [OH…O]^−^ distance in the monoanions: 1.50 Å) and strong substitution effect of two electron-withdrawing carbonyl groups [57]. According to the microspecies distribution analysis in the Marvin software suite, fluostatin readily deprotonates at approximately pH 5.0 or lower (Figure 3, the bottom inset). At pH 7.0, the monoanionic abundances of FST_1_^−^ and FST_2_^−^ are ~15.0% and ~9.5%, respectively. At pH 10.0 the monoanionic populations rise to 60.6% and 38.2%. In the meantime, the partition of the neutral form drops to 0.5% and that of the dianion FST^=^ increases to 0.6%. With an increased pH value, both monoanions will deprotonate to dianion FST^=^.

The deacyloxylation was calculated with the different protonation states of fluostatin, as shown in Figure 3. The transition states **TSda-I** and **TSda-II** are for deacyloxylation from FST_1_^−^ and FST^=^; the barriers are 28.0 and 23.1 kcal/mol, respectively. The neutral form leads to neither transition state nor stable product in the relaxed scan calculation (see Figure S2). The monoanion route is favored, even more so because the concentration ratio of [FST_1_^−^]: [FST^=^] is one-thousand-fold. The calculated results are in good agreement with the experimental observation, where the acyl FSTs were stable in aprotic organic solvents and under acidic conditions (pH < 4), but the reaction significantly accelerates under basic conditions (pH > 6) (see Supplementary Figure 116 in [1]).

The deacyloxylation serves to generate the active species ***p*-QM** [58,59,60], which is the key to formation of pre-reaction complex for the following C–C coupling [61,62]. The formation of ***p*-QM** is the rate-determining step of the dimerization. In previous experiments artificial methylation of either O6 or O7 prohibited the dimerization. 

Regarding possible nucleophiles in conjugate addition to ***p*-QM**, both monoanions and dianion are expected to be nucleophiles, but the monoanions is more abundant and **FST_1_^−^** more nucleophilic than **FST_2_^−^** (see Table S1).

The second step (i.e., the C–C coupling) determines the regio- and enantioselectivity of the conjugate dimerization. In this stage, ***p*-QM** and **FST_2_^−^** form a series of reactive complexes before conjugation, and the pre-reaction complexes then undergo the crucial C–C coupling transition state (**TScp**). As the coupling finishes, ***p*-QM** and **FST_2_^−^** conjugate covalently by the newly formed C–C bond, which connects the two new chiral centers in the dimers. 

Due to the multiple possibilities of C–C coupling combination and chirality, different pre-reaction complexes may exist between **FST_2_^−^** and ***p*-QM**. The non-covalent interaction between the two reacting partners includes electrostatic interaction of neutral molecule and anion, and π–π interactions of the two aromatic rings. Several pre-reactive complexes were computed and analyzed by frontier orbital analysis and Fukui’s function—the details will be illustrated in the following sections.

The top eight C–C coupling pathways have been scrutinized to identify the most favorable pathway. The binding affinity of the eight reactive complexes ranges from −7.1 to 2.1 kcal/mol, and the relative free energies of **TScp** are calculated in a range of −0.4 to 14.2 kcal/mol relative to the free energy zero point of **FST_2_^−^** plus ***p*-QM**. The pre-reaction states (**CMP**) and transition states (**TScp**) of pathway I toward the C1-(R)-C10′-(S) immediate product (**INT**) possess the lowest free energies with an activation barrier of only 6.7 kcal/mol. The two corresponding structures, **π-CMP** and **TScp**, are very stable, suggesting that its π–π stacking interaction is predominant and overcomes the destabilization caused by the *sp*^2^ to *sp*^3^ change.

Finally, the conversion of the dienone to phenol takes place after the C–C conjugation, facilitated by solvent acid and base catalysts. The relative free energies of the plausible conjugate anions were calculated to be lower by 22.8 to 31.4 kcal/mol than those of **FST_2_^−^** plus ***p*-QM**. 

Reaction pathway **I** is the most favorable and leads to >99.9% of the final enantiomer. The origins of this high selectivity are analyzed below.

### 3.2. Electronic Structure Analysis for Regioselectivity of the Conjugate Addition

The electronic structures of **FST_2_^−^** and ***p*-QM** are used here to analyze the C–C coupling between electrophile and nucleophile in the Michael addition. The phenoxyl group of **FST_2_^−^** is electron-donating, significantly enhancing reactivity at *ortho* (C8′) and *para* (C10′) positions. In case of ***p*-QM**, due to the existence of a sizeable conjugate system of three carbonyl groups, the carbon atoms with *sp*^2^ configuration within the system (C1, C4, C4a, C5, C6, C6a, C11, C11a) are all electrophilic. To better understand regioselectivity, we illustrated the quantitative measure of the frontier molecular orbital (FMO) composition analysis and Fukui function (FF).

**Figure 4:**
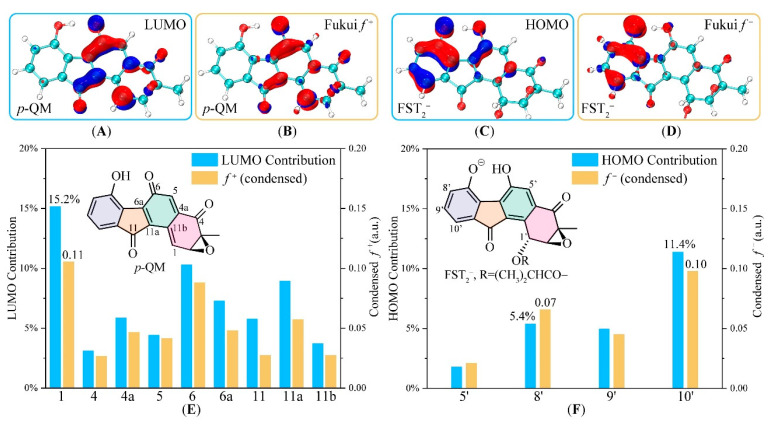
Prediction of the C–C coupling regioselectivity by frontier molecular orbitals (FMO) and Fukui function (FF). (**A**,**B**) Isosurface of the LUMO and Fukui $f^{+}$ of electrophile ***p*-QM**, respectively; (**C**,**D**) those of the HOMO and Fukui $f^{-}$ of nucleophile **FST_2_^−^** (FMO: isovalue = ±0.05 a.u., FF: isovalue = ±0.002 a.u.); (**E**,**F**) Comparison of FMO coefficients and condensed FF for ***p*-QM** and **FST_2_^−^**, respectively.

FMO and FF are useful concepts and is widely applied for determining stereoselectivity and reactivity in chemical reactions [63,64,65,66,67,68,69]. Figure 4 shows the HOMO of the **FST_2_^−^** (nucleophile) and LUMO of ***p*-QM** (electrophile). These are significantly delocalized within the two conjugate molecules. As shown in Figure 4, HOMO-LUMO analysis and Fukui functions give essentially identical predictions. The LUMO of ***p*-QM** mainly populates O6, O11, C1, and C4, while HOMO of **FST_2_^−^** expands over two 6-membered rings, with the majority populated on the terminal phenol ring.

The orbital interaction in the Michael addition is likely to be dominant by the HOMO–LUMO interaction. Isosurface of Fukui function mimicked the special electron density changes in the reaction. The electron flow to nucleophile ***p*-QM** increases its electron density near C1, C6, C6a, C11, and C11a, in perpendicular direction to the conjugated aromatic ring. The electron flow from **FST_2_^−^** is also perpendicular to its conjugated aromatic ring. This is consistent with the expectation that **FST_2_^−^** HOMO electrons would transfer to the LUMO of the recipient molecule, in a face-to-face fashion through the vertical space. The HOMO–LUMO orbital interaction between the nucleophile and electrophile is consistent with π–π stacking interaction between the two 6-5-6 fused aromatic rings. 

Taken together, the FMO and FF orbital analyses predicted that the most nucleophilic sites in **FST_2_^−^** go in the order of C10′ > C8′ > others and the most electrophilic sites in ***p*-QM** go in the order of C1 > C6 > C11a > others. Because of the steric repulsion for any C–C coupling between the ring-centered atoms C6 and C11a, only edged sites were accessible in searching for the pre-reaction complexes. 

### 3.3. Insight into π–π Stacking’s Influence on Regio- and Stereoselectivity

**Figure 5:**
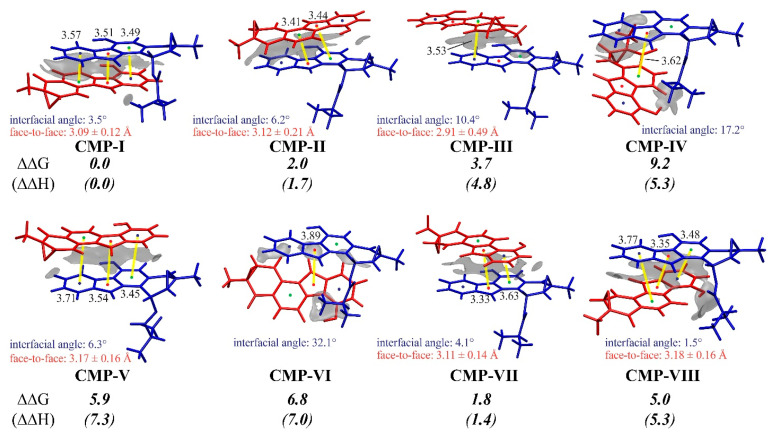
The complex structures of all pre-reaction states towards the eight possible diastereoisomers (INT). The weak intermolecular interactions were indicated as gray isosurfaces between *p*-QM (red) and **FST_2_^−^** (blue) by IGM. Relative free energies and enthalpies (in kcal/mol) were calculated to the CMP-I.

As both the FMO and Fukui Function analyses provided electronic evidence for the HOMO–LUMO interaction between the **FST_2_^−^** and ***p*-QM**, we explored the factors favoring a specific transition state. Figure 5 showed the eight pre-reaction states corresponding to the eight diastereoisomers. Among them, CMP-I presented an absolute advantage in the molecular interaction and led to the major chiral product observed in the biosynthesis. To understand the energetic preference of the non-covalent interaction, we visualized the weak interactions with the state-of-the-art IGM analysis [70], and the face-to-face distance, center-to-center distance, and dihedral angles of the two 6-5-6 aromatic rings in Figure 5.

As shown in Figure 3 and Figure 5, pathways **I** to **VIII** were named by the rank of **TScp** energies, corresponding to the relative yields of final enantiomer products. **CMP-I** and **V** switched the up-and-down parallel position of the **FST_2_^−^** and ***p*-QM** molecules, toward the 1-(R)-10′-(S) and 1-(S)-10′-(R) configuration of diastereoisomer products. Similarly, **CMP-II**/**VI**, **III**/**VIII**, and **IV**/**VII** are paired 6-5-6 parallels. **CMP-I**, **III**, **V**, and **VIII** were related to the C10′ attacking, whereas others were related to the C8′. The lowest energy **CMP-I** and **TScp-I** were connected in the dominant pathway **I** to the major product **Prod-I&III**. In general, the π–π stacking effect plays a central role in the complexations, and the stability is related to how the two molecules orient each other. The two 6-5-6 aromatic rings of **CMP-I**, **II**, **V**, **VII**, and **VIII** are reasonably close and parallel to each other, resulting in large contact areas and, more robust stabilization from π–π stacking effect. By contrast, **CMP-III**, **IV**, and **VI** have interfacial angles more massive than 10° and less parallelism, showing a weaker dispersion between the two molecules. 

Besides π–π stacking interactions, the steric effect may also be significant for the complexation. In **CMP-VIII**, the overhanging side chain at C1′ in **FST_2_^−^** has a steric hindrance with ***p*-QM**, resulting in tightened positioning of the side chain and considerably elevated the total energy. In the case of **CMP-V**, the epoxide group of ***p*-QM** comes in contact with **FST_2_^−^** making ***p*-QM** slightly bend away from **FST_2_^−^** and the face-to-face distance longer.

Taking all factors into consideration, **CMP-I** stands out as the most stable pre-reaction state as it employs the best orientation for intermolecular interaction and avoids repulsive steric hindrance. The beneficial stacking interaction makes **CMP-I** predominant and promotes pathway **I** as well.

### 3.4. Distortion/Interaction-Activation Strain Analysis of Transition States (TScp)

**Figure 6:**
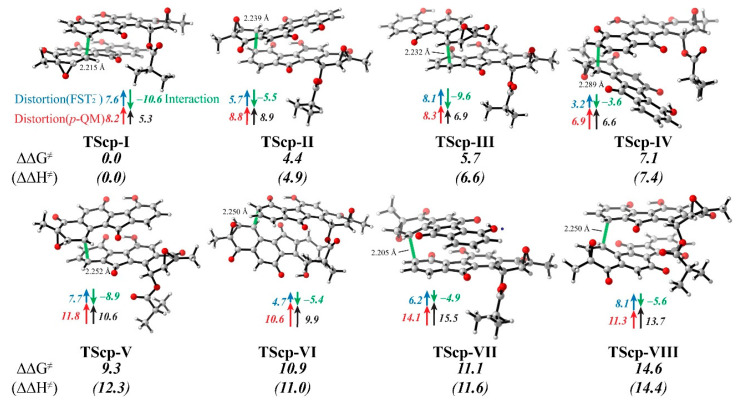
The calculated structures of TScp. (Bond lengths of the partially forming C–C bond are labeled, and the total energy change between pre-reaction state (CMPs) and transition states (TScp) are marked in black color, which consists of changes in the distortion energy of **FST_2_^−^** (blue), the distortion energy of ***p*-QM** (red), and the additional interaction energy for the C–C coupling.)

Even though the π–π stacking interaction in the most stable pre-reaction complex significantly boosts the binding affinity between the **FST_2_^−^** and ***p*-QM** molecules by ~7.2 kcal/mol in free energy (Figure 3), it does not guarantee that the beneficial intermolecular interaction is always retained in the transition states (**TScp**). To further examine how the intermolecular interaction affects the C–C coupling, we used the distortion–interaction analysis to quantify the intramolecular strain of the two monomers and the intermolecular interaction along the reaction coordinate, with reference to their corresponding pre-reaction states **CMP-I** to **VIII** (Figure 6 and Figures S3 and S4 in Supplementary Material). 

In the pre-reaction states (**CMPs**), the intramolecular distortions **FST_2_^−^** and ***p*-QM** were neglected (Figure S7). However, in the transition states (**TScp**), the two reactive carbon atoms of the nucleophile and electrophile both transform from *sp*^2^ to *sp*^3^ configuration, leading to a dramatical distortion in the range of 3.2 to 14.1 kcal/mol (**FST_2_^−^**: avg. 6.4 kcal/mol, and ***p*-QM**: avg. 10.0 kcal/mol, respectively). On the other hand, the forming C–C bond brings the additional interaction between the two motifs **FST_2_^−^** and ***p*-QM** by 3.6–10.6 kcal/mol. Intriguingly, the most intensive interaction of 10.6 kcal/mol was observed in **TScp-I**, where the distortion energies were moderate (7.6 and 8.2 kcal/mol for **FST_2_^−^** and ***p*-QM**, respectively).

In the cases of **TScp-V**, **VI**, **VII**, and **VIII**, the ***p*-QM** distortion energies increase by 10.6 kcal/mol or more and the approaching C1 site in *S-*configuration. In contrast, the ***p*-QM** distortion energies increase by 8.8 kcal/mol or less in **TScp-I**, **II**, **III**, and **IV** with the C1 chirality in *R-*configuration. It was evident that the striking difference was caused by the nearby epoxy group, which also caused the internal strain within ***p*-QM**. Given that the epoxy groups clash the counterpart monomer in the *S*-configuration of TScps (V–VIII, with nonbonded C…O distances of less than 3.0 Å), one may argue that the steric effect of the epoxy group in ***p*-QM** determines the C1(*R*)-configuration of the major product. (Figures S5 and S6)

If the motif **FST_2_^−^** has significant steric hindrance with ***p*-QM**, such as in **TScp-VIII**, the internal strain will cause a greater increase in distortion energy of **FST_2_^−^**. However, the distortion energy of **FST_2_^−^** stems from the strain energy at either the C10′ or C8′ atoms as electronic structure change rapidly during the temporary transition from *sp*^2^ to *sp*^3^. In general, if C8′ and C10′ are less displaced from their original location relative to C7′, C9′ and C10a’, the distortion energy should be smaller. Therefore, the magnitude by which the distortion energy of **FST_2_^−^** cannot be easily attributed to a single reason.

The increase in interaction energy is of primary focus. Because the structures of **TScps** mostly resemble **π-CMPs**, the weak interaction (dispersion) does not dominate. Instead, molecular orbital interaction became the main factor. With a close distance between the C10′/C8′ and C1 atoms, the frontier molecular orbitals of both molecules (primarily HOMO in **FST_2_^−^** and LUMO in ***p*-QM**) interacted with each other to form the C–C bond in **INT**. The rearrangement of molecular orbitals leads to a more stable electronic structure. This stabilizing effect competes with the distortion energies, but together they determine the activation barrier. Interestingly, interaction energy increase is generally more significant in C1–C10′ combinations (**TScp-I**, **III**, **V**, and **VIII**) than C1–C8′ combinations (**TScp-II**, **IV**, **VI**, and **VII**).

The overall activation barrier is a sum of the distortion and interaction energies between the two molecules. Reaction path **I** has both the lowest energy **TScp** and **CMP**, due to moderate distortion and superior orbital–orbital interaction (see Figure S7), and consequently generates only FST dimers with the observed chirality. In the final step, the quinone methide structure in ***p*-QM** isomerizes into a stable phenol structure, and the C–C coupling at C10′ or C8′ in **FST_2_^−^** leads to the conjugate products.

## 4. Conclusions

Although most stereospecific conjugations in natural product biosynthesis are catalyzed by particular proteins in their functional gene cluster, fluostatin dimerization is spontaneous and non-enzymatic. We have carried out extensive DFT calculations on the reaction mechanism and successfully determined the regioselectivity and enantioselectivity of the C–C conjugation. (1) The deacyloxylation step is rate-determining for the conjugate dimerization. Both monoanionic and bianionic deacyloxylations are possible under experimental conditions. (2) The resulting *para-*quinone methide is highly reactive. Both the frontier molecular orbital interactions and Fukui function analyses suggest that the C1 position is the most electrophilic. (3) The monoanion **FST_2_^−^** acts as the nucleophile with the highest electron-donating characteristic at the C8′ and C10′ positions. (4) The π–π stacking interaction of the two 6-5-6 aromatic rings steers the formation of pre-reaction states, which possesses the best parallel-displaced orientation with a face-to-face distance of 3.0 Å. The strong intermolecular interaction promotes the complexation of the two reactive species and alignment of the HOMO–LUMO molecular orbitals between the nucleophile and electrophile in a way that favors on stereoisomer. (5) The distortion/interaction-activation strain analysis on the C–C coupling course suggests that the HOMO–LUMO interaction stays in the lowest energy pathway from the pre-reaction state to the transition state. The *R*-C1 configuration in the conjugate products is likely related to the steric effect of the epoxy group and such a small hindrance was found to be critical for stereochemical regulation in butanolide heterodimerization as well [30]. Accordingly, we predict that non-epoxide pre-fluostatin might conjugate in a different fashion of regio- and enantioselectivity; for example, difluostatin A is a heterodimer by C–C linkage through C1-C5′ [12]. The unique π-conjugate systems interact with each other to form the sturdy π–π stacking conformation, very similar to the base–base stacking in B-type DNA double helix [71,72,73], layer–layer stacking in graphene [74,75,76,77], and heterocyclic molecules [78,79]. Short face-to-face distance, parallelism (~0° interfacial angle), multiple center-to-center overlaps, and minimal steric effect contribute to the predominant pre-reaction complex (π-**CMP-I**) and transition state (**TScp-I**). The C1-(R)-C10′-(S) coupling pathway is superior and explains why only one enantiomer of fluostatin dimer with *R* C1 chirality is obtained in the non-enzymatic experiments and post-biosynthesis.

## Supplementary Materials

The following are available online at https://www.mdpi.com/2218-273X/10/6/815/s1, Figure S1: The fluostatin family members; Figure S2: Transition state searching for deacyloxylation; Figure S3: Distortion/interaction-activation strain analysis for the pre-reaction complexes; Figure S4: Potential energy surface contours for TScps searching; Figure S5: Epoxy contacts in TScps; Figure S6: Epoxy contacts in CMPs; Figure S7: Energy decomposition analysis along intrinsic reaction coordinate; Table S1: Frontier molecular orbital and Fukui analyses of FST monoanions.
