# Supplementary material for: Understand the Specific Regio- and Enantioselectivity of Fluostatin Conjugation in the Post-Biosynthesis
Source: Biomolecules. 2020 May 26;10(6):815. doi: 10.3390/biom10060815 (PMC7355926; doi:10.3390/biom10060815)
Supplement: Supplementary file 1 [file biomolecules-10-00815-s001.pdf]

Supplementary Materials

Figure S1. The fluostatin family members --S2

Figure S2. Transition state searching for deacyloxyation---S3

Figure S3. Distortion/Interaction-Activation Strain analysis for the reactive complexes ---------------------S4

Figure S4. Potential energy surface contours for TScp searching in the eight reaction paths-----------------S5

Figure S5. Epoxy contacts in TScps---S6

Figure S6. Epoxy contacts in CMPs---S7

Table S1. FMO composition and FF analysis of FST_1^- and FST_2^----S8

Geometries of the 36 compounds discussed in the paper---S9–72

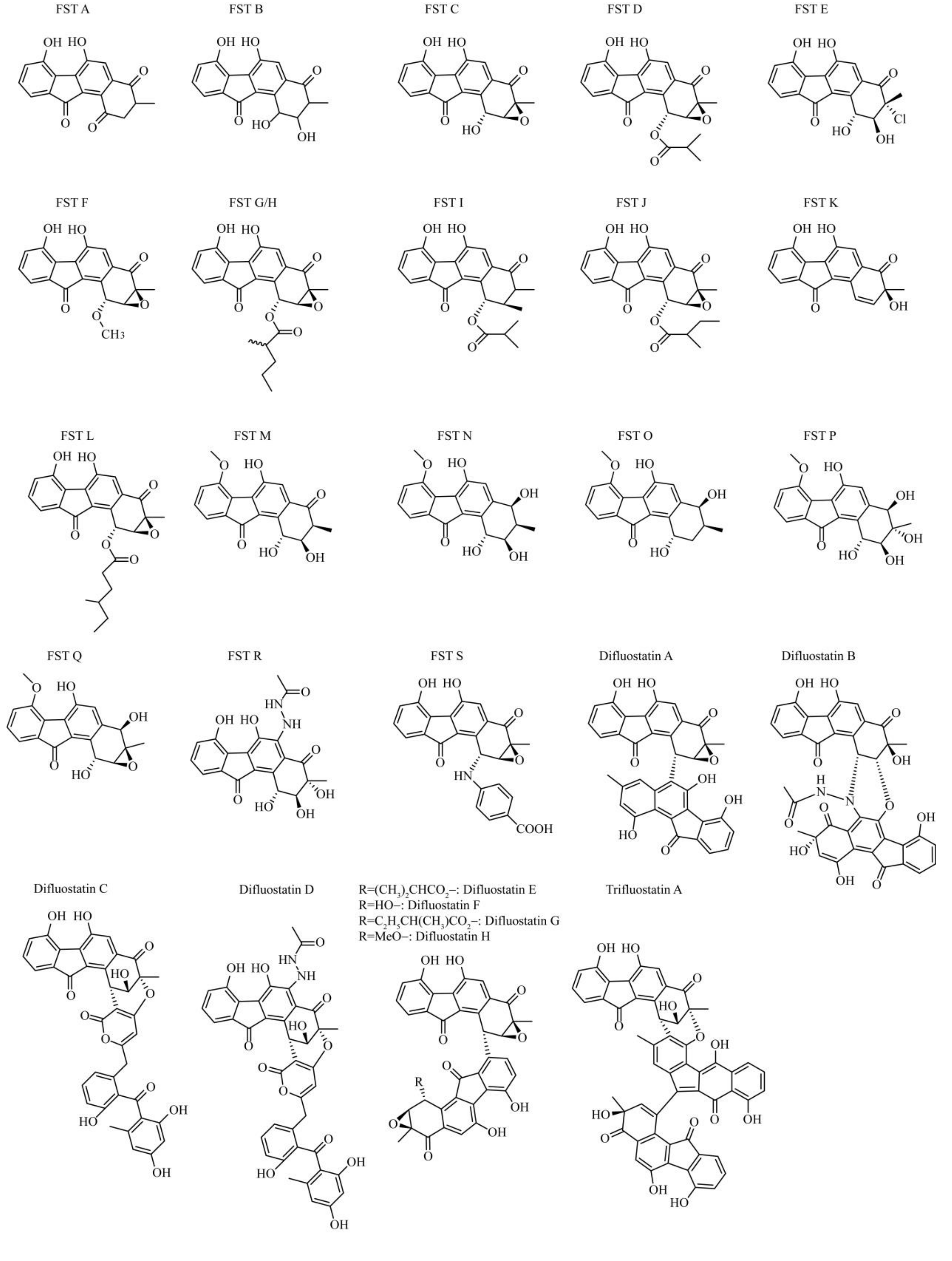

Figure S1. The fluostatin family members, including 19 monomers, eight dimers and one trimer.

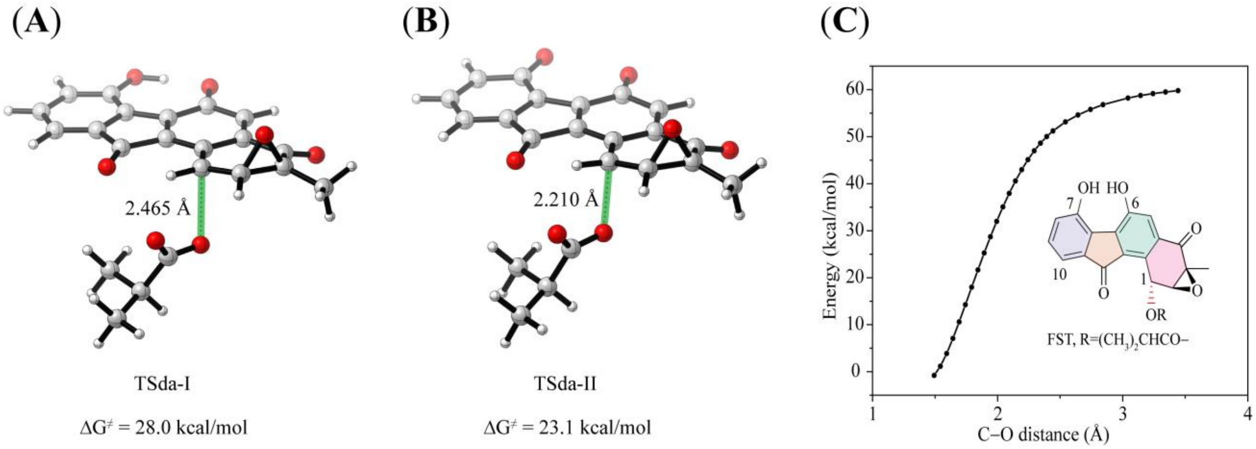

Figure S2. Transition state searching for deacyloxyation. (**A**) TSda-I of FST$^{-}_{I}$; (**B**) TSda-II of FST$^{=}$; and (**C**) the C-O elongation of neutral form FST (no transition state).

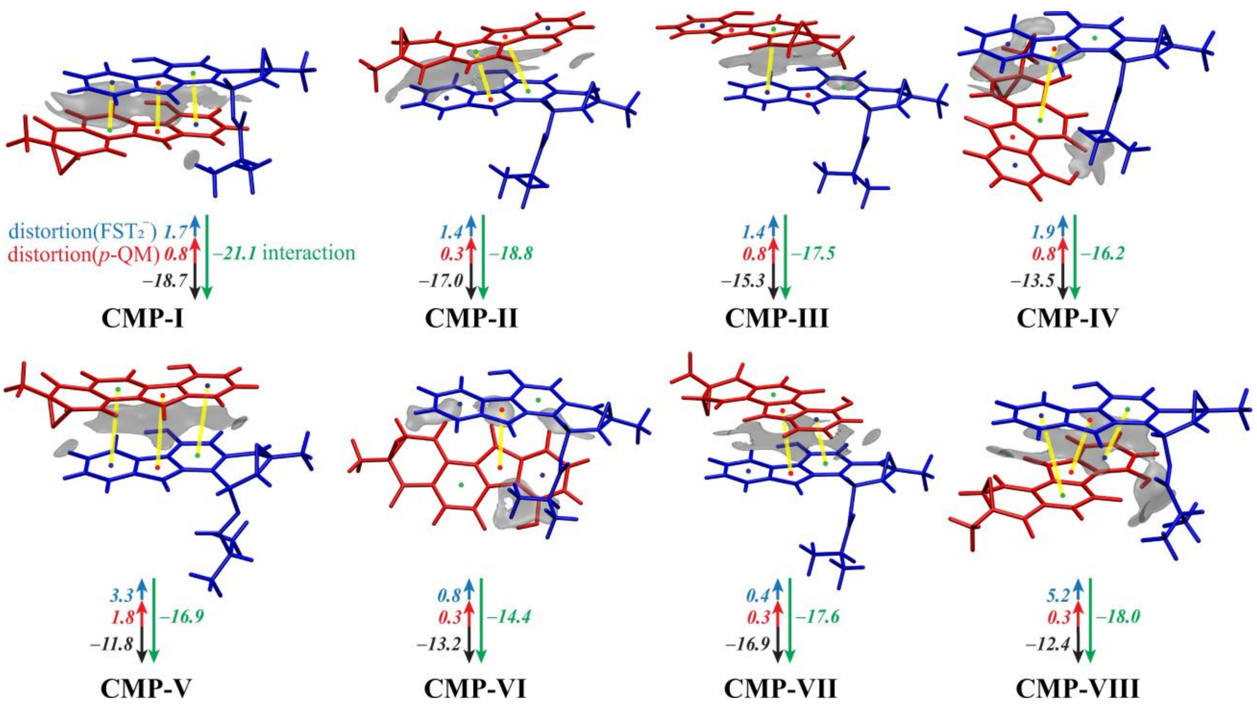

Figure S3. Distortion-interaction analysis for the reactive complexes.

*Total energy change between free (**FST$_2^-$** + ***p*-QM**) and pre-reaction state (**CMPs**) are marked in black color, which consists of changes in the distortion energy of **FST$_2^-$** (blue) and ***p*-QM** (red), and the interaction energy (green).

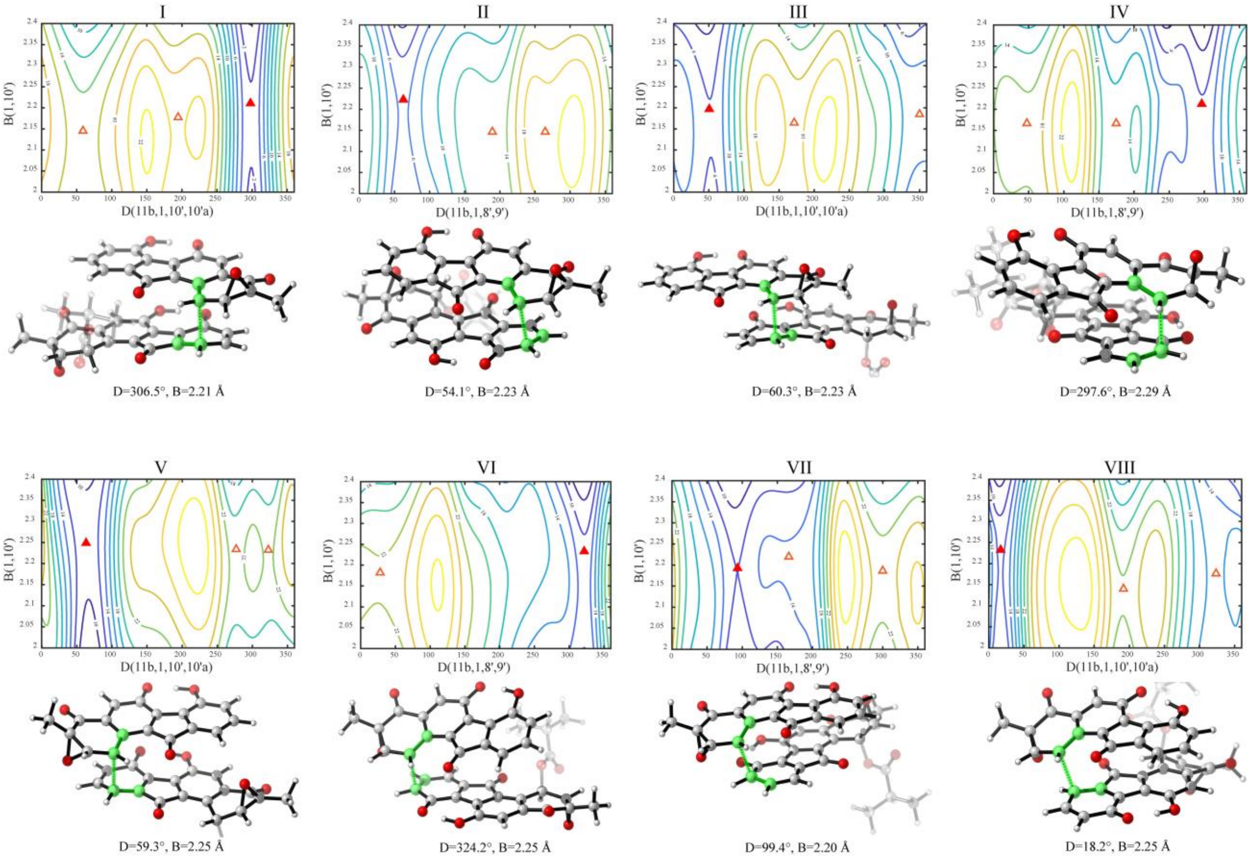

Figure S4. TScp potential energy surface contours of the eight reaction paths.

***** PES were scanned with the dihedral angle marked in green and the newly formed carbon-carbon bond denoted by dash lines. Multiple transition states (red triangles) exist in each path and the transition state with lowest energy (filled red triangle) is optimized. The optimized transition state structures are shown below each PES.

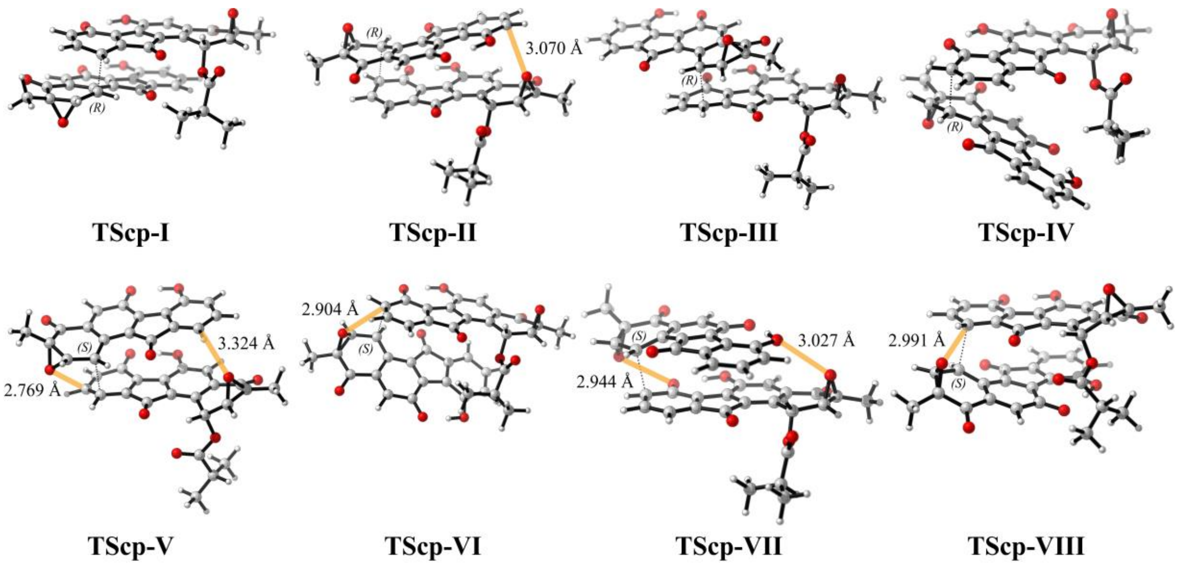

Figure S5. Epoxy contacts in TScps, represented by the shortest distances of the epoxy oxygen atoms to the heavy C/N/O atoms of its counterpart monomer.

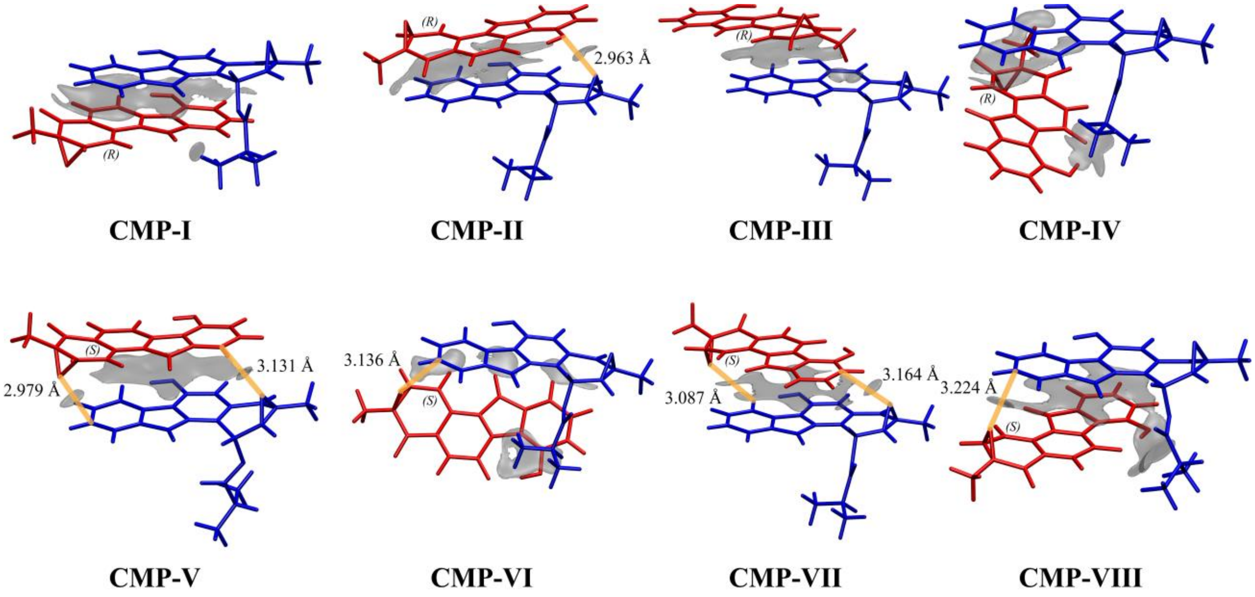

Figure S4. Epoxy contacts in CMPs, represented by the shortest distances of the epoxy oxygen atoms to the heavy C/N/O atoms of its counterpart monomer.

Table S1. FMO composition and FF analysis of FST_1^- and FST_2^-

Descriptor	C5′	C8′	C9′	C10′
FST_1^- HOMO composition	6.71%	1.44%	4.90%	3.92%
FST_2^- HOMO composition	1.79%	5.38%	4.96%	11.38%
FST_1^- FF (f^-)	0.075	0.020	0.036	0.028
FST_2^- FF (f^-)	0.021	0.066	0.045	0.098

Geometries of the compounds discussed in the paper:

1. M06-2X results for FST

Center Number	Atomic Number	Atomic Type	Coordinates (Angstroms) X	Y	Z
1	6	0	-3.724078	-1.984118	-0.100416
2	6	0	-2.553784	-1.258270	-0.080783
3	6	0	-2.502988	0.148834	0.000732
4	6	0	-3.702887	0.854495	0.063160
5	6	0	-4.907550	0.117408	0.045814
6	6	0	-4.925479	-1.262933	-0.033040
7	1	0	-3.706593	-3.067079	-0.164210
8	1	0	-5.833150	0.682081	0.096423
9	1	0	-5.876096	-1.785193	-0.043725
10	6	0	-0.269652	-0.582443	-0.075926
11	6	0	1.152792	-0.527401	-0.070125
12	6	0	1.762101	0.778470	-0.028805
13	6	0	0.991936	1.902498	0.025413
14	6	0	-0.425298	1.803819	0.056848
15	6	0	-1.065789	0.539154	0.000584
16	1	0	1.445102	2.889822	0.020984
17	6	0	-1.172510	-1.794949	-0.132150
18	8	0	-0.823949	-2.953489	-0.204639
19	8	0	-3.841180	2.197308	0.141703
20	1	0	-2.971609	2.638563	0.127904
21	8	0	-1.175551	2.879256	0.110051
22	6	0	3.248598	0.920050	-0.197160
23	6	0	1.929750	-1.659688	-0.125098
24	1	0	1.464155	-2.626099	-0.298128
25	6	0	3.380896	-1.612741	0.090352
26	6	0	4.096101	-0.302318	0.047135
27	8	0	3.746502	1.977793	-0.510691
28	8	0	3.761318	-0.912189	1.280511
29	1	0	3.952390	-2.511657	-0.114971
30	6	0	5.544856	-0.220754	-0.325077
31	1	0	5.643924	-0.017192	-1.393898
32	1	0	6.025448	0.586830	0.232930
33	1	0	6.038123	-1.165906	-0.090563
34	1	0	-0.660897	3.711716	0.123015

```
Final Gibbs Free Energy = Single Point Energy + Thermal correction to Gibbs Free Energy

SCF Done:  E(RM062X) =  -1375.63768943     A.U. after   16 cycles
SMD-CDS (non-electrostatic) energy       (kcal/mol) =       9.17
(included in total energy above)
Thermal corrections calculated under M06-2X/6-31G*:
M06-2X/6-31G*:
Zero-point correction=                           0.365613 (Hartree/Particle)
 Thermal correction to Energy=                    0.390377
 Thermal correction to Enthalpy=                  0.391322
 Thermal correction to Gibbs Free Energy=         0.311817
```

2. M06-2X results for FST_1^-

Center Number	Atomic Number	Atomic Type	X	Y	Z
1	6	0	-3.489068	2.220599	-0.798259
2	6	0	-2.613033	1.180496	-0.550330
3	6	0	-3.032152	-0.080855	-0.098283
4	6	0	-4.388841	-0.326159	0.117341
5	6	0	-5.287737	0.729296	-0.135483
6	6	0	-4.851326	1.972000	-0.581301
7	1	0	-3.132441	3.184840	-1.146435
8	1	0	-6.345192	0.545702	0.030736
9	1	0	-5.581153	2.754999	-0.761151
10	6	0	-0.704444	-0.231186	-0.295335
11	6	0	0.572178	-0.774067	-0.260720
12	6	0	0.668262	-2.104944	0.193101
13	6	0	-0.465691	-2.829561	0.559778
14	6	0	-1.782298	-2.296701	0.504558
15	6	0	-1.848945	-0.951735	0.063827
16	1	0	-0.345133	-3.852433	0.905417
17	6	0	-1.126358	1.152708	-0.684962
18	8	0	-0.407918	2.076585	-1.032044
19	8	0	-4.866306	-1.509895	0.550267
20	8	0	-2.826846	-2.991936	0.841002
21	6	0	1.994848	-2.753379	0.337559
22	6	0	1.763479	0.055010	-0.643709
23	1	0	1.546124	0.615184	-1.556851
24	6	0	3.024729	-0.742183	-0.857409
25	6	0	3.169689	-2.128179	-0.370821
26	8	0	2.163089	-3.763500	1.003738
27	8	0	2.887260	-1.832994	-1.750845
28	1	0	3.934099	-0.154215	-0.978806
29	6	0	4.517015	-2.721881	-0.084097
30	1	0	4.748822	-2.632735	0.980404
31	1	0	4.529881	-3.781727	-0.351614
32	1	0	5.282618	-2.196832	-0.659472
33	1	0	-4.066249	-2.156689	0.683330
34	8	0	1.996240	1.003578	0.417909
35	6	0	2.435066	2.227101	0.060078
36	8	0	2.771652	2.502380	-1.072488
37	6	0	2.425183	3.176728	1.235241
38	1	0	2.911172	2.650446	2.064826
39	6	0	3.186960	4.452608	0.904093
40	1	0	3.198800	5.112883	1.775551
41	1	0	4.220905	4.237953	0.619765
42	1	0	2.705720	4.983659	0.077199
43	6	0	0.970080	3.468581	1.629443
44	1	0	0.424717	2.550020	1.862870
45	1	0	0.954142	4.114120	2.512007
46	1	0	0.450226	3.985295	0.816039

Coordinates (Angstroms)

```
Final Gibbs Free Energy = Single Point Energy + Thermal correction to Gibbs Free Energy

Single Point Energy calculated under M06-2X/6-311+G**:
SCF Done:  E(RM062X) =  -1375.18442782     A.U.
 SMD-CDS (non-electrostatic) energy       (kcal/mol) =       9.04
 (included in total energy above)
Thermal corrections calculated under M06-2X/6-31G*:
Zero-point correction=                           0.352103 (Hartree/Particle)
 Thermal correction to Energy=                    0.376092
 Thermal correction to Enthalpy=                  0.377036
 Thermal correction to Gibbs Free Energy=         0.299099
```

3. M06-2X results for FST_2^-

Center Number	Atomic Number	Atomic Type	X	Y	Z
1	6	0	-3.492463	2.214191	-0.801207
2	6	0	-2.615543	1.175981	-0.554206
3	6	0	-3.034468	-0.084793	-0.100437
4	6	0	-4.394888	-0.375623	0.133766
5	6	0	-5.287115	0.710571	-0.129293
6	6	0	-4.855419	1.950130	-0.576180
7	1	0	-3.144143	3.180607	-1.150950
8	1	0	-6.346196	0.532204	0.037731
9	1	0	-5.589648	2.730700	-0.754376
10	6	0	-0.700245	-0.225517	-0.299840
11	6	0	0.572981	-0.773710	-0.262888
12	6	0	0.677741	-2.103848	0.182299
13	6	0	-0.461532	-2.829651	0.548071
14	6	0	-1.744916	-2.275727	0.487588
15	6	0	-1.849762	-0.944805	0.057677
16	1	0	-0.349782	-3.854021	0.890424
17	6	0	-1.129739	1.155750	-0.693156
18	8	0	-0.412069	2.077552	-1.048237
19	8	0	-4.824795	-1.531245	0.555362
20	8	0	-2.812172	-3.011960	0.839665
21	6	0	2.000401	-2.755094	0.324195
22	6	0	1.765022	0.062524	-0.638776
23	1	0	1.549734	0.613148	-1.558417
24	6	0	3.034698	-0.725398	-0.835750
25	6	0	3.181659	-2.114178	-0.358516
26	8	0	2.158881	-3.781673	0.967299
27	8	0	2.917298	-1.809269	-1.740226
28	1	0	3.941219	-0.129674	-0.938680
29	6	0	4.527995	-2.702611	-0.057268
30	1	0	4.743382	-2.622881	1.011433
31	1	0	4.550943	-3.759518	-0.335259
32	1	0	5.298888	-2.167345	-0.615895
33	1	0	-3.679882	-2.428622	0.734470
34	8	0	1.971249	1.014594	0.421065
35	6	0	2.410662	2.239735	0.063968
36	8	0	2.758859	2.510529	-1.065510
37	6	0	2.387335	3.191773	1.236527
38	1	0	2.871262	2.669742	2.070035
39	6	0	3.145020	4.470400	0.906474
40	1	0	3.149240	5.132575	1.776539
41	1	0	4.181366	4.259694	0.628071
42	1	0	2.665676	4.997505	0.075948
43	6	0	0.928755	3.477481	1.622054
44	1	0	0.385927	2.556925	1.853404
45	1	0	0.905576	4.123621	2.504003
46	1	0	0.411360	3.991628	0.805566

```
Final Gibbs Free Energy = Single Point Energy + Thermal correction to Gibbs Free Energy

Single Point Energy calculated under M06-2X/6-311+G**:
SCF Done:  E(RM062X) =  -1375.18344723     A.U.
 SMD-CDS (non-electrostatic) energy       (kcal/mol) =       9.03
 (included in total energy above)

Thermal corrections calculated under M06-2X/6-31G*:
Zero-point correction=                           0.351564 (Hartree/Particle)
 Thermal correction to Energy=                    0.375595
 Thermal correction to Enthalpy=                  0.376539
 Thermal correction to Gibbs Free Energy=         0.298512
```

4. M06-2X results for FST=

Center Number	Atomic Number	Atomic Type	Coordinates (Angstroms) X	Y	Z
1	6	0	-3.477254	2.136458	-0.794275
2	6	0	-2.661992	1.055369	-0.520915
3	6	0	-3.096511	-0.210527	-0.072809
4	6	0	-4.503386	-0.437899	0.106861
5	6	0	-5.327132	0.715601	-0.183127
6	6	0	-4.855129	1.940937	-0.610069
7	1	0	-3.061215	3.080030	-1.133797
8	1	0	-6.396409	0.568678	-0.045243
9	1	0	-5.555106	2.749690	-0.804415
10	6	0	-0.745270	-0.323202	-0.266648
11	6	0	0.563673	-0.794595	-0.248630
12	6	0	0.740289	-2.111068	0.214138
13	6	0	-0.358948	-2.870141	0.602915
14	6	0	-1.719608	-2.424341	0.571867
15	6	0	-1.882837	-1.070106	0.102749
16	1	0	-0.191341	-3.883955	0.957767
17	6	0	-1.186060	1.045434	-0.652372
18	8	0	-0.482425	1.988633	-0.999345
19	8	0	-5.060615	-1.528197	0.483171
20	8	0	-2.660962	-3.203203	0.949106
21	6	0	2.091701	-2.696518	0.336408
22	6	0	1.708612	0.087457	-0.650448
23	1	0	1.453766	0.649825	-1.551023
24	6	0	3.000325	-0.651820	-0.894455
25	6	0	3.221102	-2.025601	-0.404931
26	8	0	2.327163	-3.693298	1.007152
27	8	0	2.895572	-1.752496	-1.780817
28	1	0	3.878088	-0.022481	-1.040118
29	6	0	4.602155	-2.553124	-0.149317
30	1	0	4.857226	-2.443937	0.908038
31	1	0	4.659804	-3.613498	-0.408978
32	1	0	5.326710	-1.996822	-0.748250
33	8	0	1.934048	1.038509	0.416137
34	6	0	2.339348	2.271342	0.061963
35	8	0	2.661384	2.563253	-1.071811
36	6	0	2.314835	3.216015	1.241455
37	1	0	2.805071	2.693014	2.070509
38	6	0	3.060418	4.503566	0.918710
39	1	0	3.063510	5.159165	1.793829
40	1	0	4.097257	4.303467	0.634011
41	1	0	2.573487	5.033554	0.094464
42	6	0	0.854600	3.485746	1.632263
43	1	0	0.323300	2.558580	1.864220
44	1	0	0.826226	4.131080	2.514764
45	1	0	0.328711	3.994080	0.817469

```
Final Gibbs Free Energy = Single Point Energy + Thermal correction to Gibbs Free Energy

Single Point Energy calculated under M06-2X/6-311+G**:
SCF Done:  E(RM062X) =  -1374.69445688     A.U.
 SMD-CDS (non-electrostatic) energy       (kcal/mol) =       9.48
 (included in total energy above)

Thermal corrections calculated under M06-2X/6-31G*:
Zero-point correction=                           0.340302 (Hartree/Particle)
 Thermal correction to Energy=                    0.364150
 Thermal correction to Enthalpy=                  0.365094
 Thermal correction to Gibbs Free Energy=         0.287702
```

5. M06-2X results for TSda-I (from FST_1^-)

Center Number	Atomic Number	Atomic Type	Coordinates (Angstroms) X	Y	Z
1	6	0	3.494653	2.271040	0.843823
2	6	0	2.659367	1.202071	0.603700
3	6	0	3.104963	-0.027489	0.087650
4	6	0	4.454298	-0.193207	-0.204111
5	6	0	5.320699	0.894361	0.040595
6	6	0	4.857409	2.095685	0.551235
7	1	0	3.110484	3.204727	1.241887
8	1	0	6.373783	0.759705	-0.186865
9	1	0	5.558942	2.905324	0.723259
10	6	0	0.799333	-0.283304	0.390318
11	6	0	-0.498541	-0.889214	0.393398
12	6	0	-0.577980	-2.223624	-0.175833
13	6	0	0.527725	-2.879690	-0.607139
14	6	0	1.862522	-2.290604	-0.534508
15	6	0	1.926516	-0.922732	-0.036940
16	1	0	0.449610	-3.873440	-1.037563
17	6	0	1.185582	1.112076	0.815855
18	8	0	0.448759	1.985605	1.228340
19	8	0	4.996677	-1.324927	-0.708585
20	8	0	2.867275	-2.927286	-0.903287
21	6	0	-1.922339	-2.860199	-0.372984
22	6	0	-1.601495	-0.266611	0.898896
23	1	0	-1.533502	0.725107	1.328475
24	6	0	-2.886391	-0.982641	1.045125
25	6	0	-3.085320	-2.303626	0.407252
26	8	0	-2.081046	-3.785664	-1.144904
27	8	0	-2.788230	-2.196981	1.800170
28	1	0	-3.764456	-0.381215	1.259215
29	6	0	-4.447735	-2.811560	0.040845
30	1	0	-4.665294	-2.581282	-1.005089
31	1	0	-4.494882	-3.895327	0.175827
32	1	0	-5.199632	-2.337468	0.675145
33	8	0	-2.689420	1.080003	-0.855276
34	6	0	-2.791275	2.225821	-0.323554
35	8	0	-3.032437	2.441220	0.892368
36	6	0	-2.529273	3.429298	-1.242421
37	1	0	-2.846677	3.138687	-2.250137
38	6	0	-3.286170	4.676484	-0.803175
39	1	0	-3.097461	5.506376	-1.492357
40	1	0	-4.366200	4.499574	-0.771295
41	1	0	-2.966574	4.985301	0.196972
42	6	0	-1.016970	3.681773	-1.267017
43	1	0	-0.471820	2.780313	-1.566867
44	1	0	-0.768256	4.484466	-1.969089
45	1	0	-0.665849	3.978600	-0.272105
46	1	0	4.277681	-1.999846	-0.826090

```
Final Gibbs Free Energy = Single Point Energy + Thermal correction to Gibbs Free Energy

Single Point Energy calculated under M06-2X/6-311+G**:
SCF Done:  E(RM062X) =  -1375.13280663     A.U.
 SMD-CDS (non-electrostatic) energy       (kcal/mol) =       9.74
 (included in total energy above)

Thermal corrections calculated under M06-2X/6-31G*:
Zero-point correction=                           0.348881 (Hartree/Particle)
 Thermal correction to Energy=                    0.373912
 Thermal correction to Enthalpy=                  0.374856
 Thermal correction to Gibbs Free Energy=         0.292085

Low frequencies ---  -61.3674   -5.2083   -0.0002    0.0006    0.0009    3.2875
 Low frequencies ---   10.4420   17.3613   27.6630
```

6. M06-2X results for TSda-II (from FST$^{=}$)

Center Number	Atomic Number	Atomic Type	X	Y	Z
1	6	0	3.500819	2.168598	0.800461
2	6	0	2.696423	1.079821	0.549054
3	6	0	3.142455	-0.171958	0.073064
4	6	0	4.538879	-0.384436	-0.154406
5	6	0	5.357292	0.786382	0.112514
6	6	0	4.879635	1.996264	0.562428
7	1	0	3.085327	3.103685	1.162906
8	1	0	6.422989	0.658022	-0.064269
9	1	0	5.570150	2.817099	0.737363
10	6	0	0.809543	-0.354267	0.357771
11	6	0	-0.519562	-0.879367	0.365245
12	6	0	-0.680358	-2.191618	-0.211161
13	6	0	0.396155	-2.894419	-0.650372
14	6	0	1.770926	-2.396339	-0.591217
15	6	0	1.932462	-1.034876	-0.062262
16	1	0	0.265855	-3.882358	-1.082490
17	6	0	1.224056	1.030735	0.739692
18	8	0	0.503738	1.948565	1.106253
19	8	0	5.097331	-1.463416	-0.548358
20	8	0	2.698232	-3.111694	-1.003319
21	6	0	-2.046982	-2.771272	-0.382650
22	6	0	-1.593998	-0.189897	0.879054
23	1	0	-1.443539	0.736580	1.416356
24	6	0	-2.906160	-0.857489	1.050723
25	6	0	-3.172469	-2.165052	0.416687
26	8	0	-2.266023	-3.693572	-1.147667
27	8	0	-2.852598	-2.071145	1.808979
28	1	0	-3.755370	-0.218675	1.275659
29	6	0	-4.561842	-2.616301	0.076093
30	1	0	-4.784982	-2.390679	-0.969784
31	1	0	-4.656847	-3.695074	0.226232
32	1	0	-5.283262	-2.100880	0.713753
33	8	0	-2.398521	1.112332	-0.709040
34	6	0	-2.574446	2.284864	-0.237704
35	8	0	-2.841450	2.545573	0.956615
36	6	0	-2.378828	3.433244	-1.237082
37	1	0	-2.782489	3.091160	-2.196933
38	6	0	-3.089110	4.708469	-0.802931
39	1	0	-2.959654	5.495459	-1.553021
40	1	0	-4.162057	4.541915	-0.664231
41	1	0	-2.681126	5.071052	0.145741
42	6	0	-0.872215	3.663339	-1.406002
43	1	0	-0.365303	2.742672	-1.712443
44	1	0	-0.681347	4.430635	-2.163350
45	1	0	-0.430078	3.999707	-0.461197

```
Final Gibbs Free Energy = Single Point Energy + Thermal correction to Gibbs Free Energy

Single Point Energy calculated under M06-2X/6-311+G**:
SCF Done:  E(RM062X) =  -1374.65134319     A.U.
 SMD-CDS (non-electrostatic) energy       (kcal/mol) =      10.01
 (included in total energy above)

Thermal corrections calculated under M06-2X/6-31G*:
Zero-point correction=                           0.336364 (Hartree/Particle)
 Thermal correction to Energy=                    0.360959
 Thermal correction to Enthalpy=                  0.361903
 Thermal correction to Gibbs Free Energy=         0.281375

Low Frequencies (M06-2X/6-31G*):
Low frequencies --- -257.9158   -9.1612    0.0008    0.0008    0.0009    5.5480
 Low frequencies ---   13.4939   22.0481   35.5220
```

7. M06-2X results for *p*-QM

Center Number	Atomic Number	Atomic Type	Coordinates (Angstroms) X	Y	Z
1	6	0	-3.784179	-1.929159	-0.110279
2	6	0	-2.593270	-1.239607	-0.090630
3	6	0	-2.511147	0.161918	-0.002166
4	6	0	-3.682543	0.907440	0.073628
5	6	0	-4.911381	0.210219	0.054164
6	6	0	-4.965019	-1.169276	-0.034994
7	1	0	-3.805518	-3.011918	-0.179689
8	1	0	-5.822385	0.797851	0.110870
9	1	0	-5.930026	-1.665071	-0.047105
10	6	0	-0.295161	-0.585655	-0.088986
11	6	0	1.148578	-0.549535	-0.077830
12	6	0	1.754089	0.784735	-0.055072
13	6	0	1.001705	1.903090	0.005401
14	6	0	-0.464371	1.858015	0.064338
15	6	0	-1.073053	0.526615	-0.007847
16	1	0	1.460369	2.887151	-0.002864
17	6	0	-1.206188	-1.786589	-0.149725
18	8	0	-0.881776	-2.953632	-0.232184
19	8	0	-3.740244	2.254370	0.164888
20	1	0	-2.821449	2.619345	0.166459
21	8	0	-1.126919	2.898138	0.148221
22	6	0	3.240563	0.925911	-0.213232
23	6	0	1.917401	-1.663302	-0.073165
24	1	0	1.462150	-2.644725	-0.152146
25	6	0	3.381525	-1.588972	0.117780
26	6	0	4.091911	-0.288147	0.046664
27	8	0	3.745344	1.978552	-0.548681
28	8	0	3.784915	-0.889633	1.301997
29	1	0	3.960511	-2.483446	-0.092422
30	6	0	5.540766	-0.193378	-0.327255
31	1	0	5.641914	0.002779	-1.397615
32	1	0	6.018360	0.621830	0.222647
33	1	0	6.044156	-1.132093	-0.086824

```
Final Gibbs Free Energy = Single Point Energy + Thermal correction to Gibbs Free Energy

Single Point Energy calculated under M06-2X/6-311+G**:
SCF Done:  E(RM062X) =  -1067.90256787     A.U
 SMD-CDS (non-electrostatic) energy       (kcal/mol) =       7.08
 (included in total energy above)

Thermal corrections calculated under M06-2X/6-31G*:
Zero-point correction=                           0.240394 (Hartree/Particle)
 Thermal correction to Energy=                    0.257840
 Thermal correction to Enthalpy=                  0.258784
 Thermal correction to Gibbs Free Energy=         0.195135
```

8. M06-2X results for *p*-QM⁻

Center Number	Atomic Number	Atomic Type	X	Y	Z
1	6	0	-3.763821	-1.915642	-0.126922
2	6	0	-2.607890	-1.181322	-0.072518
3	6	0	-2.546921	0.231006	0.008925
4	6	0	-3.754455	0.998230	0.019107
5	6	0	-4.964515	0.182372	-0.033170
6	6	0	-4.979639	-1.186402	-0.101520
7	1	0	-3.742502	-2.999283	-0.187185
8	1	0	-5.899238	0.737954	-0.019335
9	1	0	-5.927895	-1.715522	-0.139902
10	6	0	-0.324670	-0.540299	-0.038706
11	6	0	1.121023	-0.536113	-0.033088
12	6	0	1.752932	0.788009	-0.052138
13	6	0	1.014197	1.910602	0.022742
14	6	0	-0.457958	1.902802	0.158370
15	6	0	-1.117216	0.580965	0.034670
16	1	0	1.483672	2.889716	-0.003671
17	6	0	-1.220760	-1.725631	-0.102975
18	8	0	-0.903031	-2.905258	-0.172315
19	8	0	-3.858679	2.263505	0.062136
20	8	0	-1.050055	2.958147	0.358114
21	6	0	3.232599	0.909061	-0.258868
22	6	0	1.879999	-1.656118	-0.011012
23	1	0	1.415582	-2.635148	-0.042940
24	6	0	3.349733	-1.591355	0.139103
25	6	0	4.078057	-0.306793	0.007734
26	8	0	3.740098	1.945641	-0.643599
27	8	0	3.809608	-0.869340	1.291799
28	1	0	3.913245	-2.497523	-0.065844
29	6	0	5.514843	-0.239318	-0.416670
30	1	0	5.583157	-0.070256	-1.494320
31	1	0	6.021765	0.582367	0.096372
32	1	0	6.015455	-1.178192	-0.170621

(Coordinates in Angstroms)

```
Final Gibbs Free Energy = Single Point Energy + Thermal correction to Gibbs Free Energy

Single Point Energy calculated under M06-2X/6-311+G**:
SCF Done:  E(RM062X) =  -1067.42799151     A.U.
 SMD-CDS (non-electrostatic) energy       (kcal/mol) =       7.46
 (included in total energy above)

Thermal corrections calculated under M06-2X/6-31G*:
Zero-point correction=                           0.226991 (Hartree/Particle)
 Thermal correction to Energy=                    0.244415
 Thermal correction to Enthalpy=                  0.245360
 Thermal correction to Gibbs Free Energy=         0.181589
```

9. M06-2X results for π-CMP-I

Center Number	Atomic Number	Atomic Type	Coordinates (Angstroms) X	Y	Z
1	6	0	1.750928	-1.289668	2.115043
2	6	0	0.420485	-1.179061	1.776399
3	6	0	-0.413535	-2.285043	1.534576
4	6	0	0.120272	-3.565470	1.607688
5	6	0	1.480147	-3.694260	1.971030
6	6	0	2.275018	-2.589563	2.224681
7	1	0	2.367791	-0.409818	2.275803
8	1	0	1.890287	-4.697390	2.038827
9	1	0	3.316420	-2.734797	2.492939
10	6	0	-1.766723	-0.406570	1.202257
11	6	0	-2.936193	0.396814	0.939613
12	6	0	-4.139690	-0.347828	0.569016
13	6	0	-4.144515	-1.699419	0.503264
14	6	0	-2.956994	-2.507876	0.787435
15	6	0	-1.757049	-1.768045	1.171746
16	1	0	-5.036223	-2.236186	0.194331
17	6	0	-0.374439	0.058651	1.538384
18	8	0	0.030833	1.204796	1.553514
19	8	0	-0.568880	-4.700591	1.352290
20	1	0	-1.493169	-4.461324	1.083263
21	8	0	-2.987584	-3.745730	0.701237
22	6	0	-5.367787	0.391015	0.136282
23	6	0	-2.927491	1.751058	0.997263
24	1	0	-2.014846	2.287162	1.230906
25	6	0	-4.167524	2.534929	0.816410
26	6	0	-5.415313	1.881302	0.361805
27	8	0	-6.307064	-0.177160	-0.390347
28	8	0	-5.222259	2.216472	1.737091
29	1	0	-4.061369	3.597486	0.613833
30	6	0	-6.482574	2.634079	-0.375010
31	1	0	-6.377877	2.473884	-1.451789
32	1	0	-7.471804	2.284920	-0.067092
33	1	0	-6.396297	3.701835	-0.162001
34	6	0	-1.977175	2.385600	-1.875469
35	6	0	-0.958507	1.462072	-1.689282
36	6	0	-1.168416	0.082742	-1.765785
37	6	0	-2.447202	-0.461098	-2.047030
38	6	0	-3.469610	0.509247	-2.299306
39	6	0	-3.241420	1.875465	-2.217387
40	1	0	-1.791464	3.452786	-1.803319
41	1	0	-4.456390	0.133976	-2.559939
42	1	0	-4.061338	2.563138	-2.409479
43	6	0	1.122444	0.343802	-1.325054
44	6	0	2.444088	0.000963	-1.087906
45	6	0	2.735303	-1.376798	-1.022067
46	6	0	1.735659	-2.335337	-1.189859
47	6	0	0.406313	-1.977933	-1.445533
48	6	0	0.110969	-0.610781	-1.518145
49	1	0	1.990623	-3.388705	-1.120736
50	6	0	0.483974	1.702321	-1.379683
51	8	0	1.027274	2.778229	-1.193592
52	8	0	-2.689571	-1.733619	-2.069531
53	8	0	-0.522165	-2.939136	-1.574083
54	6	0	4.103130	-1.846255	-0.697544
55	6	0	3.499936	1.061386	-0.929437
56	1	0	3.346905	1.836800	-1.683446
57	6	0	4.911672	0.541544	-1.061950
58	6	0	5.241352	-0.891638	-0.933327
59	8	0	4.324053	-2.964033	-0.257791
60	8	0	5.150932	-0.243533	-2.215125
61	1	0	5.696623	1.257229	-0.820413
62	6	0	6.605812	-1.352792	-0.516225

63	1	0	6.621302	-1.558477	0.557182
64	1	0	6.875891	-2.269369	-1.047364
65	1	0	7.341674	-0.577605	-0.740742
66	1	0	-1.437619	-2.496617	-1.782685
67	8	0	3.369248	1.667305	0.373533
68	6	0	3.798051	2.946097	0.467588
69	8	0	4.238640	3.555435	-0.483951
70	6	0	3.681265	3.493232	1.871402
71	1	0	4.315935	2.850683	2.494915
72	6	0	4.195850	4.926477	1.919363
73	1	0	4.167083	5.292697	2.949016
74	1	0	5.223985	4.998011	1.555318
75	1	0	3.568992	5.580364	1.304622
76	6	0	2.238597	3.384975	2.376362
77	1	0	1.926909	2.345587	2.485942
78	1	0	2.158139	3.874978	3.350843
79	1	0	1.547545	3.881217	1.685675

Final Gibbs Free Energy = Single Point Energy + Thermal correction to Gibbs Free Energy

Single Point Energy calculated under M06-2X/6-311+G**:
SCF Done: E(RM062X) = -2443.12467943   A.U.
 SMD-CDS (non-electrostatic) energy   (kcal/mol) =   12.76
 (included in total energy above)

Thermal corrections calculated under M06-2X/6-31G*:
Zero-point correction=   0.594084 (Hartree/Particle)
 Thermal correction to Energy=   0.636722
 Thermal correction to Enthalpy=   0.637666
 Thermal correction to Gibbs Free Energy=   0.520964

10. M06-2X results for π-CMP-II

Center Number	Atomic Number	Atomic Type	Coordinates (Angstroms) X	Y	Z
1	6	0	1.196243	-3.886246	-0.772679
2	6	0	0.308721	-2.861619	-1.014924
3	6	0	0.603031	-1.762212	-1.840975
4	6	0	1.845514	-1.689044	-2.460118
5	6	0	2.762073	-2.737700	-2.224843
6	6	0	2.451052	-3.806717	-1.401075
7	1	0	0.935953	-4.709919	-0.115049
8	1	0	3.732610	-2.673331	-2.706931
9	1	0	3.187985	-4.587212	-1.242494
10	6	0	-1.586668	-1.407751	-1.099509
11	6	0	-2.881772	-0.793367	-0.946222
12	6	0	-3.033587	0.526258	-1.558303
13	6	0	-2.039129	1.097738	-2.274134
14	6	0	-0.752385	0.433010	-2.492242
15	6	0	-0.587309	-0.875495	-1.854937
16	1	0	-2.155196	2.091978	-2.694614
17	6	0	-1.068241	-2.680185	-0.471234
18	8	0	-1.635170	-3.382926	0.341419
19	8	0	2.238155	-0.685862	-3.275286
20	1	0	1.522257	-0.002557	-3.317765
21	8	0	0.144289	0.978914	-3.151311
22	6	0	-4.279852	1.319877	-1.295595
23	6	0	-3.901383	-1.379246	-0.273443
24	1	0	-3.757071	-2.334114	0.221381
25	6	0	-5.259202	-0.791169	-0.273407
26	6	0	-5.487494	0.582376	-0.778947
27	8	0	-4.321865	2.519538	-1.487359
28	8	0	-5.799438	-0.556397	-1.580898
29	1	0	-5.974741	-1.182603	0.444267
30	6	0	-6.639484	1.422687	-0.315495
31	1	0	-6.316700	2.095043	0.483733
32	1	0	-7.019597	2.026778	-1.143839
33	1	0	-7.439618	0.780353	0.058307
34	6	0	-2.729976	1.528299	1.449196
35	6	0	-1.525385	0.846687	1.417724
36	6	0	-1.337887	-0.405544	2.018983
37	6	0	-2.399457	-1.089022	2.653350
38	6	0	-3.636705	-0.366757	2.680902
39	6	0	-3.789916	0.894267	2.120091
40	1	0	-2.843588	2.502501	0.983231
41	1	0	-4.477539	-0.846937	3.175317
42	1	0	-4.756233	1.387878	2.188696
43	6	0	0.731227	0.152484	1.053828
44	6	0	2.070711	0.044919	0.705817
45	6	0	2.755246	-1.095422	1.166300
46	6	0	2.086240	-2.086861	1.891721
47	6	0	0.727353	-1.987533	2.207861
48	6	0	0.058148	-0.824668	1.805009
49	1	0	2.634303	-2.967261	2.213541
50	6	0	-0.259835	1.227343	0.724031
51	8	0	-0.095805	2.183711	-0.018639
52	8	0	-2.287409	-2.274834	3.170932
53	8	0	0.119492	-2.990480	2.864049
54	6	0	4.214450	-1.257378	0.958805
55	6	0	2.735186	1.143905	-0.080222
56	1	0	2.165626	1.336418	-0.996662
57	6	0	4.172615	0.877071	-0.457368
58	6	0	4.929177	-0.279255	0.059568
59	8	0	4.852832	-2.143664	1.506153
60	8	0	4.376105	-0.265898	-1.267310
61	1	0	4.739102	1.759177	-0.754643
62	6	0	6.426631	-0.268915	0.144669

63	1	0	6.746562	-0.001417	1.155170
64	1	0	6.825200	-1.258496	-0.093310
65	1	0	6.831236	0.459969	-0.560979
66	1	0	-0.882836	-2.741394	3.009820
67	8	0	2.702811	2.323156	0.742386
68	6	0	2.522146	3.504570	0.116699
69	8	0	2.495735	3.612090	-1.090466
70	6	0	2.373767	4.634611	1.109236
71	1	0	3.265110	4.592981	1.747417
72	6	0	2.312920	5.973361	0.386594
73	1	0	2.250731	6.784824	1.116799
74	1	0	3.197588	6.134297	-0.235383
75	1	0	1.428826	6.021823	-0.256872
76	6	0	1.138719	4.407567	1.990881
77	1	0	1.183785	3.445141	2.507791
78	1	0	1.080169	5.200517	2.741645
79	1	0	0.225219	4.437237	1.388506

Final Gibbs Free Energy = Single Point Energy + Thermal correction to Gibbs Free Energy

Single Point Energy calculated under M06-2X/6-311+G**:
SCF Done: E(RM062X) = -2443.12203571 A.U.
SMD-CDS (non-electrostatic) energy (kcal/mol) = 13.08
(included in total energy above)

Thermal corrections calculated under M06-2X/6-31G*:
Zero-point correction= 0.594177 (Hartree/Particle)
Thermal correction to Energy= 0.636784
Thermal correction to Enthalpy= 0.637728
Thermal correction to Gibbs Free Energy= 0.521476

11. M06-2X results for π-CMP-III

Center Number	Atomic Number	Atomic Type	Coordinates (Angstroms) X	Y	Z
1	6	0	-6.907944	-1.432974	-0.552674
2	6	0	-5.749714	-0.850931	-0.087270
3	6	0	-5.406310	0.490434	-0.329827
4	6	0	-6.267149	1.288738	-1.075849
5	6	0	-7.456789	0.702781	-1.560292
6	6	0	-7.772228	-0.621947	-1.307533
7	1	0	-7.136298	-2.473317	-0.344349
8	1	0	-8.123937	1.328547	-2.145143
9	1	0	-8.697000	-1.032061	-1.699682
10	6	0	-3.642922	-0.380416	0.935150
11	6	0	-2.367895	-0.465002	1.607494
12	6	0	-1.572885	0.766400	1.605914
13	6	0	-2.007888	1.892448	0.996250
14	6	0	-3.296000	1.965637	0.301418
15	6	0	-4.100261	0.747382	0.323625
16	1	0	-1.391607	2.786248	0.980493
17	6	0	-4.662853	-1.468341	0.731789
18	8	0	-4.623962	-2.610862	1.145430
19	8	0	-6.047233	2.590444	-1.370121
20	1	0	-5.173920	2.864431	-0.985379
21	8	0	-3.661882	3.018190	-0.245978
22	6	0	-0.195149	0.774793	2.201163
23	6	0	-1.888989	-1.610624	2.149286
24	1	0	-2.461720	-2.529291	2.088530
25	6	0	-0.632563	-1.624814	2.929935
26	6	0	0.241906	-0.428775	2.996838
27	8	0	0.568118	1.706473	2.030944
28	8	0	-0.655214	-0.741068	4.061832
29	1	0	-0.169238	-2.588753	3.123405
30	6	0	1.702081	-0.519197	3.328437
31	1	0	2.306237	-0.467802	2.420344
32	1	0	1.987020	0.314176	3.976587
33	1	0	1.907442	-1.460996	3.841818
34	6	0	-0.691234	-2.913100	-0.476262
35	6	0	0.061569	-1.752381	-0.554606
36	6	0	-0.452454	-0.546233	-1.044149
37	6	0	-1.781266	-0.449656	-1.523606
38	6	0	-2.519736	-1.677270	-1.499138
39	6	0	-2.000915	-2.854770	-0.984924
40	1	0	-0.273627	-3.831542	-0.075594
41	1	0	-3.533533	-1.650948	-1.893726
42	1	0	-2.623220	-3.745152	-0.969169
43	6	0	1.766740	-0.086029	-0.443033
44	6	0	2.933301	0.635177	-0.239612
45	6	0	2.896945	2.002790	-0.567698
46	6	0	1.726617	2.592721	-1.053246
47	6	0	0.553917	1.856324	-1.254526
48	6	0	0.593325	0.488747	-0.954649
49	1	0	1.726101	3.651999	-1.292107
50	6	0	1.480797	-1.529492	-0.150714
51	8	0	2.245350	-2.348146	0.334671
52	8	0	-2.315877	0.657241	-1.929887
53	8	0	-0.545422	2.477887	-1.720406
54	6	0	4.113636	2.843608	-0.467983
55	6	0	4.180500	-0.063211	0.231643
56	1	0	3.950490	-0.709750	1.082681
57	6	0	5.293341	0.866945	0.641418
58	6	0	5.284459	2.305738	0.312409
59	8	0	4.194344	3.940033	-0.999468
60	8	0	4.907726	1.821915	1.614441
61	1	0	6.260449	0.393607	0.808772
62	6	0	6.553299	3.102971	0.248608

63	1	0	7.322999	2.618695	0.853528
64	1	0	6.904660	3.172837	-0.783962
65	1	0	6.384432	4.115197	0.625292
66	1	0	-1.299192	1.782850	-1.831552
67	8	0	4.623998	-0.898748	-0.852017
68	6	0	5.187354	-2.079037	-0.521445
69	8	0	5.454608	-2.381499	0.622318
70	6	0	5.403853	-2.950148	-1.734955
71	1	0	6.000027	-3.798200	-1.387211
72	6	0	4.044624	-3.454697	-2.236289
73	1	0	4.197607	-4.116464	-3.093667
74	1	0	3.513794	-4.012129	-1.459137
75	1	0	3.416415	-2.616342	-2.555018
76	6	0	6.157714	-2.198294	-2.833401
77	1	0	7.110768	-1.803515	-2.469321
78	1	0	6.362490	-2.879558	-3.664013
79	1	0	5.558928	-1.365414	-3.212689

Final Gibbs Free Energy = Single Point Energy + Thermal correction to Gibbs Free Energy

Single Point Energy calculated under M06-2X/6-311+G**:
SCF Done: E(RM062X) = -2443.11780844   A.U.
SMD-CDS (non-electrostatic) energy   (kcal/mol) =   13.77
(included in total energy above)

Thermal corrections calculated under M06-2X/6-31G*:
Zero-point correction=   0.594755 (Hartree/Particle)
Thermal correction to Energy=   0.637577
Thermal correction to Enthalpy=   0.638522
Thermal correction to Gibbs Free Energy=   0.519922

12. M06-2X results for π-CMP-IV

Center Number	Atomic Number	Atomic Type	Coordinates (Angstroms) X	Y	Z
1	6	0	4.432121	3.448932	0.848944
2	6	0	3.693379	2.443826	0.264998
3	6	0	2.620603	2.683243	-0.612019
4	6	0	2.309308	3.992499	-0.961700
5	6	0	3.043767	5.032674	-0.351443
6	6	0	4.075990	4.771484	0.534045
7	1	0	5.256819	3.223320	1.517280
8	1	0	2.783175	6.053898	-0.612592
9	1	0	4.619402	5.599495	0.977102
10	6	0	2.818915	0.354616	-0.500084
11	6	0	2.569947	-1.037414	-0.777652
12	6	0	1.362041	-1.323778	-1.548398
13	6	0	0.573457	-0.336371	-2.029260
14	6	0	0.915036	1.079619	-1.868594
15	6	0	2.078212	1.366871	-1.031672
16	1	0	-0.332263	-0.566004	-2.582877
17	6	0	3.895906	0.968157	0.362755
18	8	0	4.755749	0.387776	0.994161
19	8	0	1.367052	4.343914	-1.865291
20	1	0	0.893802	3.530359	-2.173951
21	8	0	0.238447	1.964500	-2.414886
22	6	0	0.941277	-2.747397	-1.772120
23	6	0	3.398369	-2.035354	-0.381589
24	1	0	4.277101	-1.814429	0.214031
25	6	0	3.207922	-3.427063	-0.851806
26	6	0	1.976020	-3.823898	-1.576801
27	8	0	-0.192462	-3.027575	-2.108896
28	8	0	3.185545	-3.540830	-2.280759
29	1	0	3.761029	-4.211558	-0.342259
30	6	0	1.497112	-5.243983	-1.647269
31	1	0	0.670495	-5.402181	-0.953089
32	1	0	1.150486	-5.461776	-2.661519
33	1	0	2.315903	-5.921700	-1.396259
34	6	0	2.009049	-0.554506	2.575687
35	6	0	0.793696	-0.856136	1.991305
36	6	0	0.431225	-2.152096	1.595801
37	6	0	1.296894	-3.251531	1.786194
38	6	0	2.552200	-2.920475	2.392399
39	6	0	2.891598	-1.629210	2.773965
40	1	0	2.261700	0.461470	2.865412
41	1	0	3.253395	-3.735477	2.555599
42	1	0	3.864012	-1.451115	3.225351
43	6	0	-1.417358	-0.789633	1.092350
44	6	0	-2.691400	-0.442282	0.661728
45	6	0	-3.456771	-1.462204	0.068050
46	6	0	-2.963434	-2.768055	-0.031504
47	6	0	-1.708149	-3.123442	0.469099
48	6	0	-0.924146	-2.101744	1.021617
49	1	0	-3.577249	-3.533302	-0.497158
50	6	0	-0.336248	0.068506	1.679321
51	8	0	-0.357019	1.275438	1.861238
52	8	0	1.006276	-4.471365	1.441964
53	8	0	-1.293153	-4.399359	0.391117
54	6	0	-4.784577	-1.172796	-0.522578
55	6	0	-3.214410	0.950853	0.884681
56	1	0	-3.048874	1.221702	1.932287
57	6	0	-4.684771	1.122037	0.593412
58	6	0	-5.480291	0.098632	-0.111864
59	8	0	-5.321834	-1.928000	-1.318310
60	8	0	-5.530123	0.246969	1.318949
61	1	0	-5.027519	2.154723	0.535207
62	6	0	-6.732614	0.451852	-0.858173

63	1	0	-6.511873	0.591651	-1.919639
64	1	0	-7.471871	-0.347547	-0.761247
65	1	0	-7.150852	1.378229	-0.458285
66	1	0	-0.349203	-4.479218	0.827234
67	8	0	-2.465543	1.864482	0.069109
68	6	0	-2.199772	3.065105	0.632101
69	8	0	-2.706175	3.428127	1.672355
70	6	0	-1.232754	3.862932	-0.202028
71	1	0	-0.379088	3.197329	-0.374360
72	6	0	-1.883182	4.194174	-1.551252
73	1	0	-1.180934	4.763713	-2.166514
74	1	0	-2.165212	3.289401	-2.095241
75	1	0	-2.779749	4.805130	-1.398408
76	6	0	-0.779829	5.111821	0.540780
77	1	0	-0.276328	4.856181	1.478079
78	1	0	-0.082825	5.678159	-0.083261
79	1	0	-1.633574	5.756154	0.774491

Final Gibbs Free Energy = Single Point Energy + Thermal correction to Gibbs Free Energy

Single Point Energy calculated under M06-2X/6-311+G**:
SCF Done: E(RM062X) = -2443.11513828   A.U.
 SMD-CDS (non-electrostatic) energy   (kcal/mol) =   12.94
 (included in total energy above)

Thermal corrections calculated under M06-2X/6-31G*:
Zero-point correction=   0.595424 (Hartree/Particle)
 Thermal correction to Energy=   0.635625
 Thermal correction to Enthalpy=   0.636569
 Thermal correction to Gibbs Free Energy=   0.526102

13. M06-2X results for π-CMP-V

Center Number	Atomic Number	Atomic Type	Coordinates (Angstroms) X	Y	Z
1	6	0	-0.976966	2.574874	-1.807263
2	6	0	0.196827	2.120920	-1.250961
3	6	0	0.776276	2.681095	-0.099608
4	6	0	0.132206	3.731968	0.540420
5	6	0	-1.065647	4.220999	-0.030130
6	6	0	-1.609466	3.659607	-1.172778
7	1	0	-1.396334	2.107429	-2.691305
8	1	0	-1.561457	5.046990	0.470657
9	1	0	-2.538485	4.055099	-1.572993
10	6	0	2.229540	0.968362	-0.754207
11	6	0	3.433901	0.170811	-0.836270
12	6	0	4.442108	0.442967	0.191753
13	6	0	4.210057	1.320792	1.192894
14	6	0	2.970326	2.101831	1.288352
15	6	0	2.021636	1.927636	0.188402
16	1	0	4.969318	1.518237	1.943607
17	6	0	1.040684	0.968438	-1.681951
18	8	0	0.790235	0.161591	-2.556500
19	8	0	0.561084	4.314703	1.683568
20	1	0	1.372543	3.842504	2.000452
21	8	0	2.788303	2.903031	2.214640
22	6	0	5.811853	-0.155487	0.075433
23	6	0	3.663858	-0.718632	-1.830976
24	1	0	2.923325	-0.873141	-2.608518
25	6	0	4.927987	-1.485982	-1.913291
26	6	0	6.054198	-1.190425	-0.996198
27	8	0	6.723497	0.194042	0.800789
28	8	0	5.229222	-2.336316	-0.800494
29	1	0	5.174132	-1.922708	-2.877365
30	6	0	7.483664	-1.448149	-1.370112
31	1	0	7.948024	-0.533368	-1.747325
32	1	0	8.043906	-1.786598	-0.494520
33	1	0	7.527833	-2.217517	-2.143902
34	6	0	1.627544	-3.215645	-0.307075
35	6	0	0.660174	-2.244796	-0.107519
36	6	0	0.767438	-1.259942	0.880027
37	6	0	1.896468	-1.182210	1.730004
38	6	0	2.861299	-2.215565	1.538122
39	6	0	2.725803	-3.197573	0.566079
40	1	0	1.517639	-3.972904	-1.076790
41	1	0	3.733563	-2.209463	2.187132
42	1	0	3.498449	-3.953932	0.470564
43	6	0	-1.326970	-0.913914	-0.138423
44	6	0	-2.560304	-0.331222	-0.380045
45	6	0	-2.873906	0.826955	0.363531
46	6	0	-2.013098	1.316064	1.342252
47	6	0	-0.787909	0.693331	1.617811
48	6	0	-0.446539	-0.422672	0.845473
49	1	0	-2.276714	2.216741	1.888816
50	6	0	-0.629461	-2.054068	-0.833040
51	8	0	-1.002816	-2.667678	-1.817406
52	8	0	2.061312	-0.243500	2.617613
53	8	0	0.022285	1.218390	2.549716
54	6	0	-4.089620	1.611978	0.040262
55	6	0	-3.627555	-0.944807	-1.252111
56	1	0	-3.215203	-1.688036	-1.934834
57	6	0	-4.445490	0.097672	-1.978054
58	6	0	-4.726077	1.385107	-1.311397
59	8	0	-4.557477	2.448007	0.797369
60	8	0	-3.765670	1.270257	-2.376200
61	1	0	-5.185245	-0.280255	-2.680970
62	6	0	-5.958790	2.182691	-1.616388

63	1	0	-6.740221	1.965523	-0.883903
64	1	0	-5.737664	3.253037	-1.579334
65	1	0	-6.324578	1.926369	-2.613006
66	1	0	0.890511	0.634824	2.619451
67	8	0	-4.620268	-1.566315	-0.398841
68	6	0	-4.310155	-2.734816	0.193113
69	8	0	-3.311578	-3.366744	-0.074961
70	6	0	-5.327610	-3.096093	1.249028
71	1	0	-6.318515	-2.972552	0.798279
72	6	0	-5.131162	-4.530433	1.719948
73	1	0	-5.896174	-4.785281	2.458378
74	1	0	-5.201779	-5.238212	0.889297
75	1	0	-4.149240	-4.649419	2.188302
76	6	0	-5.185760	-2.091255	2.402756
77	1	0	-5.369259	-1.066760	2.067545
78	1	0	-5.903608	-2.332683	3.191473
79	1	0	-4.177973	-2.142141	2.829293

```
---------------------------------------------------------------------
Final Gibbs Free Energy = Single Point Energy + Thermal correction to Gibbs Free Energy

Single Point Energy calculated under M06-2X/6-311+G**:
SCF Done:  E(RM062X) =  -2443.11328995     A.U.
 SMD-CDS (non-electrostatic) energy       (kcal/mol) =      14.07
 (included in total energy above)

Thermal corrections calculated under M06-2X/6-31G*:
Zero-point correction=                           0.593956 (Hartree/Particle)
 Thermal correction to Energy=                    0.637025
 Thermal correction to Enthalpy=                  0.637969
 Thermal correction to Gibbs Free Energy=         0.518911
```

14. M06-2X results for π-CMP-VI

Center Number	Atomic Number	Atomic Type	Coordinates (Angstroms) X	Y	Z
1	6	0	-1.507656	-0.380045	-2.581300
2	6	0	-0.306417	0.090061	-2.099005
3	6	0	-0.079835	1.443784	-1.786912
4	6	0	-1.104683	2.366172	-1.967303
5	6	0	-2.354091	1.886683	-2.421642
6	6	0	-2.553121	0.550782	-2.723498
7	1	0	-1.643521	-1.426057	-2.836006
8	1	0	-3.155524	2.609231	-2.543271
9	1	0	-3.524014	0.222351	-3.082337
10	6	0	1.937201	0.354359	-1.314987
11	6	0	3.309995	0.159898	-0.913570
12	6	0	4.067540	1.378507	-0.627385
13	6	0	3.483559	2.596029	-0.638245
14	6	0	2.060707	2.777257	-0.942196
15	6	0	1.324803	1.569669	-1.323413
16	1	0	4.061226	3.494212	-0.442178
17	6	0	0.955977	-0.666098	-1.837906
18	8	0	1.166749	-1.843775	-2.045941
19	8	0	-0.995910	3.694295	-1.744080
20	1	0	-0.081753	3.895983	-1.420548
21	8	0	1.545901	3.902934	-0.913606
22	6	0	5.554489	1.297524	-0.433454
23	6	0	3.880291	-1.062545	-0.781108
24	1	0	3.331846	-1.962814	-1.043000
25	6	0	5.250449	-1.218783	-0.242656
26	6	0	6.143495	-0.042292	-0.083886
27	8	0	6.264207	2.275257	-0.560072
28	8	0	5.473946	-0.616933	1.035431
29	1	0	5.724015	-2.188369	-0.367752
30	6	0	7.636830	-0.163020	-0.122969
31	1	0	8.006312	0.058627	-1.127307
32	1	0	8.088529	0.543344	0.578538
33	1	0	7.930468	-1.178872	0.149509
34	6	0	1.858321	-0.781626	2.986772
35	6	0	0.778396	-1.151274	2.207773
36	6	0	0.834459	-2.195042	1.271270
37	6	0	2.019781	-2.926314	1.046613
38	6	0	3.121373	-2.539519	1.872104
39	6	0	3.043370	-1.513493	2.803150
40	1	0	1.788241	0.028947	3.705043
41	1	0	4.055328	-3.081075	1.738984
42	1	0	3.923782	-1.267238	3.389602
43	6	0	-1.348781	-1.341740	1.141485
44	6	0	-2.652497	-1.181552	0.692980
45	6	0	-3.084142	-2.058676	-0.317115
46	6	0	-2.246457	-3.070102	-0.799459
47	6	0	-0.939689	-3.233482	-0.333059
48	6	0	-0.486083	-2.323290	0.633139
49	1	0	-2.608017	-3.728200	-1.584169
50	6	0	-0.595079	-0.567206	2.180476
51	8	0	-1.017964	0.342785	2.875317
52	8	0	2.133801	-3.861365	0.146077
53	8	0	-0.169897	-4.217910	-0.825492
54	6	0	-4.372434	-1.833251	-1.010221
55	6	0	-3.514215	-0.081287	1.246913
56	1	0	-3.499515	-0.122770	2.339800
57	6	0	-4.952851	-0.129781	0.794863
58	6	0	-5.404452	-0.979360	-0.325293
59	8	0	-4.600901	-2.293864	-2.119525
60	8	0	-5.605134	-1.361027	1.047518
61	1	0	-5.540291	0.759916	1.019448
62	6	0	-6.637965	-0.649663	-1.112284

63	1	0	-6.372020	-0.082553	-2.008469
64	1	0	-7.148736	-1.565422	-1.421190
65	1	0	-7.314139	-0.048569	-0.500454
66	1	0	0.798816	-4.111037	-0.449429
67	8	0	-2.943652	1.174969	0.838061
68	6	0	-3.063244	2.196969	1.710581
69	8	0	-3.699586	2.110510	2.739578
70	6	0	-2.311670	3.418754	1.243826
71	1	0	-2.543996	3.542765	0.180085
72	6	0	-2.751100	4.648999	2.027573
73	1	0	-2.222709	5.531465	1.656444
74	1	0	-3.826193	4.824038	1.929686
75	1	0	-2.518772	4.531586	3.090583
76	6	0	-0.802233	3.172993	1.380896
77	1	0	-0.482480	2.290327	0.817292
78	1	0	-0.259336	4.043878	1.003251
79	1	0	-0.534081	3.025315	2.432438

Final Gibbs Free Energy = Single Point Energy + Thermal correction to Gibbs Free Energy

Single Point Energy calculated under M06-2X/6-311+G**:
SCF Done: E(RM062X) = -2443.11426664 A.U.
SMD-CDS (non-electrostatic) energy (kcal/mol) = 13.12
(included in total energy above)

Thermal corrections calculated under M06-2X/6-31G*:
Zero-point correction= 0.594903 (Hartree/Particle)
Thermal correction to Energy= 0.637435
Thermal correction to Enthalpy= 0.638379
Thermal correction to Gibbs Free Energy= 0.521427

15. M06-2X results for π-CMP-VII

Center Number	Atomic Number	Atomic Type	Coordinates (Angstroms) X	Y	Z
1	6	0	-0.624411	0.765002	3.028128
2	6	0	0.465636	0.256765	2.355554
3	6	0	0.511227	-1.044975	1.824668
4	6	0	-0.587623	-1.883584	1.988184
5	6	0	-1.698213	-1.383202	2.702519
6	6	0	-1.725566	-0.089559	3.199283
7	1	0	-0.624394	1.783408	3.404831
8	1	0	-2.547677	-2.044353	2.837511
9	1	0	-2.607451	0.258262	3.727940
10	6	0	2.581826	-0.085933	1.308169
11	6	0	3.902780	0.066743	0.749430
12	6	0	4.419052	-1.081785	0.002550
13	6	0	3.685528	-2.202759	-0.166663
14	6	0	2.327308	-2.340783	0.376706
15	6	0	1.826678	-1.208217	1.158680
16	1	0	4.083472	-3.056572	-0.707025
17	6	0	1.768501	0.925920	2.077387
18	8	0	2.109706	2.037686	2.431425
19	8	0	-0.668722	-3.150524	1.521521
20	1	0	0.141992	-3.349484	0.990926
21	8	0	1.665522	-3.367255	0.180378
22	6	0	5.830972	-1.059501	-0.503960
23	6	0	4.621379	1.207631	0.866772
24	1	0	4.254024	2.030235	1.470899
25	6	0	5.875631	1.406759	0.109475
26	6	0	6.521123	0.275719	-0.602042
27	8	0	6.412874	-2.080176	-0.815106
28	8	0	5.735634	1.234010	-1.306979
29	1	0	6.508902	2.241492	0.395304
30	6	0	7.991864	0.256674	-0.894735
31	1	0	8.527496	-0.275901	-0.105050
32	1	0	8.178953	-0.248793	-1.845882
33	1	0	8.366367	1.280800	-0.953133
34	6	0	0.351839	3.669847	0.251266
35	6	0	-0.126988	2.435970	-0.139073
36	6	0	0.656618	1.505281	-0.842434
37	6	0	1.983072	1.792222	-1.235138
38	6	0	2.465651	3.069343	-0.809963
39	6	0	1.683820	3.967126	-0.098107
40	1	0	-0.265805	4.370684	0.803877
41	1	0	3.489031	3.324867	-1.075381
42	1	0	2.112147	4.921970	0.193994
43	6	0	-1.419982	0.463973	-0.512833
44	6	0	-2.383820	-0.534211	-0.525979
45	6	0	-2.023755	-1.755821	-1.123946
46	6	0	-0.740336	-1.951123	-1.644454
47	6	0	0.225902	-0.939441	-1.630140
48	6	0	-0.138958	0.288770	-1.057867
49	1	0	-0.485458	-2.912497	-2.080645
50	6	0	-1.475836	1.835381	0.089512
51	8	0	-2.424165	2.356644	0.655379
52	8	0	2.721998	0.971866	-1.921183
53	8	0	1.446250	-1.175139	-2.135538
54	6	0	-2.999235	-2.863092	-1.257480
55	6	0	-3.752208	-0.269266	0.039557
56	1	0	-3.658547	0.193847	1.026381
57	6	0	-4.620863	-1.495363	0.170022
58	6	0	-4.283553	-2.779782	-0.473801
59	8	0	-2.794643	-3.827181	-1.979164
60	8	0	-4.043948	-2.546232	0.925308
61	1	0	-5.672039	-1.298932	0.379196
62	6	0	-5.336581	-3.797046	-0.798331

```
     63          1           0       -5.644146   -3.703535   -1.843107
     64          1           0       -4.948097   -4.806561   -0.641331
     65          1           0       -6.206601   -3.642825   -0.156289
     66          1           0        2.020949   -0.295566   -2.068471
     67          8           0       -4.397372    0.669295   -0.840197
     68          6           0       -5.209307    1.582350   -0.267651
     69          8           0       -5.519035    1.543917    0.904128
     70          6           0       -5.647643    2.630308   -1.263531
     71          1           0       -6.041121    2.087781   -2.131437
     72          6           0       -6.729684    3.515750   -0.661092
     73          1           0       -7.069246    4.244571   -1.401930
     74          1           0       -7.592026    2.927811   -0.334643
     75          1           0       -6.339262    4.060226    0.204380
     76          6           0       -4.428723    3.447361   -1.715517
     77          1           0       -3.657976    2.809909   -2.157561
     78          1           0       -4.742704    4.180816   -2.463510
     79          1           0       -3.992296    3.986338   -0.868381
---------------------------------------------------------------------
Final Gibbs Free Energy = Single Point Energy + Thermal correction to Gibbs Free Energy

Single Point Energy calculated under M06-2X/6-311+G**:
SCF Done:  E(RM062X) =  -2443.12177664     A.U.
 SMD-CDS (non-electrostatic) energy       (kcal/mol) =      13.46
 (included in total energy above)

Thermal corrections calculated under M06-2X/6-31G*:
Zero-point correction=                           0.593493 (Hartree/Particle)
 Thermal correction to Energy=                    0.636079
 Thermal correction to Enthalpy=                  0.637023
 Thermal correction to Gibbs Free Energy=         0.520886
```

16. M06-2X results for π-CMP-VIII

Center Number	Atomic Number	Atomic Type	Coordinates (Angstroms) X	Y	Z
1	6	0	-0.062233	-3.380044	2.423715
2	6	0	-0.689161	-2.223777	2.015203
3	6	0	-0.035165	-0.982335	1.907351
4	6	0	1.317473	-0.902303	2.217101
5	6	0	1.968825	-2.079767	2.647646
6	6	0	1.301652	-3.288526	2.749630
7	1	0	-0.603547	-4.319387	2.479927
8	1	0	3.029724	-2.009902	2.871681
9	1	0	1.844678	-4.171641	3.070189
10	6	0	-2.251794	-0.581136	1.270790
11	6	0	-3.416534	0.121796	0.790649
12	6	0	-3.229649	1.540750	0.486426
13	6	0	-2.023565	2.135919	0.605103
14	6	0	-0.834630	1.407646	1.061343
15	6	0	-1.031116	0.004524	1.421470
16	1	0	-1.894962	3.192221	0.386915
17	6	0	-2.115992	-2.041831	1.630861
18	8	0	-2.983985	-2.892136	1.617704
19	8	0	2.068952	0.217648	2.136560
20	1	0	1.536777	0.939611	1.722632
21	8	0	0.263648	1.972668	1.136631
22	6	0	-4.419048	2.389198	0.141833
23	6	0	-4.613167	-0.481148	0.593354
24	1	0	-4.755420	-1.521630	0.866022
25	6	0	-5.738235	0.225389	-0.056176
26	6	0	-5.681523	1.690367	-0.288314
27	8	0	-4.377898	3.600928	0.218596
28	8	0	-5.437223	0.768852	-1.347783
29	1	0	-6.722046	-0.228541	0.017247
30	6	0	-6.920181	2.528710	-0.386592
31	1	0	-7.148384	2.976671	0.583817
32	1	0	-6.774845	3.330887	-1.114709
33	1	0	-7.761550	1.907411	-0.700074
34	6	0	-2.526985	-0.496068	-2.339565
35	6	0	-1.237162	-0.736259	-1.914570
36	6	0	-0.810500	-1.984984	-1.429375
37	6	0	-1.700808	-3.069299	-1.292829
38	6	0	-3.032887	-2.804941	-1.748327
39	6	0	-3.425040	-1.578880	-2.259397
40	1	0	-2.835384	0.480422	-2.700211
41	1	0	-3.752464	-3.615807	-1.668308
42	1	0	-4.453778	-1.446500	-2.583690
43	6	0	1.063639	-0.569745	-1.283604
44	6	0	2.383461	-0.190063	-1.066395
45	6	0	3.254452	-1.194531	-0.602472
46	6	0	2.816097	-2.501895	-0.385699
47	6	0	1.495516	-2.885432	-0.644085
48	6	0	0.615524	-1.889484	-1.084385
49	1	0	3.519983	-3.248057	-0.028298
50	6	0	-0.101596	0.225446	-1.795677
51	8	0	-0.137282	1.423823	-2.028002
52	8	0	-1.369776	-4.217220	-0.773393
53	8	0	1.129761	-4.168179	-0.482500
54	6	0	4.676851	-0.882727	-0.332414
55	6	0	2.888756	1.208400	-1.315271
56	1	0	2.299833	1.691523	-2.094815
57	6	0	4.359415	1.248208	-1.666666
58	6	0	5.289954	0.233849	-1.133672
59	8	0	5.346460	-1.507181	0.475745
60	8	0	4.850129	0.222694	-2.505446
61	1	0	4.758798	2.243912	-1.858022
62	6	0	6.740229	0.528761	-0.894921

63	1	0	6.901218	0.798411	0.151911
64	1	0	7.349396	-0.350436	-1.122023
65	1	0	7.058210	1.359650	-1.528447
66	1	0	0.089221	-4.239454	-0.590482
67	8	0	2.803156	1.995990	-0.105000
68	6	0	2.306373	3.245152	-0.200124
69	8	0	1.769930	3.667238	-1.203067
70	6	0	2.584055	4.098560	1.022580
71	1	0	3.504925	4.630408	0.738258
72	6	0	1.472011	5.130093	1.202141
73	1	0	1.723006	5.802058	2.027393
74	1	0	1.332580	5.727796	0.298859
75	1	0	0.526081	4.632283	1.439423
76	6	0	2.864969	3.331940	2.312025
77	1	0	3.632986	2.566926	2.177490
78	1	0	3.217334	4.036220	3.071205
79	1	0	1.956979	2.856751	2.693588

Final Gibbs Free Energy = Single Point Energy + Thermal correction to Gibbs Free Energy

Single Point Energy calculated under M06-2X/6-311+G**:
SCF Done: E(RM062X) = -2443.11556297 A.U.
SMD-CDS (non-electrostatic) energy (kcal/mol) = 12.91
(included in total energy above)

Thermal corrections calculated under M06-2X/6-31G*:
Zero-point correction= 0.593139 (Hartree/Particle)
Thermal correction to Energy= 0.636049
Thermal correction to Enthalpy= 0.636993
Thermal correction to Gibbs Free Energy= 0.519746

17. M06-2X results for TScp-I

Center Number	Atomic Number	Atomic Type	Coordinates (Angstroms) X	Y	Z
1	6	0	1.904396	-1.282378	2.010088
2	6	0	0.562074	-1.174887	1.706022
3	6	0	-0.290654	-2.282235	1.566235
4	6	0	0.229993	-3.565477	1.705122
5	6	0	1.601112	-3.688647	2.015129
6	6	0	2.418744	-2.577955	2.171935
7	1	0	2.535341	-0.402010	2.093955
8	1	0	2.005918	-4.690139	2.126886
9	1	0	3.468938	-2.720880	2.405758
10	6	0	-1.616412	-0.417617	1.067252
11	6	0	-2.751345	0.362586	0.724442
12	6	0	-3.974495	-0.378448	0.570726
13	6	0	-4.020192	-1.743012	0.715752
14	6	0	-2.852553	-2.540562	0.999673
15	6	0	-1.643050	-1.790991	1.203841
16	1	0	-4.962624	-2.266106	0.583974
17	6	0	-0.222689	0.050762	1.392006
18	8	0	0.187290	1.198167	1.384334
19	8	0	-0.497894	-4.694910	1.554171
20	1	0	-1.451291	-4.434792	1.357175
21	8	0	-2.911992	-3.804411	1.089352
22	6	0	-5.233193	0.336637	0.201564
23	6	0	-2.684606	1.740834	0.449982
24	1	0	-1.784808	2.278903	0.729087
25	6	0	-3.950922	2.522200	0.460251
26	6	0	-5.255571	1.840964	0.329410
27	8	0	-6.223492	-0.252402	-0.196619
28	8	0	-4.782368	2.299188	1.601488
29	1	0	-3.897319	3.559595	0.137907
30	6	0	-6.458214	2.540402	-0.231115
31	1	0	-6.554811	2.325666	-1.299099
32	1	0	-7.366125	2.198064	0.272605
33	1	0	-6.355592	3.618781	-0.091630
34	6	0	-2.205905	1.993240	-1.697488
35	6	0	-1.048951	1.180359	-1.724498
36	6	0	-1.118661	-0.187222	-1.833054
37	6	0	-2.386113	-0.845251	-2.048856
38	6	0	-3.497175	0.037567	-2.349803
39	6	0	-3.399785	1.393454	-2.212335
40	1	0	-2.102980	3.074334	-1.670796
41	1	0	-4.429367	-0.432689	-2.650107
42	1	0	-4.265845	2.021639	-2.404780
43	6	0	1.145132	0.278678	-1.460006
44	6	0	2.486593	0.069342	-1.199236
45	6	0	2.911524	-1.271499	-1.104827
46	6	0	2.013235	-2.322328	-1.269401
47	6	0	0.654588	-2.096962	-1.531537
48	6	0	0.229783	-0.770603	-1.638423
49	1	0	2.360872	-3.345032	-1.158382
50	6	0	0.374525	1.569191	-1.482783
51	8	0	0.805802	2.696428	-1.329390
52	8	0	-2.530352	-2.097986	-1.978943
53	8	0	-0.167890	-3.159457	-1.626323
54	6	0	4.302880	-1.596794	-0.702955
55	6	0	3.397678	1.235694	-0.928629
56	1	0	3.228698	2.020210	-1.670717
57	6	0	4.863562	0.882473	-0.928178
58	6	0	5.339101	-0.510433	-0.809487
59	8	0	4.617211	-2.699466	-0.285351
60	8	0	5.291718	0.171572	-2.075405
61	1	0	5.534310	1.672285	-0.591426
62	6	0	6.704808	-0.830629	-0.279588

63	1	0	6.651766	-1.056399	0.788540
64	1	0	7.117647	-1.700521	-0.797183
65	1	0	7.367253	0.024383	-0.430486
66	1	0	-1.112170	-2.832717	-1.751567
67	8	0	3.040376	1.752313	0.367821
68	6	0	3.143030	3.083007	0.542391
69	8	0	3.626151	3.822668	-0.289694
70	6	0	2.582721	3.539187	1.868186
71	1	0	2.315462	2.651766	2.447558
72	6	0	3.637729	4.358835	2.611528
73	1	0	3.227412	4.712124	3.561627
74	1	0	4.532585	3.765874	2.822120
75	1	0	3.930401	5.230032	2.017507
76	6	0	1.327031	4.375631	1.595465
77	1	0	0.564240	3.798839	1.064856
78	1	0	0.904801	4.720654	2.543540
79	1	0	1.582266	5.251836	0.990728

Final Gibbs Free Energy = Single Point Energy + Thermal correction to Gibbs Free Energy

Single Point Energy calculated under M06-2X/6-311+G**:
SCF Done: E(RM062X) = -2443.11674888 A.U.
SMD-CDS (non-electrostatic) energy (kcal/mol) = 12.62
(included in total energy above)

Thermal corrections calculated under M06-2X/6-31G*:
Zero-point correction= 0.594547 (Hartree/Particle)
Thermal correction to Energy= 0.636140
Thermal correction to Enthalpy= 0.637084
Thermal correction to Gibbs Free Energy= 0.523752

Low frequencies --- -302.3920 -7.6231 -0.0024 -0.0017 -0.0005 2.1948
Low frequencies --- 5.9226 22.7970 31.5460

18. M06-2X results for TScp-II

Center Number	Atomic Number	Atomic Type	Coordinates (Angstroms) X	Y	Z
1	6	0	-0.967293	-3.933285	0.730927
2	6	0	-0.113554	-2.870484	0.946063
3	6	0	-0.422755	-1.788748	1.786053
4	6	0	-1.648175	-1.765714	2.446619
5	6	0	-2.524677	-2.851485	2.242554
6	6	0	-2.198054	-3.908053	1.403525
7	1	0	-0.697142	-4.743231	0.060119
8	1	0	-3.480078	-2.830924	2.757897
9	1	0	-2.907954	-4.717855	1.269073
10	6	0	1.703934	-1.311461	0.925953
11	6	0	2.928432	-0.635110	0.701995
12	6	0	3.085784	0.598436	1.417556
13	6	0	2.105570	1.095604	2.243718
14	6	0	0.852078	0.423069	2.449440
15	6	0	0.718156	-0.839048	1.767228
16	1	0	2.258707	2.041966	2.753688
17	6	0	1.228111	-2.620381	0.346758
18	8	0	1.811565	-3.327630	-0.453390
19	8	0	-2.045104	-0.765516	3.263221
20	1	0	-1.319822	-0.065071	3.284828
21	8	0	-0.062244	0.918093	3.177741
22	6	0	4.341023	1.392245	1.247542
23	6	0	3.920281	-1.089299	-0.193604
24	1	0	3.811149	-2.080754	-0.623347
25	6	0	5.313945	-0.597872	0.007608
26	6	0	5.559668	0.666197	0.733271
27	8	0	4.402736	2.578634	1.516328
28	8	0	5.753098	-0.603473	1.365249
29	1	0	6.062834	-0.918062	-0.712459
30	6	0	6.784591	1.495669	0.488228
31	1	0	6.573311	2.268636	-0.255626
32	1	0	7.102165	1.984615	1.413111
33	1	0	7.592788	0.859541	0.121226
34	6	0	2.610882	1.783669	-1.348668
35	6	0	1.430201	1.018044	-1.396487
36	6	0	1.328971	-0.224944	-1.991522
37	6	0	2.492090	-0.880949	-2.509664
38	6	0	3.731162	-0.178132	-2.230408
39	6	0	3.752778	1.183395	-1.828912
40	1	0	2.607513	2.784915	-0.929573
41	1	0	4.631280	-0.624492	-2.645900
42	1	0	4.704886	1.707429	-1.806185
43	6	0	-0.790971	0.193462	-1.079814
44	6	0	-2.110078	-0.012642	-0.715008
45	6	0	-2.713138	-1.203763	-1.170161
46	6	0	-1.984385	-2.134830	-1.908080
47	6	0	-0.634542	-1.934881	-2.231231
48	6	0	-0.047827	-0.733741	-1.829534
49	1	0	-2.465905	-3.053218	-2.230456
50	6	0	0.115638	1.336583	-0.749475
51	8	0	-0.118324	2.297697	-0.040382
52	8	0	2.475513	-2.011121	-3.084885
53	8	0	0.025633	-2.905101	-2.891288
54	6	0	-4.153254	-1.480593	-0.931920
55	6	0	-2.840475	1.024256	0.096732
56	1	0	-2.259908	1.256264	0.997074
57	6	0	-4.236707	0.630916	0.515150
58	6	0	-4.912149	-0.579837	0.008890
59	8	0	-4.734371	-2.398299	-1.487940
60	8	0	-4.311508	-0.534419	1.314021
61	1	0	-4.866030	1.457783	0.843297
62	6	0	-6.407434	-0.693735	-0.021403

63	1	0	-6.785847	-0.444794	-1.016255
64	1	0	-6.713475	-1.714931	0.219928
65	1	0	-6.843440	-0.006401	0.706862
66	1	0	0.987128	-2.621256	-3.022006
67	8	0	-2.933118	2.205950	-0.715693
68	6	0	-2.883964	3.392922	-0.073314
69	8	0	-2.856893	3.484736	1.134828
70	6	0	-2.871132	4.545276	-1.050294
71	1	0	-3.710091	4.373604	-1.735130
72	6	0	-3.055765	5.867025	-0.317147
73	1	0	-3.086224	6.688695	-1.037778
74	1	0	-3.984466	5.878888	0.259960
75	1	0	-2.223453	6.044073	0.371222
76	6	0	-1.570554	4.520156	-1.865450
77	1	0	-1.443879	3.569297	-2.390087
78	1	0	-1.592569	5.324769	-2.605667
79	1	0	-0.705817	4.676490	-1.212615

Final Gibbs Free Energy = Single Point Energy + Thermal correction to Gibbs Free Energy

Single Point Energy calculated under M06-2X/6-311+G**:
SCF Done: E(RM062X) = -2443.10826483 A.U.
SMD-CDS (non-electrostatic) energy (kcal/mol) = 13.07
(included in total energy above)

Thermal corrections calculated under M06-2X/6-31G*:
Zero-point correction= 0.593759 (Hartree/Particle)
Thermal correction to Energy= 0.635442
Thermal correction to Enthalpy= 0.636386
Thermal correction to Gibbs Free Energy= 0.522217

Low frequencies --- -355.3544 -10.6423 -2.7521 -0.0021 -0.0016 -0.0007
Low frequencies --- 3.6643 22.5368 31.3552

19. M06-2X results for TScp-III

Center Number	Atomic Number	Atomic Type	Coordinates (Angstroms) X	Y	Z
1	6	0	-7.034369	-1.483660	-0.602579
2	6	0	-5.855963	-0.923591	-0.153237
3	6	0	-5.590989	0.454756	-0.195391
4	6	0	-6.550092	1.318500	-0.719536
5	6	0	-7.757686	0.756717	-1.182949
6	6	0	-7.997638	-0.608958	-1.127159
7	1	0	-7.202329	-2.554782	-0.551847
8	1	0	-8.503932	1.431833	-1.590869
9	1	0	-8.941282	-0.997623	-1.495865
10	6	0	-3.672497	-0.516007	0.733346
11	6	0	-2.370955	-0.635340	1.285079
12	6	0	-1.671185	0.612780	1.466263
13	6	0	-2.219953	1.816309	1.101106
14	6	0	-3.527877	1.929891	0.506413
15	6	0	-4.240399	0.688446	0.369185
16	1	0	-1.657752	2.733306	1.252251
17	6	0	-4.670262	-1.605252	0.442793
18	8	0	-4.549289	-2.798140	0.654097
19	8	0	-6.392450	2.658070	-0.809425
20	1	0	-5.480888	2.895114	-0.449678
21	8	0	-4.004744	3.052620	0.160445
22	6	0	-0.303071	0.622553	2.063665
23	6	0	-1.755964	-1.870103	1.548096
24	1	0	-2.375017	-2.760078	1.567909
25	6	0	-0.560546	-1.884378	2.437272
26	6	0	0.178152	-0.633365	2.747910
27	8	0	0.428408	1.595435	1.994064
28	8	0	-0.768892	-1.186727	3.665785
29	1	0	-0.012252	-2.819446	2.531136
30	6	0	1.599756	-0.630213	3.233062
31	1	0	2.278334	-0.263650	2.460556
32	1	0	1.684436	0.036225	4.096527
33	1	0	1.898019	-1.638456	3.529062
34	6	0	-0.819361	-2.585783	-0.347816
35	6	0	0.060952	-1.498455	-0.534815
36	6	0	-0.356298	-0.303960	-1.074980
37	6	0	-1.685743	-0.170654	-1.627641
38	6	0	-2.432138	-1.410467	-1.738321
39	6	0	-2.009935	-2.564309	-1.141679
40	1	0	-0.441696	-3.525014	0.045952
41	1	0	-3.373065	-1.367192	-2.281001
42	1	0	-2.625574	-3.457153	-1.189928
43	6	0	1.870443	0.050989	-0.448711
44	6	0	3.067977	0.704106	-0.222409
45	6	0	3.113418	2.073814	-0.554257
46	6	0	1.987749	2.725191	-1.051701
47	6	0	0.775865	2.051492	-1.267983
48	6	0	0.739961	0.685838	-0.988157
49	1	0	2.043559	3.783921	-1.285633
50	6	0	1.486923	-1.361041	-0.114133
51	8	0	2.179510	-2.216243	0.407878
52	8	0	-2.168354	0.935463	-1.997410
53	8	0	-0.277737	2.754054	-1.729592
54	6	0	4.375645	2.847149	-0.431170
55	6	0	4.259845	-0.057924	0.291426
56	1	0	3.967796	-0.670031	1.149349
57	6	0	5.412300	0.814241	0.719003
58	6	0	5.493258	2.249416	0.381496
59	8	0	4.526410	3.932285	-0.967523
60	8	0	5.051804	1.796498	1.674007
61	1	0	6.346312	0.290005	0.918570
62	6	0	6.805203	2.975544	0.351858

63	1	0	7.530705	2.449042	0.975930
64	1	0	7.186519	3.026637	-0.671039
65	1	0	6.682617	3.995142	0.726623
66	1	0	-1.059632	2.135196	-1.844036
67	8	0	4.676307	-0.940084	-0.763773
68	6	0	5.172142	-2.139386	-0.392059
69	8	0	5.402883	-2.422138	0.764359
70	6	0	5.362879	-3.055554	-1.576208
71	1	0	5.892436	-3.928964	-1.186087
72	6	0	3.987935	-3.485848	-2.102974
73	1	0	4.119964	-4.184213	-2.934325
74	1	0	3.399297	-3.981718	-1.325619
75	1	0	3.426358	-2.618880	-2.466399
76	6	0	6.195836	-2.388849	-2.672414
77	1	0	7.161341	-2.044093	-2.290892
78	1	0	6.380559	-3.109056	-3.474423
79	1	0	5.664401	-1.532623	-3.097673

Final Gibbs Free Energy = Single Point Energy + Thermal correction to Gibbs Free Energy

Single Point Energy calculated under M06-2X/6-311+G**:
SCF Done: E(RM062X) = -2443.10709348   A.U.
 SMD-CDS (non-electrostatic) energy   (kcal/mol) =   13.54
 (included in total energy above)

Thermal corrections calculated under M06-2X/6-31G*:
Zero-point correction=   0.595348 (Hartree/Particle)
 Thermal correction to Energy=   0.636945
 Thermal correction to Enthalpy=   0.637889
 Thermal correction to Gibbs Free Energy=   0.523117

Low frequencies --- -278.2027   -0.0008   0.0004   0.0008   6.7394   8.9669
 Low frequencies ---   13.8789   23.9392   26.3737

20. M06-2X results for TScp-IV

Center Number	Atomic Number	Atomic Type	Coordinates (Angstroms) X	Y	Z
1	6	0	3.903162	4.045518	0.824608
2	6	0	3.339088	2.909285	0.278567
3	6	0	2.257232	2.940154	-0.616155
4	6	0	1.743926	4.170368	-1.021956
5	6	0	2.298183	5.335093	-0.455365
6	6	0	3.349742	5.277623	0.449826
7	1	0	4.742357	3.980849	1.509977
8	1	0	1.885720	6.291827	-0.762455
9	1	0	3.750230	6.200149	0.857168
10	6	0	2.798329	0.673223	-0.378129
11	6	0	2.763038	-0.726760	-0.578623
12	6	0	1.697702	-1.208478	-1.416609
13	6	0	0.791723	-0.360744	-1.992316
14	6	0	0.858232	1.075473	-1.833789
15	6	0	1.918077	1.547001	-0.990940
16	1	0	-0.003648	-0.759030	-2.615758
17	6	0	3.765142	1.490028	0.444730
18	8	0	4.719988	1.088208	1.083093
19	8	0	0.761796	4.312264	-1.940706
20	1	0	0.423308	3.399226	-2.193237
21	8	0	0.027639	1.838217	-2.411769
22	6	0	1.545578	-2.675451	-1.660426
23	6	0	3.671892	-1.618781	0.012488
24	1	0	4.548109	-1.212265	0.504128
25	6	0	3.805400	-2.988307	-0.552750
26	6	0	2.747789	-3.552718	-1.419696
27	8	0	0.499919	-3.158512	-2.054656
28	8	0	3.945804	-3.004959	-1.975767
29	1	0	4.446529	-3.687928	-0.022682
30	6	0	2.546846	-5.028882	-1.588511
31	1	0	1.711269	-5.364371	-0.970549
32	1	0	2.320237	-5.254702	-2.634324
33	1	0	3.452334	-5.563975	-1.294171
34	6	0	1.952649	-0.108984	2.561056
35	6	0	0.793404	-0.633924	1.971972
36	6	0	0.657857	-1.945108	1.548062
37	6	0	1.749145	-2.858922	1.644355
38	6	0	2.985664	-2.260125	2.099833
39	6	0	3.034715	-0.960469	2.658769
40	1	0	1.981665	0.915979	2.917382
41	1	0	3.824999	-2.935385	2.247521
42	1	0	3.968651	-0.616812	3.092647
43	6	0	-1.407268	-0.932691	1.086451
44	6	0	-2.719537	-0.792844	0.663999
45	6	0	-3.315712	-1.920870	0.069197
46	6	0	-2.617017	-3.123079	-0.045001
47	6	0	-1.307596	-3.266494	0.430156
48	6	0	-0.697635	-2.140248	0.991491
49	1	0	-3.099825	-3.976588	-0.510852
50	6	0	-0.490902	0.087263	1.688318
51	8	0	-0.708522	1.266995	1.893577
52	8	0	1.681461	-4.082292	1.292485
53	8	0	-0.701208	-4.464478	0.317257
54	6	0	-4.679428	-1.844777	-0.512583
55	6	0	-3.441662	0.512435	0.858947
56	1	0	-3.318340	0.832963	1.898307
57	6	0	-4.921010	0.454729	0.571405
58	6	0	-5.557276	-0.690922	-0.107181
59	8	0	-5.097268	-2.678814	-1.299409
60	8	0	-5.614757	-0.526956	1.321216
61	1	0	-5.417346	1.421964	0.498838
62	6	0	-6.856942	-0.546555	-0.841424

63	1	0	-6.672094	-0.386578	-1.906818
64	1	0	-7.459016	-1.451753	-0.726689
65	1	0	-7.410417	0.307075	-0.444026
66	1	0	0.239763	-4.392827	0.685261
67	8	0	-2.823890	1.493125	0.013831
68	6	0	-2.719501	2.737550	0.533793
69	8	0	-3.288143	3.070034	1.551360
70	6	0	-1.836712	3.615296	-0.314548
71	1	0	-0.898695	3.059987	-0.435953
72	6	0	-2.474625	3.799179	-1.697650
73	1	0	-1.823150	4.419431	-2.319330
74	1	0	-2.622769	2.840164	-2.199910
75	1	0	-3.444095	4.300962	-1.607047
76	6	0	-1.571239	4.943559	0.379340
77	1	0	-1.095739	4.794774	1.353267
78	1	0	-0.909648	5.555475	-0.240360
79	1	0	-2.505642	5.492879	0.533311

Final Gibbs Free Energy = Single Point Energy + Thermal correction to Gibbs Free Energy

Single Point Energy calculated under M06-2X/6-311+G**:
SCF Done: E(RM062X) = -2443.10508880 A.U.
SMD-CDS (non-electrostatic) energy (kcal/mol) = 12.91
(included in total energy above)

Thermal corrections calculated under M06-2X/6-31G*:
Zero-point correction= 0.594702 (Hartree/Particle)
Thermal correction to Energy= 0.636333
Thermal correction to Enthalpy= 0.637278
Thermal correction to Gibbs Free Energy= 0.523353

Low frequencies --- -329.2268 -5.6913 -0.0028 -0.0021 -0.0009 4.0065
Low frequencies --- 7.3811 26.2491 28.2443

21. M06-2X results for TScp-V

Center Number	Atomic Number	Atomic Type	X	Y	Z
			Coordinates (Angstroms)		
1	6	0	-0.889904	2.741415	-1.583437
2	6	0	0.317885	2.218895	-1.169949
3	6	0	1.153430	2.845771	-0.231125
4	6	0	0.745989	4.042788	0.350063
5	6	0	-0.493053	4.582236	-0.054513
6	6	0	-1.290447	3.953986	-1.002491
7	1	0	-1.510784	2.217802	-2.303001
8	1	0	-0.811083	5.514790	0.402494
9	1	0	-2.238390	4.403962	-1.282115
10	6	0	2.199948	0.813810	-0.730667
11	6	0	3.177278	-0.212382	-0.761178
12	6	0	4.383733	0.073451	-0.032572
13	6	0	4.532758	1.225931	0.698333
14	6	0	3.502668	2.236562	0.791932
15	6	0	2.341495	1.986137	-0.012273
16	1	0	5.467614	1.415939	1.217683
17	6	0	0.922850	0.905441	-1.520844
18	8	0	0.463427	0.072087	-2.282733
19	8	0	1.463748	4.705748	1.285734
20	1	0	2.330970	4.213853	1.429016
21	8	0	3.661228	3.286911	1.486259
22	6	0	5.559205	-0.836314	-0.180832
23	6	0	2.970314	-1.455397	-1.383180
24	1	0	2.124105	-1.542582	-2.056069
25	6	0	4.154680	-2.296826	-1.740059
26	6	0	5.490354	-1.939904	-1.225517
27	8	0	6.583584	-0.687655	0.460862
28	8	0	4.796454	-3.089450	-0.739603
29	1	0	4.094345	-2.792206	-2.706429
30	6	0	6.756505	-2.197975	-1.988271
31	1	0	7.055125	-1.302055	-2.539521
32	1	0	7.561171	-2.466723	-1.298655
33	1	0	6.599748	-3.015894	-2.695094
34	6	0	1.905171	-2.732301	0.135767
35	6	0	0.774067	-1.902257	0.301900
36	6	0	0.762361	-0.839652	1.175492
37	6	0	1.896486	-0.581036	2.032647
38	6	0	2.883547	-1.637200	2.077672
39	6	0	2.862935	-2.683266	1.200911
40	1	0	1.847228	-3.604839	-0.508486
41	1	0	3.656444	-1.551321	2.835978
42	1	0	3.621803	-3.452174	1.265438
43	6	0	-1.316966	-0.755757	0.089848
44	6	0	-2.566454	-0.283759	-0.267589
45	6	0	-2.995998	0.915627	0.343957
46	6	0	-2.220563	1.562023	1.297414
47	6	0	-0.963949	1.062983	1.673322
48	6	0	-0.516120	-0.102547	1.048145
49	1	0	-2.565660	2.495706	1.732018
50	6	0	-0.503970	-1.898449	-0.472039
51	8	0	-0.785814	-2.641762	-1.391212
52	8	0	2.023626	0.478316	2.710844
53	8	0	-0.248439	1.754150	2.582052
54	6	0	-4.245941	1.576152	-0.114964
55	6	0	-3.520668	-1.016661	-1.177754
56	1	0	-3.008251	-1.779248	-1.765174
57	6	0	-4.308923	-0.069366	-2.053124
58	6	0	-4.727485	1.236711	-1.505529
59	8	0	-4.850473	2.391218	0.561024
60	8	0	-3.654640	1.115526	-2.456560
61	1	0	-4.950592	-0.531659	-2.800740
62	6	0	-5.964613	1.939664	-1.978138

63	1	0	-6.808410	1.692944	-1.328832
64	1	0	-5.816519	3.022779	-1.956838
65	1	0	-6.197759	1.628496	-2.998675
66	1	0	0.662231	1.333467	2.675498
67	8	0	-4.562699	-1.634206	-0.386380
68	6	0	-4.270074	-2.767735	0.278549
69	8	0	-3.200996	-3.329657	0.182372
70	6	0	-5.419118	-3.194078	1.161397
71	1	0	-6.321989	-3.167028	0.541127
72	6	0	-5.188628	-4.599579	1.699144
73	1	0	-6.040991	-4.904707	2.312072
74	1	0	-5.068433	-5.323634	0.888558
75	1	0	-4.288735	-4.628814	2.321188
76	6	0	-5.570362	-2.166354	2.292476
77	1	0	-5.760898	-1.163710	1.900673
78	1	0	-6.405920	-2.452462	2.937030
79	1	0	-4.660955	-2.135215	2.902051

Final Gibbs Free Energy = Single Point Energy + Thermal correction to Gibbs Free Energy

Single Point Energy calculated under M06-2X/6-311+G**:
SCF Done: E(RM062X) = -2443.09675587   A.U.
SMD-CDS (non-electrostatic) energy   (kcal/mol) =   14.07
(included in total energy above)

Thermal corrections calculated under M06-2X/6-31G*:
Zero-point correction=   0.593610 (Hartree/Particle)
Thermal correction to Energy=   0.635693
Thermal correction to Enthalpy=   0.636637
Thermal correction to Gibbs Free Energy=   0.518655

Low frequencies --- -299.2411 -16.9737   -3.0684   -0.0026   -0.0016   -0.0013
Low frequencies ---   7.8706   11.2053   19.5869

22. M06-2X results for TScp-VI

Center Number	Atomic Number	Atomic Type	Coordinates (Angstroms) X	Y	Z
1	6	0	1.392825	-0.559598	2.499790
2	6	0	0.208830	-0.061521	1.994390
3	6	0	-0.013694	1.305017	1.757395
4	6	0	0.986561	2.222850	2.070655
5	6	0	2.215494	1.720659	2.547528
6	6	0	2.418885	0.362976	2.754556
7	1	0	1.522363	-1.621202	2.685217
8	1	0	3.003126	2.436898	2.763789
9	1	0	3.377788	0.016642	3.128319
10	6	0	-1.989229	0.231685	1.090897
11	6	0	-3.313838	0.054082	0.622501
12	6	0	-4.040305	1.264488	0.357867
13	6	0	-3.450032	2.498071	0.472832
14	6	0	-2.073156	2.677527	0.863156
15	6	0	-1.385175	1.468780	1.217567
16	1	0	-4.040499	3.393729	0.304987
17	6	0	-1.038064	-0.800012	1.646265
18	8	0	-1.261972	-1.980390	1.838673
19	8	0	0.848143	3.561227	1.949360
20	1	0	-0.058764	3.754181	1.556840
21	8	0	-1.548758	3.829546	0.932189
22	6	0	-5.523323	1.227096	0.166540
23	6	0	-3.890583	-1.216363	0.410329
24	1	0	-3.447099	-2.072919	0.908964
25	6	0	-5.349350	-1.322514	0.118624
26	6	0	-6.207813	-0.115963	0.068668
27	8	0	-6.193344	2.242471	0.118078
28	8	0	-5.807033	-0.792303	-1.122263
29	1	0	-5.818674	-2.264353	0.392439
30	6	0	-7.667337	-0.156472	0.408286
31	1	0	-7.821888	0.154193	1.444960
32	1	0	-8.222648	0.521847	-0.244671
33	1	0	-8.047302	-1.172014	0.278685
34	6	0	-2.012241	-0.292036	-2.534305
35	6	0	-0.877528	-0.829323	-1.903990
36	6	0	-0.876031	-2.005845	-1.176431
37	6	0	-2.080714	-2.755586	-0.995451
38	6	0	-3.264602	-2.128630	-1.548674
39	6	0	-3.191545	-0.988119	-2.390405
40	1	0	-1.939257	0.611803	-3.129912
41	1	0	-4.158806	-2.745879	-1.568402
42	1	0	-4.097899	-0.639666	-2.871792
43	6	0	1.337610	-1.234363	-1.101374
44	6	0	2.668712	-1.145153	-0.729946
45	6	0	3.143282	-2.137695	0.147860
46	6	0	2.316920	-3.179447	0.569511
47	6	0	0.979767	-3.272075	0.164988
48	6	0	0.488624	-2.262993	-0.668160
49	1	0	2.706700	-3.922296	1.259107
50	6	0	0.523972	-0.294338	-1.935116
51	8	0	0.905856	0.690492	-2.538822
52	8	0	-2.145081	-3.854131	-0.356153
53	8	0	0.227657	-4.290748	0.621936
54	6	0	4.488445	-2.019420	0.761231
55	6	0	3.527744	-0.016657	-1.231457
56	1	0	3.421670	0.058082	-2.317364
57	6	0	4.995862	-0.169760	-0.914828
58	6	0	5.499407	-1.144680	0.073679
59	8	0	4.777220	-2.589865	1.801155
60	8	0	5.563318	-1.394849	-1.341491
61	1	0	5.605914	0.713027	-1.102544
62	6	0	6.809497	-0.948679	0.776326

63	1	0	6.647442	-0.474131	1.747651
64	1	0	7.299466	-1.912279	0.939217
65	1	0	7.459095	-0.311618	0.172257
66	1	0	-0.717981	-4.183327	0.274702
67	8	0	3.053667	1.211394	-0.648231
68	6	0	3.204567	2.316305	-1.410146
69	8	0	3.777701	2.299553	-2.479401
70	6	0	2.562601	3.532015	-0.791093
71	1	0	2.688150	3.456270	0.293715
72	6	0	3.221924	4.802708	-1.314995
73	1	0	2.773991	5.674775	-0.830787
74	1	0	4.296998	4.808723	-1.113440
75	1	0	3.073996	4.895530	-2.395184
76	6	0	1.061236	3.510144	-1.113521
77	1	0	0.567299	2.631789	-0.685631
78	1	0	0.591104	4.407665	-0.702752
79	1	0	0.907486	3.502361	-2.197909

Final Gibbs Free Energy = Single Point Energy + Thermal correction to Gibbs Free Energy

Single Point Energy calculated under M06-2X/6-311+G**:
SCF Done: E(RM062X) = -2443.09967062   A.U.
SMD-CDS (non-electrostatic) energy   (kcal/mol) =   13.07
(included in total energy above)

Thermal corrections calculated under M06-2X/6-31G*:
Zero-point correction=   0.595186 (Hartree/Particle)
Thermal correction to Energy=   0.636573
Thermal correction to Enthalpy=   0.637517
Thermal correction to Gibbs Free Energy=   0.524112

Low frequencies --- -375.2528   -0.9947   -0.0021   -0.0011   -0.0003   7.0558
Low frequencies ---   10.5613   21.1831   29.0942

23. M06-2X results for TScp-VII

Center Number	Atomic Number	Atomic Type	Coordinates (Angstroms) X	Y	Z
1	6	0	0.486897	-2.981630	2.524291
2	6	0	-0.450444	-2.017760	2.218568
3	6	0	-0.174271	-0.637593	2.238662
4	6	0	1.107290	-0.201986	2.582445
5	6	0	2.070270	-1.180838	2.903338
6	6	0	1.773569	-2.536391	2.867545
7	1	0	0.239792	-4.037817	2.483608
8	1	0	3.067691	-0.840818	3.168997
9	1	0	2.551541	-3.255013	3.104897
10	6	0	-2.360538	-0.820285	1.446545
11	6	0	-3.551421	-0.441504	0.796479
12	6	0	-3.776793	0.965686	0.647786
13	6	0	-2.833645	1.885366	1.037393
14	6	0	-1.548791	1.500368	1.564107
15	6	0	-1.378739	0.089887	1.793390
16	1	0	-3.025527	2.946516	0.909415
17	6	0	-1.863969	-2.200623	1.774059
18	8	0	-2.487168	-3.246501	1.736028
19	8	0	1.479169	1.090680	2.606263
20	1	0	0.701592	1.659657	2.301483
21	8	0	-0.645881	2.360166	1.800645
22	6	0	-5.077696	1.450036	0.100747
23	6	0	-4.302380	-1.369146	0.042065
24	1	0	-4.190892	-2.417990	0.290002
25	6	0	-5.656659	-0.978676	-0.461698
26	6	0	-6.101192	0.427055	-0.358685
27	8	0	-5.355671	2.634983	0.050747
28	8	0	-5.756718	-0.152705	-1.617744
29	1	0	-6.411349	-1.760547	-0.416071
30	6	0	-7.540734	0.804983	-0.171384
31	1	0	-7.750823	0.977528	0.887566
32	1	0	-7.760949	1.722520	-0.723387
33	1	0	-8.183238	0.001295	-0.537792
34	6	0	-1.135388	-2.779701	-1.162538
35	6	0	-0.483158	-1.535301	-1.101060
36	6	0	-1.083545	-0.332906	-1.432845
37	6	0	-2.446263	-0.292833	-1.875266
38	6	0	-3.152822	-1.572823	-1.828173
39	6	0	-2.452774	-2.784392	-1.562516
40	1	0	-0.606149	-3.691452	-0.903489
41	1	0	-4.085967	-1.620029	-2.381155
42	1	0	-3.000905	-3.719562	-1.632863
43	6	0	1.099413	0.219718	-0.771143
44	6	0	2.209351	0.996124	-0.472836
45	6	0	2.086871	2.384312	-0.670183
46	6	0	0.881778	2.938920	-1.104764
47	6	0	-0.241261	2.148479	-1.373379
48	6	0	-0.113829	0.763716	-1.228901
49	1	0	0.806213	4.013857	-1.237960
50	6	0	0.929042	-1.265320	-0.679247
51	8	0	1.756295	-2.098966	-0.351270
52	8	0	-3.029523	0.777833	-2.219343
53	8	0	-1.388192	2.754890	-1.740764
54	6	0	3.247680	3.289802	-0.485174
55	6	0	3.477403	0.348294	0.010775
56	1	0	3.239705	-0.347098	0.820718
57	6	0	4.524775	1.312832	0.508217
58	6	0	4.446500	2.765735	0.261701
59	8	0	3.257344	4.430183	-0.920812
60	8	0	4.085994	2.189354	1.529779
61	1	0	5.513165	0.880339	0.661319
62	6	0	5.672217	3.629881	0.254087

63	1	0	6.017667	3.784892	-0.771389
64	1	0	5.451863	4.605034	0.696171
65	1	0	6.466504	3.146657	0.827318
66	1	0	-2.077089	2.049363	-1.950141
67	8	0	4.032137	-0.404672	-1.080724
68	6	0	4.608725	-1.586114	-0.770043
69	8	0	4.765168	-1.962325	0.371728
70	6	0	5.015739	-2.340376	-2.014272
71	1	0	5.611965	-1.641492	-2.613036
72	6	0	5.849400	-3.561675	-1.649861
73	1	0	6.170448	-4.075014	-2.560319
74	1	0	6.738405	-3.284237	-1.076976
75	1	0	5.259710	-4.262094	-1.050141
76	6	0	3.767462	-2.724982	-2.820884
77	1	0	3.170875	-1.847510	-3.084439
78	1	0	4.074248	-3.225026	-3.743865
79	1	0	3.139464	-3.415341	-2.248841

Final Gibbs Free Energy = Single Point Energy + Thermal correction to Gibbs Free Energy

Single Point Energy calculated under M06-2X/6-311+G**:
SCF Done: E(RM062X) = -2443.09788909 A.U.
SMD-CDS (non-electrostatic) energy (kcal/mol) = 13.18
(included in total energy above)

Thermal corrections calculated under M06-2X/6-31G*:
Zero-point correction= 0.594014 (Hartree/Particle)
Thermal correction to Energy= 0.635703
Thermal correction to Enthalpy= 0.636647
Thermal correction to Gibbs Free Energy= 0.522628

Low frequencies --- -400.9130 -5.6703 -0.0020 -0.0014 -0.0008 7.5804
Low frequencies --- 9.9111 28.8370 30.4317

24. M06-2X results for TScp-VIII

Center Number	Atomic Number	Atomic Type	Coordinates (Angstroms) X	Y	Z
1	6	0	-0.069529	-3.335231	2.530741
2	6	0	-0.592064	-2.104208	2.188172
3	6	0	0.174261	-0.924626	2.162621
4	6	0	1.537268	-0.983442	2.441498
5	6	0	2.078766	-2.235094	2.798240
6	6	0	1.296580	-3.381884	2.847862
7	1	0	-0.687569	-4.227725	2.528496
8	1	0	3.141857	-2.281857	3.016536
9	1	0	1.759114	-4.326664	3.115507
10	6	0	-1.926243	-0.321755	1.329982
11	6	0	-2.907534	0.467440	0.698863
12	6	0	-2.639262	1.873527	0.635001
13	6	0	-1.461332	2.405155	1.101662
14	6	0	-0.394639	1.580370	1.603652
15	6	0	-0.687659	0.177736	1.687665
16	1	0	-1.307760	3.480309	1.089218
17	6	0	-1.969475	-1.776071	1.715306
18	8	0	-2.938586	-2.514509	1.709335
19	8	0	2.371643	0.078504	2.358098
20	1	0	1.818700	0.902456	2.194872
21	8	0	0.708358	2.071213	1.994506
22	6	0	-3.727578	2.801719	0.209045
23	6	0	-3.986436	-0.122137	0.004342
24	1	0	-4.351983	-1.079932	0.354317
25	6	0	-4.962297	0.749501	-0.701515
26	6	0	-4.894115	2.228626	-0.559699
27	8	0	-3.699483	3.992128	0.464222
28	8	0	-4.440083	1.573038	-1.742126
29	1	0	-5.932014	0.312869	-0.928000
30	6	0	-6.111704	3.093201	-0.689033
31	1	0	-6.519921	3.317431	0.299859
32	1	0	-5.853354	4.034866	-1.180417
33	1	0	-6.870025	2.575172	-1.280002
34	6	0	-3.090556	-1.095425	-1.815166
35	6	0	-1.712396	-1.225815	-1.560793
36	6	0	-1.117273	-2.388882	-1.123549
37	6	0	-1.900630	-3.591227	-0.937631
38	6	0	-3.287260	-3.490061	-1.330925
39	6	0	-3.841136	-2.318081	-1.765640
40	1	0	-3.438261	-0.257811	-2.408044
41	1	0	-3.883931	-4.393518	-1.251016
42	1	0	-4.891903	-2.292464	-2.043441
43	6	0	0.618928	-0.806793	-1.197214
44	6	0	1.909081	-0.302280	-1.148573
45	6	0	2.928009	-1.207155	-0.784449
46	6	0	2.650737	-2.532088	-0.469913
47	6	0	1.347033	-3.041843	-0.551826
48	6	0	0.329182	-2.159678	-0.922437
49	1	0	3.460667	-3.202120	-0.196464
50	6	0	-0.676504	-0.144295	-1.582873
51	8	0	-0.881308	1.027290	-1.838667
52	8	0	-1.422028	-4.656385	-0.452309
53	8	0	1.156050	-4.349557	-0.296314
54	6	0	4.347708	-0.770637	-0.804205
55	6	0	2.270224	1.118750	-1.492899
56	1	0	1.479788	1.588768	-2.077125
57	6	0	3.606474	1.205088	-2.203142
58	6	0	4.700206	0.297162	-1.807419
59	8	0	5.203884	-1.268864	-0.093645
60	8	0	3.981172	0.134797	-3.044900
61	1	0	3.882087	2.199004	-2.553348
62	6	0	6.143224	0.690527	-1.906026

63	1	0	6.488840	1.084981	-0.947014
64	1	0	6.757674	-0.175915	-2.165276
65	1	0	6.264898	1.459744	-2.671576
66	1	0	0.167329	-4.548194	-0.318297
67	8	0	2.506038	1.892872	-0.295259
68	6	0	1.885591	3.087252	-0.176294
69	8	0	0.903016	3.380043	-0.825152
70	6	0	2.639067	4.047822	0.721357
71	1	0	3.340254	4.518372	0.013857
72	6	0	1.708465	5.129047	1.261745
73	1	0	2.291152	5.885395	1.795004
74	1	0	1.160349	5.622711	0.455746
75	1	0	0.985850	4.696814	1.960416
76	6	0	3.468869	3.390295	1.823007
77	1	0	4.126286	2.611513	1.429029
78	1	0	4.093196	4.152644	2.298476
79	1	0	2.825000	2.951536	2.589135

Final Gibbs Free Energy = Single Point Energy + Thermal correction to Gibbs Free Energy

Single Point Energy calculated under M06-2X/6-311+G**:
SCF Done: E(RM062X) = -2443.09395221   A.U.
 SMD-CDS (non-electrostatic) energy   (kcal/mol) =   12.71
 (included in total energy above)

Thermal corrections calculated under M06-2X/6-31G*:
Zero-point correction=   0.594747 (Hartree/Particle)
 Thermal correction to Energy=   0.636281
 Thermal correction to Enthalpy=   0.637225
 Thermal correction to Gibbs Free Energy=   0.524169

Low frequencies --- -430.4059   -2.6523   -0.0021   -0.0015   -0.0008   3.7429
 Low frequencies ---   11.7342   21.3359   35.6511

25. M06-2X results for INT-I

Center Number	Atomic Number	Atomic Type	Coordinates (Angstroms) X	Y	Z
1	6	0	1.615257	-1.436465	2.030265
2	6	0	0.280064	-1.236781	1.733181
3	6	0	-0.649641	-2.281479	1.612717
4	6	0	-0.224757	-3.601064	1.764287
5	6	0	1.136900	-3.816399	2.063040
6	6	0	2.035380	-2.763130	2.199163
7	1	0	2.309294	-0.602753	2.096320
8	1	0	1.473302	-4.842310	2.181824
9	1	0	3.075186	-2.979723	2.423482
10	6	0	-1.831155	-0.330440	1.064037
11	6	0	-2.868265	0.495985	0.633188
12	6	0	-4.117989	-0.151205	0.476794
13	6	0	-4.271588	-1.520915	0.669582
14	6	0	-3.195152	-2.383120	1.026711
15	6	0	-1.964993	-1.714052	1.240019
16	1	0	-5.253481	-1.964001	0.527234
17	6	0	-0.415033	0.032278	1.387324
18	8	0	0.085309	1.145557	1.347329
19	8	0	-1.045509	-4.663323	1.630253
20	1	0	-1.997464	-4.315425	1.436374
21	8	0	-3.346465	-3.664013	1.156020
22	6	0	-5.306270	0.636434	0.058702
23	6	0	-2.658620	1.922597	0.204372
24	1	0	-1.855451	2.380107	0.786565
25	6	0	-3.915214	2.746377	0.356721
26	6	0	-5.256514	2.129565	0.263592
27	8	0	-6.299917	0.124788	-0.433963
28	8	0	-4.684137	2.513290	1.523884
29	1	0	-3.839582	3.793170	0.062500
30	6	0	-6.451093	2.910855	-0.197146
31	1	0	-6.594073	2.779496	-1.273310
32	1	0	-7.354266	2.566758	0.314123
33	1	0	-6.300307	3.971830	0.013939
34	6	0	-2.190274	2.052876	-1.346019
35	6	0	-0.983259	1.218211	-1.534108
36	6	0	-1.069311	-0.102976	-1.793581
37	6	0	-2.365198	-0.697183	-2.172299
38	6	0	-3.415784	0.263959	-2.546963
39	6	0	-3.318677	1.555973	-2.197777
40	1	0	-1.990969	3.115459	-1.509193
41	1	0	-4.280814	-0.138709	-3.064891
42	1	0	-4.123816	2.249501	-2.429223
43	6	0	1.194040	0.250792	-1.356809
44	6	0	2.525356	-0.014404	-1.106106
45	6	0	2.891957	-1.377950	-1.043713
46	6	0	1.959171	-2.387540	-1.234213
47	6	0	0.614934	-2.099897	-1.524274
48	6	0	0.249584	-0.758133	-1.605491
49	1	0	2.262670	-3.426311	-1.145062
50	6	0	0.456899	1.563335	-1.294163
51	8	0	0.921097	2.667820	-1.099272
52	8	0	-2.555786	-1.915881	-2.210282
53	8	0	-0.233232	-3.136256	-1.668872
54	6	0	4.279702	-1.765754	-0.679132
55	6	0	3.521308	1.097346	-0.907299
56	1	0	3.321972	1.890730	-1.631072
57	6	0	4.956934	0.653351	-1.066381
58	6	0	5.365629	-0.761201	-0.941416
59	8	0	4.541995	-2.849536	-0.186289
60	8	0	5.217721	-0.121069	-2.221117
61	1	0	5.706579	1.409410	-0.836326
62	6	0	6.758425	-1.149709	-0.545014

63	1	0	6.805422	-1.337589	0.530541
64	1	0	7.061187	-2.059884	-1.069570
65	1	0	7.450862	-0.343300	-0.795806
66	1	0	-1.148001	-2.794413	-1.843377
67	8	0	3.370276	1.641717	0.418760
68	6	0	3.727516	2.939533	0.560967
69	8	0	4.096131	3.616695	-0.374638
70	6	0	3.629909	3.410856	1.993348
71	1	0	4.292173	2.753068	2.570385
72	6	0	4.107792	4.853258	2.103769
73	1	0	4.091053	5.167186	3.150765
74	1	0	5.125832	4.970542	1.723294
75	1	0	3.451135	5.518944	1.534585
76	6	0	2.201628	3.239836	2.520186
77	1	0	1.907464	2.189957	2.550195
78	1	0	2.138277	3.647714	3.533006
79	1	0	1.488343	3.780531	1.887819

Final Gibbs Free Energy = Single Point Energy + Thermal correction to Gibbs Free Energy

Single Point Energy calculated under M06-2X/6-311+G**:
SCF Done: E(RM062X) = -2443.13263901   A.U.
 SMD-CDS (non-electrostatic) energy   (kcal/mol) =   12.84
 (included in total energy above)

Thermal corrections calculated under M06-2X/6-31G*:
Zero-point correction=   0.597260 (Hartree/Particle)
 Thermal correction to Energy=   0.638735
 Thermal correction to Enthalpy=   0.639679
 Thermal correction to Gibbs Free Energy=   0.525779

26. M06-2X results for INT-II

Center Number	Atomic Number	Atomic Type	Coordinates (Angstroms) X	Y	Z
1	6	0	0.282239	-4.264005	-0.273967
2	6	0	-0.451170	-3.134416	-0.588603
3	6	0	0.012549	-2.138407	-1.457492
4	6	0	1.265462	-2.268783	-2.063344
5	6	0	2.025221	-3.410992	-1.737874
6	6	0	1.545811	-4.386111	-0.867125
7	1	0	-0.102575	-5.008978	0.415696
8	1	0	3.007035	-3.515052	-2.191364
9	1	0	2.167071	-5.248650	-0.646607
10	6	0	-2.079275	-1.379631	-0.703310
11	6	0	-3.159229	-0.520112	-0.522433
12	6	0	-3.164024	0.633012	-1.339801
13	6	0	-2.085095	0.962563	-2.155092
14	6	0	-0.914644	0.163547	-2.239514
15	6	0	-0.995477	-1.062099	-1.538603
16	1	0	-2.122720	1.882071	-2.732697
17	6	0	-1.782649	-2.710140	-0.065013
18	8	0	-2.475705	-3.333275	0.722526
19	8	0	1.753676	-1.371045	-2.936885
20	1	0	1.105235	-0.552011	-2.969981
21	8	0	0.131421	0.543707	-2.916358
22	6	0	-4.325082	1.557479	-1.307118
23	6	0	-4.161274	-0.650461	0.603409
24	1	0	-4.267181	-1.697774	0.900606
25	6	0	-5.511934	-0.102365	0.207084
26	6	0	-5.619226	1.028175	-0.739405
27	8	0	-4.271563	2.704481	-1.725128
28	8	0	-5.908428	-0.321535	-1.135415
29	1	0	-6.307567	-0.206123	0.944244
30	6	0	-6.777117	1.981085	-0.701476
31	1	0	-6.532319	2.845518	-0.077947
32	1	0	-7.012345	2.338611	-1.707587
33	1	0	-7.653015	1.479158	-0.284673
34	6	0	-2.364366	2.139213	1.253229
35	6	0	-1.202924	1.278595	1.262901
36	6	0	-1.178570	0.039677	1.818341
37	6	0	-2.414412	-0.531178	2.364340
38	6	0	-3.689945	0.141999	1.910236
39	6	0	-3.547593	1.607738	1.606196
40	1	0	-2.258760	3.173924	0.942354
41	1	0	-4.459598	-0.033178	2.668284
42	1	0	-4.452707	2.209504	1.604052
43	6	0	0.952141	0.298209	0.901264
44	6	0	2.247576	0.002054	0.522542
45	6	0	2.777638	-1.221759	0.989525
46	6	0	2.004841	-2.094651	1.743216
47	6	0	0.671651	-1.803012	2.076433
48	6	0	0.157228	-0.577891	1.660928
49	1	0	2.430737	-3.033767	2.082924
50	6	0	0.134046	1.521953	0.614341
51	8	0	0.449110	2.499009	-0.033655
52	8	0	-2.441491	-1.550270	3.048668
53	8	0	-0.014792	-2.734664	2.768770
54	6	0	4.201494	-1.584986	0.753032
55	6	0	3.043338	0.973570	-0.307768
56	1	0	2.444598	1.286033	-1.169796
57	6	0	4.362838	0.433579	-0.804865
58	6	0	4.970686	-0.804793	-0.277674
59	8	0	4.747696	-2.481861	1.372039
60	8	0	4.279552	-0.774899	-1.537145
61	1	0	5.035621	1.181957	-1.222445
62	6	0	6.449336	-1.046962	-0.339857

63	1	0	6.915795	-0.780425	0.612092
64	1	0	6.651766	-2.101749	-0.542974
65	1	0	6.888613	-0.438264	-1.133275
66	1	0	-0.919936	-2.398858	2.981001
67	8	0	3.307141	2.118492	0.518845
68	6	0	3.313097	3.326238	-0.086529
69	8	0	3.213973	3.460394	-1.286644
70	6	0	3.451919	4.440654	0.923811
71	1	0	4.317019	4.181923	1.545826
72	6	0	3.688278	5.771290	0.222888
73	1	0	3.830442	6.560486	0.966237
74	1	0	4.575077	5.736639	-0.415839
75	1	0	2.827503	6.036827	-0.398531
76	6	0	2.206040	4.480869	1.819792
77	1	0	2.036034	3.520593	2.314233
78	1	0	2.336480	5.247661	2.588432
79	1	0	1.318453	4.733203	1.230914

Final Gibbs Free Energy = Single Point Energy + Thermal correction to Gibbs Free Energy

Single Point Energy calculated under M06-2X/6-311+G**:
SCF Done: E(RM062X) = -2443.13400688 A.U.
SMD-CDS (non-electrostatic) energy (kcal/mol) = 13.04
(included in total energy above)

Thermal corrections calculated under M06-2X/6-31G*:
Zero-point correction= 0.596979 (Hartree/Particle)
Thermal correction to Energy= 0.638548
Thermal correction to Enthalpy= 0.639492
Thermal correction to Gibbs Free Energy= 0.525526

27. M06-2X results for INT-III

Center Number	Atomic Number	Atomic Type	Coordinates (Angstroms) X	Y	Z
1	6	0	-7.106861	-1.229257	-0.971807
2	6	0	-5.925031	-0.818259	-0.383305
3	6	0	-5.689034	0.503718	0.021526
4	6	0	-6.674313	1.474007	-0.173990
5	6	0	-7.880576	1.063057	-0.775081
6	6	0	-8.094837	-0.254984	-1.163926
7	1	0	-7.255971	-2.261748	-1.272331
8	1	0	-8.650611	1.813211	-0.929413
9	1	0	-9.040714	-0.524133	-1.623490
10	6	0	-3.725218	-0.671066	0.546457
11	6	0	-2.433424	-0.910435	1.003346
12	6	0	-1.783284	0.204221	1.584159
13	6	0	-2.373153	1.463182	1.641305
14	6	0	-3.677315	1.725708	1.141457
15	6	0	-4.334210	0.591744	0.607515
16	1	0	-1.822558	2.282992	2.094798
17	6	0	-4.708834	-1.623052	-0.070407
18	8	0	-4.566755	-2.818548	-0.270344
19	8	0	-6.521717	2.766425	0.177191
20	1	0	-5.577628	2.881196	0.597566
21	8	0	-4.210408	2.909957	1.191843
22	6	0	-0.417813	0.045691	2.142099
23	6	0	-1.695211	-2.203088	0.773744
24	1	0	-2.398676	-3.039668	0.758666
25	6	0	-0.668776	-2.456927	1.855959
26	6	0	-0.010412	-1.338331	2.576485
27	8	0	0.372403	0.973588	2.241053
28	8	0	-1.071439	-2.100919	3.166174
29	1	0	-0.107502	-3.388041	1.782706
30	6	0	1.332311	-1.476735	3.232902
31	1	0	2.117731	-1.046639	2.607795
32	1	0	1.330588	-0.947638	4.190514
33	1	0	1.552638	-2.532250	3.408071
34	6	0	-0.956424	-2.235639	-0.651196
35	6	0	0.024980	-1.128427	-0.734194
36	6	0	-0.257387	0.080266	-1.261425
37	6	0	-1.544487	0.299597	-1.953451
38	6	0	-2.296551	-0.919155	-2.283584
39	6	0	-2.000188	-2.099042	-1.715432
40	1	0	-0.457070	-3.209020	-0.708578
41	1	0	-3.118868	-0.802942	-2.983092
42	1	0	-2.593624	-2.979502	-1.942041
43	6	0	1.931508	0.289104	-0.479435
44	6	0	3.157060	0.845821	-0.176226
45	6	0	3.332466	2.205627	-0.515225
46	6	0	2.303492	2.937236	-1.092104
47	6	0	1.052983	2.359175	-1.378335
48	6	0	0.888231	1.009734	-1.083156
49	1	0	2.460644	3.983011	-1.337431
50	6	0	1.438272	-1.108607	-0.238285
51	8	0	2.049794	-2.047357	0.231740
52	8	0	-1.937048	1.423045	-2.276442
53	8	0	0.105577	3.146965	-1.922696
54	6	0	4.645771	2.877360	-0.314463
55	6	0	4.250690	0.002500	0.422766
56	1	0	3.848717	-0.586057	1.252528
57	6	0	5.425302	0.794181	0.939655
58	6	0	5.645912	2.212483	0.591962
59	8	0	4.921777	3.928939	-0.864648
60	8	0	5.067803	1.819385	1.848612
61	1	0	6.297626	0.204728	1.219398
62	6	0	7.008520	2.835067	0.656184

63	1	0	7.641215	2.266308	1.340859
64	1	0	7.468970	2.837121	-0.335068
65	1	0	6.936765	3.867851	1.007037
66	1	0	-0.727102	2.625102	-2.047780
67	8	0	4.695133	-0.899344	-0.602870
68	6	0	5.052655	-2.141762	-0.210726
69	8	0	5.130445	-2.463570	0.955296
70	6	0	5.312464	-3.043116	-1.393027
71	1	0	5.704114	-3.972908	-0.971913
72	6	0	3.991053	-3.326581	-2.117565
73	1	0	4.176310	-4.003730	-2.956441
74	1	0	3.259868	-3.793105	-1.451143
75	1	0	3.561835	-2.399765	-2.511820
76	6	0	6.344340	-2.426514	-2.340210
77	1	0	7.272831	-2.178685	-1.817339
78	1	0	6.576417	-3.139657	-3.136130
79	1	0	5.951526	-1.515568	-2.800987

Final Gibbs Free Energy = Single Point Energy + Thermal correction to Gibbs Free Energy

Single Point Energy calculated under M06-2X/6-311+G**:
SCF Done: E(RM062X) = -2443.12662833   A.U.
 SMD-CDS (non-electrostatic) energy   (kcal/mol) =   13.87
 (included in total energy above)

Thermal corrections calculated under M06-2X/6-31G*:
Zero-point correction=   0.596321 (Hartree/Particle)
 Thermal correction to Energy=   0.638508
 Thermal correction to Enthalpy=   0.639452
 Thermal correction to Gibbs Free Energy=   0.520779

28. M06-2X results for INT-IV

Center Number	Atomic Number	Atomic Type	Coordinates (Angstroms) X	Y	Z
1	6	0	3.253907	4.517381	0.771673
2	6	0	2.910639	3.276296	0.268251
3	6	0	1.871822	3.085532	-0.655134
4	6	0	1.163650	4.188446	-1.137939
5	6	0	1.492303	5.453573	-0.612315
6	6	0	2.511270	5.616260	0.320344
7	1	0	4.065320	4.630456	1.483791
8	1	0	0.935284	6.313927	-0.972595
9	1	0	2.734150	6.611446	0.691984
10	6	0	2.781059	0.952299	-0.286506
11	6	0	2.963049	-0.419126	-0.405231
12	6	0	2.097968	-1.074656	-1.313194
13	6	0	1.078279	-0.396667	-1.978967
14	6	0	0.849042	0.996832	-1.826873
15	6	0	1.776648	1.648073	-0.981832
16	1	0	0.435543	-0.951746	-2.657074
17	6	0	3.554215	1.954196	0.523120
18	8	0	4.537912	1.758255	1.217876
19	8	0	0.212242	4.096685	-2.086076
20	1	0	0.037619	3.088713	-2.279303
21	8	0	-0.124862	1.612603	-2.436265
22	6	0	2.269463	-2.521670	-1.594041
23	6	0	3.917591	-1.204992	0.461373
24	1	0	4.777166	-0.584055	0.726864
25	6	0	4.417588	-2.446620	-0.240591
26	6	0	3.606815	-3.136418	-1.264415
27	8	0	1.388882	-3.208685	-2.090807
28	8	0	4.719656	-2.301299	-1.619070
29	1	0	5.122846	-3.059760	0.319819
30	6	0	3.750003	-4.605746	-1.525991
31	1	0	3.012601	-5.161561	-0.940057
32	1	0	3.584760	-4.824967	-2.584359
33	1	0	4.751768	-4.936636	-1.242902
34	6	0	1.775744	0.175193	2.713249
35	6	0	0.686508	-0.503537	2.059155
36	6	0	0.760649	-1.746668	1.514398
37	6	0	2.033038	-2.460050	1.529083
38	6	0	3.254973	-1.626725	1.851925
39	6	0	2.995704	-0.387049	2.651607
40	1	0	1.588442	1.113531	3.225368
41	1	0	3.988076	-2.270442	2.350342
42	1	0	3.857418	0.088414	3.108606
43	6	0	-1.441201	-1.077862	1.125657
44	6	0	-2.751853	-1.105184	0.691424
45	6	0	-3.173096	-2.271697	0.020748
46	6	0	-2.305757	-3.343403	-0.154081
47	6	0	-0.981927	-3.311897	0.313783
48	6	0	-0.549080	-2.148151	0.947678
49	1	0	-2.650371	-4.233679	-0.671180
50	6	0	-0.704227	0.022122	1.825407
51	8	0	-1.105736	1.126787	2.127232
52	8	0	2.143345	-3.643410	1.203683
53	8	0	-0.225725	-4.411464	0.116009
54	6	0	-4.533054	-2.359914	-0.575527
55	6	0	-3.631272	0.101416	0.875768
56	1	0	-3.556955	0.456648	1.908120
57	6	0	-5.087604	-0.145226	0.573178
58	6	0	-5.560411	-1.349388	-0.139749
59	8	0	-4.823131	-3.211559	-1.397732
60	8	0	-5.640032	-1.234136	1.291645
61	1	0	-5.711546	0.746324	0.524359
62	6	0	-6.867166	-1.365862	-0.875622

63	1	0	-6.703021	-1.169844	-1.938225
64	1	0	-7.345899	-2.343277	-0.773960
65	1	0	-7.529398	-0.598396	-0.469191
66	1	0	0.674729	-4.266325	0.494084
67	8	0	-3.113084	1.118481	0.007260
68	6	0	-3.135879	2.388222	0.470881
69	8	0	-3.739311	2.708836	1.471752
70	6	0	-2.331628	3.299993	-0.420077
71	1	0	-1.336945	2.842548	-0.488977
72	6	0	-2.944727	3.320658	-1.825945
73	1	0	-2.351933	3.976534	-2.468801
74	1	0	-2.958516	2.322596	-2.270360
75	1	0	-3.970004	3.704369	-1.791258
76	6	0	-2.224041	4.691974	0.184668
77	1	0	-1.761238	4.659093	1.175359
78	1	0	-1.612201	5.326415	-0.463188
79	1	0	-3.212973	5.151141	0.282657

Final Gibbs Free Energy = Single Point Energy + Thermal correction to Gibbs Free Energy

Single Point Energy calculated under M06-2X/6-311+G**:
SCF Done: E(RM062X) = -2443.13169661   A.U.
SMD-CDS (non-electrostatic) energy   (kcal/mol) =   13.08
(included in total energy above)

Thermal corrections calculated under M06-2X/6-31G*:
Zero-point correction=   0.596300 (Hartree/Particle)
Thermal correction to Energy=   0.638182
Thermal correction to Enthalpy=   0.639127
Thermal correction to Gibbs Free Energy=   0.523080

29. M06-2X results for INT-V

Center Number	Atomic Number	Atomic Type	Coordinates (Angstroms) X	Y	Z
1	6	0	-0.261126	3.004646	-1.089160
2	6	0	0.909319	2.287094	-0.923002
3	6	0	2.055431	2.813705	-0.309043
4	6	0	2.041292	4.122455	0.178410
5	6	0	0.847171	4.854840	0.029625
6	6	0	-0.277049	4.312273	-0.587845
7	1	0	-1.131806	2.556986	-1.561980
8	1	0	0.823250	5.870183	0.414545
9	1	0	-1.175258	4.916033	-0.673473
10	6	0	2.556354	0.568647	-0.749496
11	6	0	3.256924	-0.631023	-0.743069
12	6	0	4.624477	-0.535554	-0.391611
13	6	0	5.184170	0.666088	0.041205
14	6	0	4.431862	1.863623	0.198428
15	6	0	3.099721	1.770741	-0.265458
16	1	0	6.242051	0.693163	0.287173
17	6	0	1.185362	0.868786	-1.277045
18	8	0	0.450829	0.127653	-1.913481
19	8	0	3.101429	4.694154	0.782288
20	1	0	3.888305	4.017688	0.769898
21	8	0	4.949276	2.953935	0.681536
22	6	0	5.525261	-1.696406	-0.595575
23	6	0	2.548222	-1.957791	-0.815561
24	1	0	1.763193	-1.935043	-1.574855
25	6	0	3.463021	-3.125356	-1.129213
26	6	0	4.930146	-3.003592	-1.080158
27	8	0	6.734909	-1.625472	-0.434246
28	8	0	4.204454	-3.675559	-0.039552
29	1	0	3.049497	-3.884353	-1.790195
30	6	0	5.834666	-3.872629	-1.904297
31	1	0	6.150972	-3.343039	-2.807156
32	1	0	6.725144	-4.140984	-1.330084
33	1	0	5.306438	-4.784127	-2.193390
34	6	0	1.805869	-2.223540	0.589811
35	6	0	0.568557	-1.414585	0.666277
36	6	0	0.409519	-0.298184	1.408576
37	6	0	1.484916	0.146724	2.313044
38	6	0	2.588837	-0.798666	2.495847
39	6	0	2.723137	-1.895045	1.732741
40	1	0	1.557134	-3.292549	0.591349
41	1	0	3.304113	-0.553825	3.274922
42	1	0	3.569269	-2.554939	1.887843
43	6	0	-1.588851	-0.482580	0.198728
44	6	0	-2.825590	-0.145752	-0.309827
45	6	0	-3.382897	1.078919	0.127322
46	6	0	-2.729610	1.887623	1.039560
47	6	0	-1.466152	1.534209	1.550218
48	6	0	-0.908334	0.330801	1.127105
49	1	0	-3.164470	2.838128	1.335197
50	6	0	-0.672030	-1.627151	-0.150083
51	8	0	-0.848666	-2.531465	-0.938516
52	8	0	1.454970	1.232927	2.901020
53	8	0	-0.869090	2.413220	2.376013
54	6	0	-4.651395	1.565796	-0.481679
55	6	0	-3.644951	-1.016286	-1.227818
56	1	0	-3.047926	-1.815286	-1.667731
57	6	0	-4.355285	-0.192998	-2.280748
58	6	0	-4.927955	1.113023	-1.893761
59	8	0	-5.414303	2.320636	0.093639
60	8	0	-3.730397	1.009042	-2.683273
61	1	0	-4.857299	-0.749561	-3.069229
62	6	0	-6.140796	1.681819	-2.566599

63	1	0	-7.039016	1.414308	-2.004509
64	1	0	-6.074442	2.771929	-2.616876
65	1	0	-6.223012	1.279492	-3.578506
66	1	0	0.033628	2.079390	2.618388
67	8	0	-4.750122	-1.584460	-0.488437
68	6	0	-4.478824	-2.562621	0.397205
69	8	0	-3.381691	-3.064775	0.507034
70	6	0	-5.689521	-2.885151	1.239369
71	1	0	-6.543242	-2.963634	0.557430
72	6	0	-5.488607	-4.193191	1.991828
73	1	0	-6.387680	-4.431814	2.566297
74	1	0	-5.287034	-5.021766	1.307083
75	1	0	-4.648462	-4.111778	2.688392
76	6	0	-5.930454	-1.706470	2.195072
77	1	0	-6.097363	-0.772656	1.650649
78	1	0	-6.809136	-1.910581	2.812949
79	1	0	-5.068465	-1.573497	2.857743

Final Gibbs Free Energy = Single Point Energy + Thermal correction to Gibbs Free Energy

Single Point Energy calculated under M06-2X/6-311+G**:
SCF Done: E(RM062X) = -2443.11934070   A.U.
 SMD-CDS (non-electrostatic) energy   (kcal/mol) =   14.28
 (included in total energy above)

Thermal corrections calculated under M06-2X/6-31G*:
Zero-point correction=   0.596216 (Hartree/Particle)
 Thermal correction to Energy=   0.638198
 Thermal correction to Enthalpy=   0.639142
 Thermal correction to Gibbs Free Energy=   0.521780

30. M06-2X results for INT-VI

Center Number	Atomic Number	Atomic Type	Coordinates (Angstroms) X	Y	Z
1	6	0	1.309914	-0.600699	2.494198
2	6	0	0.108694	-0.112927	2.013285
3	6	0	-0.140540	1.252994	1.816100
4	6	0	0.843438	2.190272	2.139507
5	6	0	2.085092	1.697803	2.590895
6	6	0	2.316420	0.336605	2.762951
7	1	0	1.463117	-1.663864	2.651602
8	1	0	2.864359	2.420479	2.816798
9	1	0	3.286509	0.001718	3.118646
10	6	0	-2.083737	0.149056	1.087452
11	6	0	-3.363894	-0.032208	0.571323
12	6	0	-4.092321	1.156779	0.330084
13	6	0	-3.521800	2.417121	0.514009
14	6	0	-2.187062	2.613460	0.952657
15	6	0	-1.503532	1.417116	1.268133
16	1	0	-4.132103	3.296271	0.328227
17	6	0	-1.116455	-0.866478	1.623879
18	8	0	-1.298493	-2.064876	1.772058
19	8	0	0.661204	3.519758	2.036502
20	1	0	-0.292845	3.691369	1.655664
21	8	0	-1.667381	3.799759	1.088735
22	6	0	-5.543348	1.110542	0.021109
23	6	0	-3.894839	-1.393058	0.170941
24	1	0	-3.614237	-2.133002	0.925356
25	6	0	-5.404118	-1.442334	0.032834
26	6	0	-6.238767	-0.232863	0.002175
27	8	0	-6.209077	2.118589	-0.164001
28	8	0	-5.944539	-0.961217	-1.199793
29	1	0	-5.873458	-2.363990	0.371713
30	6	0	-7.676098	-0.236431	0.434164
31	1	0	-7.763318	0.120836	1.463987
32	1	0	-8.265418	0.420452	-0.210773
33	1	0	-8.076924	-1.250824	0.374065
34	6	0	-1.928694	-0.241805	-2.510909
35	6	0	-0.751444	-0.839680	-1.938736
36	6	0	-0.738232	-1.968491	-1.182189
37	6	0	-1.992830	-2.662084	-0.904767
38	6	0	-3.265497	-1.896993	-1.209400
39	6	0	-3.125252	-0.754547	-2.175013
40	1	0	-1.824771	0.611122	-3.173429
41	1	0	-3.976265	-2.632997	-1.604557
42	1	0	-4.045115	-0.320483	-2.551746
43	6	0	1.477740	-1.229630	-1.161561
44	6	0	2.799787	-1.109206	-0.779980
45	6	0	3.285572	-2.079297	0.120359
46	6	0	2.478222	-3.128948	0.544317
47	6	0	1.139314	-3.243342	0.139096
48	6	0	0.634944	-2.252688	-0.700640
49	1	0	2.872825	-3.858689	1.244875
50	6	0	0.660335	-0.318768	-2.022819
51	8	0	1.028417	0.642426	-2.664674
52	8	0	-2.047467	-3.787852	-0.410411
53	8	0	0.419084	-4.278053	0.617516
54	6	0	4.625859	-1.926897	0.744284
55	6	0	3.622088	0.059420	-1.250964
56	1	0	3.533409	0.152616	-2.336959
57	6	0	5.088071	-0.040870	-0.908218
58	6	0	5.612075	-1.009252	0.076615
59	8	0	4.923537	-2.502766	1.777046
60	8	0	5.702220	-1.241621	-1.339832
61	1	0	5.667733	0.866078	-1.076816
62	6	0	6.906721	-0.775420	0.795861

63	1	0	6.720966	-0.309147	1.766795
64	1	0	7.425134	-1.723728	0.960110
65	1	0	7.543115	-0.117047	0.200668
66	1	0	-0.503501	-4.228786	0.263657
67	8	0	3.073777	1.244495	-0.646209
68	6	0	3.140745	2.374725	-1.383472
69	8	0	3.718835	2.427973	-2.448186
70	6	0	2.396074	3.515228	-0.736593
71	1	0	2.534927	3.424711	0.345713
72	6	0	2.936302	4.851571	-1.231914
73	1	0	2.415734	5.668273	-0.724393
74	1	0	4.007820	4.949402	-1.034955
75	1	0	2.773652	4.956732	-2.308950
76	6	0	0.900285	3.365531	-1.052375
77	1	0	0.491329	2.436285	-0.641936
78	1	0	0.351169	4.204334	-0.617521
79	1	0	0.739810	3.369129	-2.135916

Final Gibbs Free Energy = Single Point Energy + Thermal correction to Gibbs Free Energy

Single Point Energy calculated under M06-2X/6-311+G**:
SCF Done: E(RM062X) = -2443.12646391   A.U.
 SMD-CDS (non-electrostatic) energy   (kcal/mol) =   12.89
 (included in total energy above)

Thermal corrections calculated under M06-2X/6-31G*:
Zero-point correction=   0.597430 (Hartree/Particle)
 Thermal correction to Energy=   0.638784
 Thermal correction to Enthalpy=   0.639728
 Thermal correction to Gibbs Free Energy=   0.526830

31. M06-2X results for INT-VII

Center Number	Atomic Number	Atomic Type	Coordinates (Angstroms) X	Y	Z
1	6	0	-0.597801	1.572583	3.196680
2	6	0	0.436583	0.869617	2.605147
3	6	0	0.323478	-0.471612	2.200014
4	6	0	-0.887085	-1.150027	2.379721
5	6	0	-1.944325	-0.438738	2.978835
6	6	0	-1.807225	0.890744	3.373019
7	1	0	-0.476067	2.610617	3.490222
8	1	0	-2.888960	-0.955671	3.121350
9	1	0	-2.656410	1.396733	3.821859
10	6	0	2.438886	0.221192	1.483592
11	6	0	3.620469	0.210923	0.756404
12	6	0	4.022367	-1.048855	0.256347
13	6	0	3.212230	-2.176176	0.382934
14	6	0	1.910400	-2.129990	0.954393
15	6	0	1.580931	-0.888192	1.554571
16	1	0	3.556452	-3.121836	-0.025911
17	6	0	1.803702	1.344969	2.249023
18	8	0	2.312051	2.403525	2.578325
19	8	0	-1.080895	-2.424337	1.998466
20	1	0	-0.210989	-2.772348	1.555056
21	8	0	1.103514	-3.145480	0.921707
22	6	0	5.352417	-1.201313	-0.382857
23	6	0	4.193839	1.498981	0.211913
24	1	0	4.227338	2.251586	1.002285
25	6	0	5.596634	1.351496	-0.344423
26	6	0	6.204443	0.033355	-0.594629
27	8	0	5.800322	-2.289945	-0.710676
28	8	0	5.707529	0.842973	-1.671470
29	1	0	6.274952	2.178373	-0.143448
30	6	0	7.689302	-0.183557	-0.575131
31	1	0	8.001144	-0.591027	0.390514
32	1	0	7.975407	-0.891637	-1.357239
33	1	0	8.203654	0.765356	-0.743252
34	6	0	1.053068	3.162941	-0.234016
35	6	0	0.373522	1.937990	-0.569727
36	6	0	0.968489	0.864274	-1.155551
37	6	0	2.382924	0.931089	-1.525731
38	6	0	3.200811	2.070014	-0.922251
39	6	0	2.388107	3.211934	-0.383824
40	1	0	0.485208	3.987555	0.184676
41	1	0	3.842550	2.449172	-1.727379
42	1	0	2.948961	4.092078	-0.080644
43	6	0	-1.219576	0.163250	-0.732895
44	6	0	-2.321537	-0.661146	-0.618883
45	6	0	-2.177758	-1.980515	-1.096181
46	6	0	-0.971528	-2.415990	-1.633137
47	6	0	0.143898	-1.568697	-1.730548
48	6	0	0.008136	-0.258619	-1.272222
49	1	0	-0.877474	-3.438305	-1.985968
50	6	0	-1.058095	1.581154	-0.279733
51	8	0	-1.891715	2.305271	0.228449
52	8	0	2.908575	0.086744	-2.249468
53	8	0	1.278309	-2.084047	-2.243641
54	6	0	-3.319143	-2.930841	-1.060347
55	6	0	-3.603990	-0.136194	-0.032113
56	1	0	-3.380381	0.439877	0.869875
57	6	0	-4.595704	-1.211721	0.331542
58	6	0	-4.482249	-2.598543	-0.164455
59	8	0	-3.334060	-3.951932	-1.726891
60	8	0	-4.062875	-2.228237	1.160978
61	1	0	-5.588326	-0.856923	0.606904
62	6	0	-5.676255	-3.501922	-0.251597

63	1	0	-6.082743	-3.491929	-1.266072
64	1	0	-5.394351	-4.528097	-0.001645
65	1	0	-6.446239	-3.161581	0.444209
66	1	0	1.961060	-1.373056	-2.308666
67	8	0	-4.187883	0.748351	-1.002695
68	6	0	-4.815708	1.845062	-0.524660
69	8	0	-5.034250	2.015654	0.655205
70	6	0	-5.162746	2.804273	-1.637983
71	1	0	-5.636917	2.211625	-2.428597
72	6	0	-6.115940	3.882383	-1.140617
73	1	0	-6.376737	4.553085	-1.963835
74	1	0	-7.038111	3.450331	-0.742335
75	1	0	-5.645188	4.474828	-0.350198
76	6	0	-3.863549	3.405270	-2.194491
77	1	0	-3.182629	2.627803	-2.551878
78	1	0	-4.098856	4.070182	-3.030037
79	1	0	-3.351857	3.989805	-1.422962

Final Gibbs Free Energy = Single Point Energy + Thermal correction to Gibbs Free Energy

Single Point Energy calculated under M06-2X/6-311+G**:
SCF Done: E(RM062X) = -2443.12123846 A.U.
SMD-CDS (non-electrostatic) energy (kcal/mol) = 13.13
(included in total energy above)

Thermal corrections calculated under M06-2X/6-31G*:
Zero-point correction= 0.596537 (Hartree/Particle)
Thermal correction to Energy= 0.637975
Thermal correction to Enthalpy= 0.638920
Thermal correction to Gibbs Free Energy= 0.526014

32. M06-2X results for INT-VIII

Center Number	Atomic Number	Atomic Type	Coordinates (Angstroms) X	Y	Z
1	6	0	-0.040660	-3.213746	2.552997
2	6	0	-0.576390	-1.978014	2.240025
3	6	0	0.180796	-0.794610	2.238297
4	6	0	1.548314	-0.842426	2.508956
5	6	0	2.102035	-2.098590	2.828894
6	6	0	1.327342	-3.254455	2.856570
7	1	0	-0.648527	-4.113448	2.531954
8	1	0	3.167308	-2.145702	3.036605
9	1	0	1.801385	-4.201620	3.094589
10	6	0	-1.895035	-0.204821	1.337901
11	6	0	-2.834703	0.552331	0.646335
12	6	0	-2.583730	1.938100	0.605396
13	6	0	-1.409654	2.486401	1.130554
14	6	0	-0.368618	1.696413	1.680502
15	6	0	-0.666148	0.316052	1.763928
16	1	0	-1.269920	3.562984	1.088297
17	6	0	-1.940821	-1.650003	1.732106
18	8	0	-2.900764	-2.401710	1.677105
19	8	0	2.350273	0.239162	2.440379
20	1	0	1.757334	1.072842	2.296567
21	8	0	0.752454	2.199017	2.103317
22	6	0	-3.616207	2.867550	0.088952
23	6	0	-3.907737	-0.129319	-0.169478
24	1	0	-4.488610	-0.786702	0.483950
25	6	0	-4.862633	0.831150	-0.841048
26	6	0	-4.764175	2.297290	-0.706262
27	8	0	-3.577004	4.070853	0.298780
28	8	0	-4.293966	1.619134	-1.882777
29	1	0	-5.844303	0.428630	-1.088171
30	6	0	-5.952350	3.193385	-0.894860
31	1	0	-6.384000	3.458858	0.073922
32	1	0	-5.653646	4.114318	-1.402269
33	1	0	-6.709852	2.682866	-1.493683
34	6	0	-3.280008	-1.051675	-1.327533
35	6	0	-1.811013	-1.264140	-1.242345
36	6	0	-1.201721	-2.445350	-1.010916
37	6	0	-2.002612	-3.673683	-0.859829
38	6	0	-3.435658	-3.548514	-1.147382
39	6	0	-4.014175	-2.356852	-1.355458
40	1	0	-3.472620	-0.520159	-2.270119
41	1	0	-4.009128	-4.469664	-1.131268
42	1	0	-5.088452	-2.297230	-1.517182
43	6	0	0.559494	-0.902383	-1.160576
44	6	0	1.848751	-0.402411	-1.167639
45	6	0	2.881505	-1.317403	-0.864606
46	6	0	2.609599	-2.637526	-0.543769
47	6	0	1.296489	-3.140267	-0.567883
48	6	0	0.272021	-2.257078	-0.901720
49	1	0	3.423575	-3.316313	-0.306887
50	6	0	-0.749204	-0.208973	-1.417697
51	8	0	-0.940522	0.943749	-1.743416
52	8	0	-1.506221	-4.752531	-0.520003
53	8	0	1.127485	-4.443232	-0.272464
54	6	0	4.303453	-0.887354	-0.946015
55	6	0	2.194424	1.028502	-1.484750
56	1	0	1.383958	1.511347	-2.028361
57	6	0	3.501947	1.134650	-2.243895
58	6	0	4.610305	0.212494	-1.927948
59	8	0	5.190212	-1.414827	-0.298353
60	8	0	3.835127	0.090743	-3.135475
61	1	0	3.765431	2.139053	-2.573573
62	6	0	6.048298	0.608172	-2.076780

63	1	0	6.432965	0.978768	-1.123089
64	1	0	6.651348	-0.251280	-2.381821
65	1	0	6.139733	1.396624	-2.826791
66	1	0	0.161478	-4.667254	-0.308552
67	8	0	2.463667	1.766845	-0.273014
68	6	0	1.911100	2.999302	-0.155140
69	8	0	0.922046	3.326792	-0.776336
70	6	0	2.763247	3.944779	0.666800
71	1	0	3.468701	4.324577	-0.090052
72	6	0	1.937331	5.119283	1.180515
73	1	0	2.593492	5.853002	1.657086
74	1	0	1.400321	5.615127	0.368294
75	1	0	1.208819	4.774473	1.920304
76	6	0	3.588195	3.285142	1.770195
77	1	0	4.153298	2.427008	1.398103
78	1	0	4.300922	4.014490	2.166430
79	1	0	2.948321	2.954035	2.591416

Final Gibbs Free Energy = Single Point Energy + Thermal correction to Gibbs Free Energy

Single Point Energy calculated under M06-2X/6-311+G**:
SCF Done: E(RM062X) = -2443.12275555 A.U.
SMD-CDS (non-electrostatic) energy (kcal/mol) = 12.54
(included in total energy above)

Thermal corrections calculated under M06-2X/6-31G*:
Zero-point correction= 0.597444 (Hartree/Particle)
Thermal correction to Energy= 0.638717
Thermal correction to Enthalpy= 0.639661
Thermal correction to Gibbs Free Energy= 0.527884

33. M06-2X results for Prod-I/III

Center Number	Atomic Number	Atomic Type	Coordinates (Angstroms) X	Y	Z
1	6	0	2.109107	3.750840	-1.437135
2	6	0	2.712666	2.592462	-0.995312
3	6	0	4.100478	2.358645	-1.051638
4	6	0	4.918132	3.353131	-1.590863
5	6	0	4.311433	4.541263	-2.044895
6	6	0	2.942554	4.742799	-1.971221
7	1	0	1.032691	3.880207	-1.370247
8	1	0	4.961962	5.305174	-2.459498
9	1	0	2.521794	5.675369	-2.332382
10	6	0	3.142705	0.450877	-0.042560
11	6	0	3.046809	-0.781814	0.592054
12	6	0	4.255550	-1.476626	0.789242
13	6	0	5.470109	-0.950903	0.349370
14	6	0	5.535427	0.292663	-0.274245
15	6	0	4.360346	1.016743	-0.462851
16	1	0	6.384245	-1.517856	0.505978
17	6	0	2.047621	1.402428	-0.398154
18	8	0	0.848922	1.240662	-0.256651
19	8	0	6.265641	3.285978	-1.718770
20	1	0	6.595849	2.425094	-1.387751
21	8	0	6.715806	0.821556	-0.702914
22	6	0	4.272338	-2.807521	1.450559
23	6	0	1.711684	-1.350876	1.020237
24	1	0	1.108827	-0.534392	1.425361
25	6	0	1.859286	-2.379764	2.120533
26	6	0	3.102863	-3.150360	2.329786
27	8	0	5.194674	-3.594217	1.309276
28	8	0	2.736228	-2.053306	3.185903
29	1	0	0.931724	-2.860894	2.430549
30	6	0	3.101009	-4.515323	2.950590
31	1	0	3.097039	-5.283072	2.172363
32	1	0	3.992061	-4.654536	3.568942
33	1	0	2.210813	-4.634883	3.572122
34	6	0	0.940581	-1.975257	-0.133265
35	6	0	-0.418544	-1.764294	-0.323636
36	6	0	-1.139732	-2.372154	-1.368108
37	6	0	-0.534540	-3.258490	-2.283125
38	6	0	0.860179	-3.475433	-2.061609
39	6	0	1.560221	-2.861302	-1.039413
40	1	0	7.460386	0.224790	-0.513691
41	1	0	1.373138	-4.151298	-2.740631
42	1	0	2.623837	-3.068762	-0.938668
43	6	0	-2.687318	-1.005047	-0.257670
44	6	0	-3.882901	-0.356291	0.020057
45	6	0	-4.964400	-0.629753	-0.835476
46	6	0	-4.829969	-1.540203	-1.889740
47	6	0	-3.625112	-2.199512	-2.154343
48	6	0	-2.534442	-1.902206	-1.322088
49	1	0	-5.681628	-1.734341	-2.534580
50	6	0	-1.369078	-0.897193	0.445248
51	8	0	-1.142337	-0.248605	1.455141
52	8	0	-1.168807	-3.840721	-3.259715
53	8	0	-3.549658	-3.065090	-3.178221
54	6	0	-6.248937	0.096354	-0.697590
55	6	0	-3.951291	0.645934	1.140138
56	1	0	-3.534459	0.209307	2.052231
57	6	0	-5.340461	1.137685	1.453595
58	6	0	-6.491366	0.880751	0.566527
59	8	0	-7.101850	0.084765	-1.572346
60	8	0	-6.258204	0.099403	1.751974
61	1	0	-5.390351	2.006532	2.109216
62	6	0	-7.707958	1.758068	0.588078

63	1	0	-7.639820	2.518647	-0.194045
64	1	0	-8.607867	1.162683	0.413342
65	1	0	-7.787917	2.252480	1.558707
66	1	0	-2.564113	-3.424194	-3.247675
67	8	0	-3.116506	1.751843	0.752887
68	6	0	-2.372963	2.322956	1.722826
69	8	0	-2.547504	2.104420	2.902972
70	6	0	-1.349078	3.273284	1.151552
71	1	0	-1.080454	2.899917	0.159269
72	6	0	-1.995119	4.657905	1.010441
73	1	0	-1.270093	5.359893	0.588609
74	1	0	-2.868891	4.626228	0.353353
75	1	0	-2.308930	5.032881	1.990169
76	6	0	-0.112923	3.311336	2.044467
77	1	0	0.296828	2.308087	2.197728
78	1	0	0.659254	3.932357	1.579641
79	1	0	-0.356956	3.736273	3.023100

```
Final Gibbs Free Energy = Single Point Energy + Thermal correction to Gibbs Free Energy

Single Point Energy calculated under M06-2X/6-311+G**:
SCF Done:  E(RM062X) =  -2443.16195898     A.U.
 SMD-CDS (non-electrostatic) energy       (kcal/mol) =      13.82
 (included in total energy above)

Thermal corrections calculated under M06-2X/6-31G*:
Zero-point correction=                           0.595280 (Hartree/Particle)
 Thermal correction to Energy=                    0.637967
 Thermal correction to Enthalpy=                  0.638912
 Thermal correction to Gibbs Free Energy=         0.519730
```

34. M06-2X results for Prod-V/VIII

Center Number	Atomic Number	Atomic Type	Coordinates (Angstroms) X	Y	Z
1	6	0	-4.015652	-3.383103	-2.069227
2	6	0	-4.140766	-2.123454	-1.516374
3	6	0	-5.364543	-1.478911	-1.248158
4	6	0	-6.530091	-2.198903	-1.534434
5	6	0	-6.420089	-3.481361	-2.100437
6	6	0	-5.192605	-4.070358	-2.372302
7	1	0	-3.034849	-3.812854	-2.248470
8	1	0	-7.338695	-4.019544	-2.322096
9	1	0	-5.159209	-5.063729	-2.807393
10	6	0	-3.672540	-0.028342	-0.486804
11	6	0	-3.024832	1.069868	0.069673
12	6	0	-3.864154	2.166341	0.377206
13	6	0	-5.234666	2.101174	0.168478
14	6	0	-5.936009	0.958192	-0.342541
15	6	0	-5.069976	-0.138078	-0.659633
16	1	0	-5.835762	2.977174	0.397206
17	6	0	-3.031840	-1.235810	-1.084222
18	8	0	-1.841065	-1.451464	-1.253132
19	8	0	-7.763545	-1.687377	-1.258260
20	1	0	-7.654524	-0.806835	-0.861667
21	8	0	-7.200679	0.987264	-0.509216
22	6	0	-3.302847	3.466996	0.822032
23	6	0	-1.528766	1.081913	0.306376
24	1	0	-1.009221	1.068369	-0.656456
25	6	0	-1.050951	2.307675	1.058303
26	6	0	-1.882735	3.495950	1.314825
27	8	0	-3.947446	4.505669	0.790956
28	8	0	-1.651162	2.506636	2.333111
29	1	0	0.028396	2.457605	1.054483
30	6	0	-1.270070	4.844734	1.551911
31	1	0	-1.223477	5.410188	0.617261
32	1	0	-1.866387	5.414539	2.269663
33	1	0	-0.257258	4.723915	1.944227
34	6	0	-1.026508	-0.099081	1.128664
35	6	0	0.281862	-0.554931	1.022083
36	6	0	0.800967	-1.608199	1.800588
37	6	0	-0.024314	-2.213026	2.738925
38	6	0	-1.338434	-1.750100	2.884230
39	6	0	-1.827042	-0.714732	2.101922
40	1	0	-0.221652	-3.578794	4.104912
41	1	0	-1.973157	-2.219917	3.631074
42	1	0	-2.851388	-0.385727	2.244872
43	6	0	2.555401	-0.927982	0.401143
44	6	0	3.807937	-0.855639	-0.185935
45	6	0	4.783379	-1.746695	0.296582
46	6	0	4.473120	-2.660028	1.297746
47	6	0	3.196051	-2.736635	1.867659
48	6	0	2.219313	-1.841240	1.416751
49	1	0	5.237856	-3.341228	1.657739
50	6	0	1.362389	-0.072570	0.116297
51	8	0	1.307475	0.847493	-0.682878
52	8	0	0.458843	-3.236385	3.499845
53	8	0	3.022571	-3.679509	2.820916
54	6	0	6.181347	-1.705695	-0.208017
55	6	0	4.101887	0.171370	-1.247276
56	1	0	3.283720	0.197081	-1.971250
57	6	0	5.387837	-0.072794	-1.997386
58	6	0	6.436190	-0.988381	-1.505119
59	8	0	7.102463	-2.229951	0.394902
60	8	0	5.508895	-1.377453	-2.533942
61	1	0	5.699235	0.734469	-2.659385
62	6	0	7.868225	-0.842907	-1.925726

63	1	0	8.428636	-0.286284	-1.170310
64	1	0	8.329530	-1.826776	-2.045154
65	1	0	7.919745	-0.304428	-2.874545
66	1	0	2.102674	-3.662483	3.160569
67	8	0	4.174569	1.452217	-0.596337
68	6	0	3.765075	2.517771	-1.317758
69	8	0	3.461585	2.440393	-2.488899
70	6	0	3.714038	3.770182	-0.476497
71	1	0	4.600629	3.760960	0.166130
72	6	0	3.712298	5.009033	-1.362451
73	1	0	3.702209	5.908417	-0.740791
74	1	0	4.597492	5.043497	-2.003784
75	1	0	2.823953	5.023197	-2.000983
76	6	0	2.462076	3.710073	0.412563
77	1	0	2.465532	2.821331	1.050823
78	1	0	2.427949	4.597212	1.051635
79	1	0	1.558225	3.696484	-0.206803

Final Gibbs Free Energy = Single Point Energy + Thermal correction to Gibbs Free Energy

Single Point Energy calculated under M06-2X/6-311+G**:
SCF Done: E(RM062X) = -2442.45467660 A.U.
SMD-CDS (non-electrostatic) energy (kcal/mol) = 14.21
(included in total energy above)

Thermal corrections calculated under M06-2X/6-31G*:
Zero-point correction= 0.596127 (Hartree/Particle)
Thermal correction to Energy= 0.638577
Thermal correction to Enthalpy= 0.639522
Thermal correction to Gibbs Free Energy= 0.521250

35. M06-2X results for Prod-II/IV

Center Number	Atomic Number	Atomic Type	Coordinates (Angstroms) X	Y	Z
1	6	0	-4.435655	-4.130532	-0.400378
2	6	0	-4.615208	-2.764901	-0.343686
3	6	0	-5.628650	-2.077481	-1.040245
4	6	0	-6.503532	-2.817136	-1.837398
5	6	0	-6.329486	-4.213968	-1.900449
6	6	0	-5.323274	-4.860975	-1.201461
7	1	0	-3.635828	-4.610419	0.154388
8	1	0	-7.018564	-4.775361	-2.523803
9	1	0	-5.226882	-5.938807	-1.281660
10	6	0	-4.415258	-0.444461	0.155580
11	6	0	-4.025029	0.797434	0.640990
12	6	0	-4.764756	1.905928	0.190412
13	6	0	-5.831631	1.748026	-0.694473
14	6	0	-6.208107	0.488894	-1.154419
15	6	0	-5.493570	-0.629139	-0.728120
16	1	0	-6.379418	2.624462	-1.031504
17	6	0	-3.802935	-1.780644	0.424641
18	8	0	-2.832405	-2.031198	1.117001
19	8	0	-7.524825	-2.310332	-2.570450
20	1	0	-7.558490	-1.335666	-2.475905
21	8	0	-7.253174	0.320405	-2.013536
22	6	0	-4.392634	3.284586	0.599175
23	6	0	-2.825230	0.943096	1.542652
24	1	0	-2.848173	0.138640	2.284768
25	6	0	-2.833437	2.243982	2.313073
26	6	0	-3.569091	3.436975	1.846693
27	8	0	-4.740525	4.266675	-0.037237
28	8	0	-4.060369	2.615721	2.921471
29	1	0	-1.952047	2.422953	2.928594
30	6	0	-3.148918	4.830308	2.207909
31	1	0	-2.536522	5.258242	1.409938
32	1	0	-4.025955	5.468161	2.348608
33	1	0	-2.566929	4.813043	3.131921
34	6	0	-0.129742	1.209989	-1.181309
35	6	0	0.915357	0.611303	-0.506905
36	6	0	0.791744	0.122525	0.802683
37	6	0	-0.419108	0.210324	1.518292
38	6	0	-1.506973	0.816436	0.797190
39	6	0	-1.351429	1.301719	-0.495564
40	1	0	-0.012657	1.589740	-2.191066
41	1	0	-7.641418	1.175005	-2.269731
42	1	0	-2.203411	1.761101	-0.992001
43	6	0	3.008002	-0.314296	0.164253
44	6	0	4.315799	-0.767560	0.246552
45	6	0	4.712115	-1.340929	1.468252
46	6	0	3.810084	-1.461109	2.530060
47	6	0	2.483365	-1.028640	2.427261
48	6	0	2.089671	-0.439226	1.217565
49	1	0	4.144633	-1.907160	3.461835
50	6	0	2.312069	0.372839	-0.971389
51	8	0	2.791400	0.683493	-2.050444
52	8	0	-0.570119	-0.211622	2.738702
53	8	0	1.648314	-1.184961	3.469709
54	6	0	6.109384	-1.787455	1.679143
55	6	0	5.252559	-0.594729	-0.917710
56	1	0	4.741514	-0.871035	-1.843776
57	6	0	6.520699	-1.403884	-0.811876
58	6	0	6.972624	-1.995824	0.462389
59	8	0	6.570569	-1.989575	2.791952
60	8	0	6.314202	-2.780250	-0.548285
61	1	0	7.280308	-1.183207	-1.560914
62	6	0	8.416520	-2.317881	0.709309

63	1	0	8.901074	-1.497961	1.245474
64	1	0	8.503689	-3.225373	1.312543
65	1	0	8.927558	-2.469454	-0.243943
66	1	0	0.716467	-0.798215	3.215910
67	8	0	5.595125	0.801907	-0.990458
68	6	0	5.779155	1.319684	-2.222790
69	8	0	5.800517	0.639197	-3.226326
70	6	0	5.923284	2.821847	-2.171795
71	1	0	6.611041	3.045854	-1.348759
72	6	0	6.481108	3.351548	-3.485817
73	1	0	6.610989	4.435376	-3.423547
74	1	0	7.449818	2.900512	-3.718894
75	1	0	5.794818	3.134445	-4.310026
76	6	0	4.554602	3.436552	-1.843697
77	1	0	4.163672	3.063424	-0.892983
78	1	0	4.651275	4.523796	-1.776639
79	1	0	3.832837	3.202868	-2.633195

Final Gibbs Free Energy = Single Point Energy + Thermal correction to Gibbs Free Energy

Single Point Energy calculated under M06-2X/6-311+G**:
SCF Done: E(RM062X) = -2443.16270796   A.U.
SMD-CDS (non-electrostatic) energy   (kcal/mol) =   14.56
(included in total energy above)

Thermal corrections calculated under M06-2X/6-31G*:
Zero-point correction=   0.598316 (Hartree/Particle)
Thermal correction to Energy=   0.637114
Thermal correction to Enthalpy=   0.638058
Thermal correction to Gibbs Free Energy=   0.531164

36. M06-2X results for Prod-VI/VII

Center Number	Atomic Number	Atomic Type	Coordinates (Angstroms) X	Y	Z
1	6	0	-3.484175	3.412867	2.292802
2	6	0	-3.961809	2.355137	1.548737
3	6	0	-5.139079	2.407865	0.778555
4	6	0	-5.867141	3.598851	0.758324
5	6	0	-5.384454	4.687616	1.510940
6	6	0	-4.223348	4.603093	2.262190
7	1	0	-2.570917	3.319619	2.871786
8	1	0	-5.961112	5.607012	1.485336
9	1	0	-3.891005	5.467768	2.827270
10	6	0	-4.235860	0.246737	0.465194
11	6	0	-4.100347	-1.062570	0.008307
12	6	0	-5.167600	-1.561074	-0.762599
13	6	0	-6.256568	-0.752095	-1.093257
14	6	0	-6.331491	0.572153	-0.679487
15	6	0	-5.318173	1.081131	0.130689
16	1	0	-7.067384	-1.171755	-1.682704
17	6	0	-3.357459	0.999738	1.415390
18	8	0	-2.376610	0.591191	2.009605
19	8	0	-7.012879	3.812988	0.066563
20	1	0	-7.281393	2.997764	-0.406985
21	8	0	-7.384156	1.370550	-1.014962
22	6	0	-5.255610	-2.993213	-1.149900
23	6	0	-2.875307	-1.882957	0.341162
24	1	0	-2.823370	-2.016499	1.427864
25	6	0	-2.880649	-3.263843	-0.284118
26	6	0	-4.027174	-3.843956	-1.008698
27	8	0	-6.296370	-3.485064	-1.557072
28	8	0	-2.909036	-3.273115	-1.707212
29	1	0	-2.168514	-3.964713	0.148451
30	6	0	-4.213459	-5.324444	-1.158396
31	1	0	-4.895822	-5.702697	-0.392686
32	1	0	-4.635629	-5.553914	-2.140515
33	1	0	-3.249388	-5.827475	-1.055454
34	6	0	-0.265780	0.143712	-1.629799
35	6	0	0.862676	-0.224261	-0.926479
36	6	0	0.814978	-1.094086	0.175719
37	6	0	-0.396255	-1.640667	0.639293
38	6	0	-1.566563	-1.242002	-0.097020
39	6	0	-1.488776	-0.395157	-1.193924
40	1	0	-0.211620	0.808712	-2.485755
41	1	0	-8.003442	0.905096	-1.603978
42	1	0	-2.399299	-0.137069	-1.728667
43	6	0	3.083369	-0.577626	-0.133614
44	6	0	4.456057	-0.596878	0.063318
45	6	0	4.932777	-1.370100	1.136783
46	6	0	4.050134	-2.091931	1.946681
47	6	0	2.668769	-2.084350	1.727221
48	6	0	2.188332	-1.305584	0.664901
49	1	0	4.445033	-2.681021	2.768976
50	6	0	2.282384	0.161416	-1.163509
51	8	0	2.706126	0.927949	-2.014987
52	8	0	-0.484010	-2.463528	1.644015
53	8	0	1.863486	-2.805271	2.527976
54	6	0	6.376844	-1.405001	1.466652
55	6	0	5.357511	0.228444	-0.813389
56	1	0	5.057405	0.120362	-1.858878
57	6	0	6.818751	-0.123640	-0.698034
58	6	0	7.350810	-0.923114	0.422670
59	8	0	6.792562	-1.808822	2.542009
60	8	0	7.099818	-1.501133	-0.871020
61	1	0	7.504887	0.558685	-1.198988
62	6	0	8.790007	-0.834775	0.835807

63	1	0	8.904635	-0.127109	1.660794
64	1	0	9.150723	-1.812333	1.166404
65	1	0	9.394457	-0.497054	-0.008863
66	1	0	0.881560	-2.695644	2.204689
67	8	0	5.176907	1.604242	-0.430602
68	6	0	5.251060	2.525618	-1.413507
69	8	0	5.590644	2.255335	-2.545826
70	6	0	4.833212	3.888883	-0.917737
71	1	0	5.311451	4.031232	0.057481
72	6	0	5.275560	4.975279	-1.888535
73	1	0	4.990169	5.956596	-1.499881
74	1	0	6.359107	4.964131	-2.036374
75	1	0	4.795758	4.838882	-2.862486
76	6	0	3.310308	3.890138	-0.717956
77	1	0	2.997035	3.112594	-0.015705
78	1	0	2.995614	4.860790	-0.324746
79	1	0	2.800441	3.723340	-1.672519

Final Gibbs Free Energy = Single Point Energy + Thermal correction to Gibbs Free Energy

Single Point Energy calculated under M06-2X/6-311+G**:
SCF Done: E(RM062X) = -2443.15857103 A.U.
SMD-CDS (non-electrostatic) energy (kcal/mol) = 14.55
(included in total energy above)

Thermal corrections calculated under M06-2X/6-31G*:
Zero-point correction= 0.598806 (Hartree/Particle)
Thermal correction to Energy= 0.638476
Thermal correction to Enthalpy= 0.639420
Thermal correction to Gibbs Free Energy= 0.530003
